# Discovery of GLPG3667, a Selective ATP Competitive Tyrosine Kinase 2 Inhibitor for the Treatment of Autoimmune Diseases

**DOI:** 10.1021/acs.jmedchem.4c00769

**Published:** 2024-05-28

**Authors:** Oscar Mammoliti, Sébastien Martina, Pieter Claes, Ghjuvanni Coti, Roland Blanque, Catherine Jagerschmidt, Kenji Shoji, Monica Borgonovi, Steve De Vos, Florence Marsais, Line Oste, Evelyne Quinton, Miriam López-Ramos, David Amantini, Reginald Brys, Juan-Miguel Jimenez, René Galien, Steven van der Plas

**Affiliations:** † Galapagos NV, Generaal De Wittelaan L11, A3, 2800 Mechelen, Belgium; ‡ Galapagos SASU, 102 Avenue Gaston Roussel, 93230 Romainville, France

## Abstract

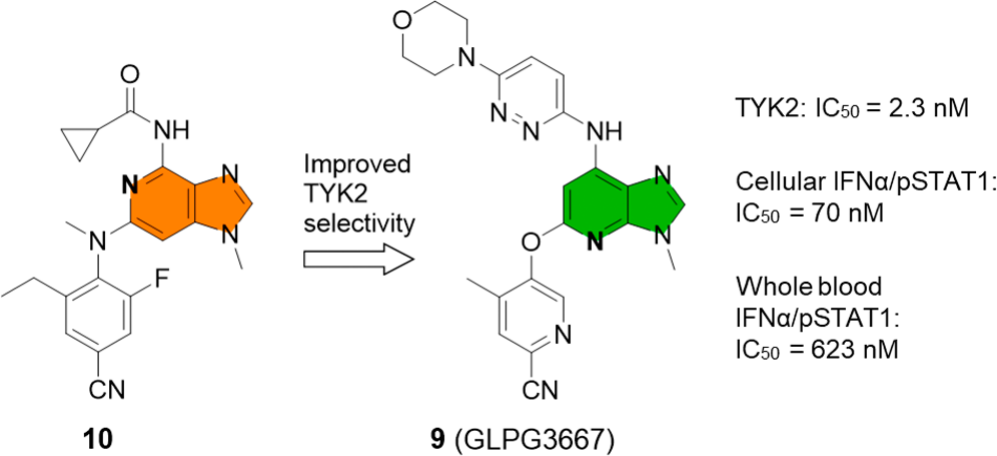

Tyrosine kinase 2 (TYK2) mediates cytokine signaling through type 1 interferon, interleukin (IL)-12/IL-23, and the IL-10 family. There appears to be an association between TYK2 genetic variants and inflammatory conditions, and clinical evidence suggests that selective inhibition of TYK2 could produce a unique therapeutic profile. Here, we describe the discovery of compound **9** (GLPG3667), a reversible and selective TYK2 adenosine triphosphate competitive inhibitor in development for the treatment of inflammatory and autoimmune diseases. The preclinical pharmacokinetic profile was favorable, and TYK2 selectivity was confirmed in peripheral blood mononuclear cells and whole blood assays. Dermal ear inflammation was reduced in an IL-23-induced *in vivo* mouse model of psoriasis. GLPG3667 also completed a phase 1b study (NCT04594928) in patients with moderate-to-severe psoriasis where clinical effect was shown within the 4 weeks of treatment and it is now in phase 2 trials for the treatment of dermatomyositis (NCT05695950) and systemic lupus erythematosus (NCT05856448).

## Introduction

Janus kinases (JAKs) are a class of nonreceptor cytoplasmic tyrosine kinases involved in the signaling of more than 60 cytokines and growth factors. The four members of this class (JAK1, JAK2, JAK3, and tyrosine kinase 2 [TYK2])^1^ function in pairs to phosphorylate signal transducer and activator of transcription (STAT) proteins when activated by a ligand.^2^ Activated STATs dimerize and migrate to the nucleus where they regulate gene transcription.^3^

**Figure 1:**
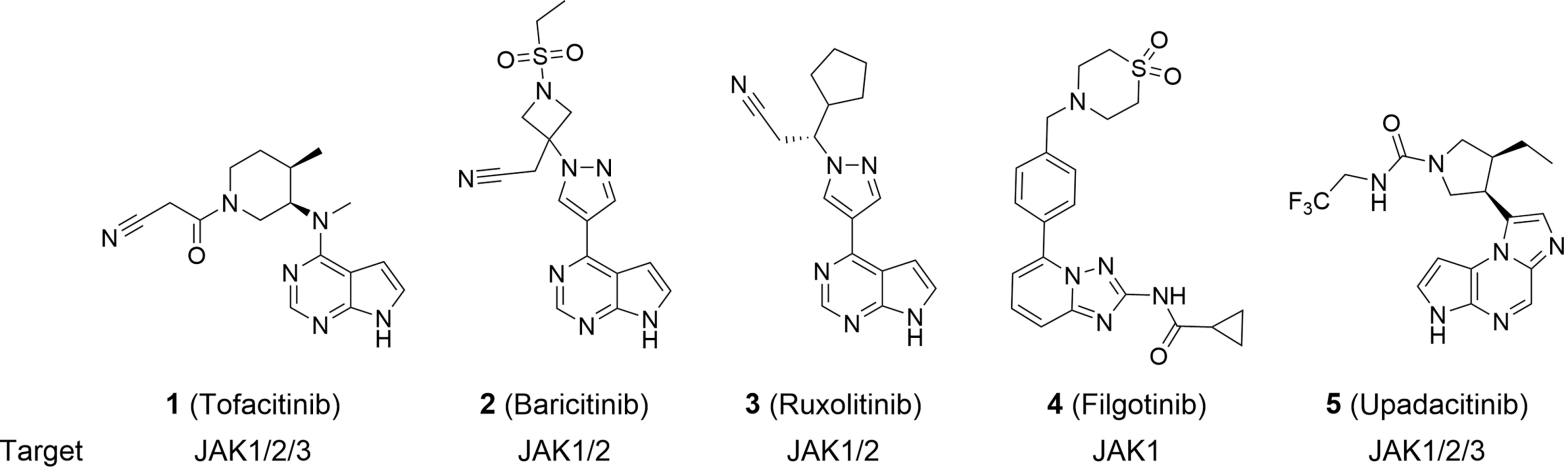
Examples of approved selective and nonselective JAK1 inhibitors.^4^ (Note: This is not an exhaustive list of all licensed JAK inhibitors targeting JAK1.)

During the past decade, a number of JAK inhibitors such as **1** (tofacitinib), **2** (baricitinib), **3** (ruxolitinib), **4** (filgotinib), and **5** (upadacitinib) have been approved to treat patients with inflammatory diseases.^4^ All these molecules inhibit JAK1 (Figure 1), which is considered the key target owing to its major role in JAK/STAT-dependent signaling.^5^ Some of the molecules also target other JAKs, leading to a broader spectrum of activity but with potentially some liabilities.^5^ Notably, all of the current approved JAK inhibitors display a low potency toward TYK2, which is implicated in type I interferon (IFN) and interleukin (IL)-10 family signaling and required for IL-12 and IL-23 signaling.^4,6−10^

IL-12 and IL-23 are cytokines that play an important role in various inflammatory diseases.^11^ The anti-IL-12/23 antibody ustekinumab and anti-IL-23 antibodies (guselkumab, tildrakizumab, and risankizumab) are approved in the USA and European Union for the treatment of plaque psoriasis; some are also approved for psoriatic arthritis, Crohn’s disease, and/or ulcerative colitis.^12−19^ The remarkable efficacy of these antibodies^20,21^ has generated interest in targeting TYK2 owing to its role in IL-12 and IL-23 signaling.^22^

Type I IFN is also a key driver in some inflammatory and autoimmune diseases such as systemic lupus erythematosus (SLE) and dermatomyositis. Anifrolumab is a monoclonal antibody that blocks IFNα/β signaling and has recently been approved for the treatment of patients with SLE.^23^ The TYK2-selective inhibitor **6** deucravacitinib (BMS-986165) has displayed good efficacy in a phase 2 trial in patients with SLE,^24^ linking the interest in type I IFN and TYK2 in this class of disease. Regarding dermatomyositis, several reports highlight the important role of type I and type III IFN in disease progression and severity.^25−28^ In addition, several genetic studies have identified associations between TYK2 gene single nucleotide polymorphisms and an increased or decreased risk of developing inflammatory or autoimmune diseases.^8,29,30^

**Figure 2:**
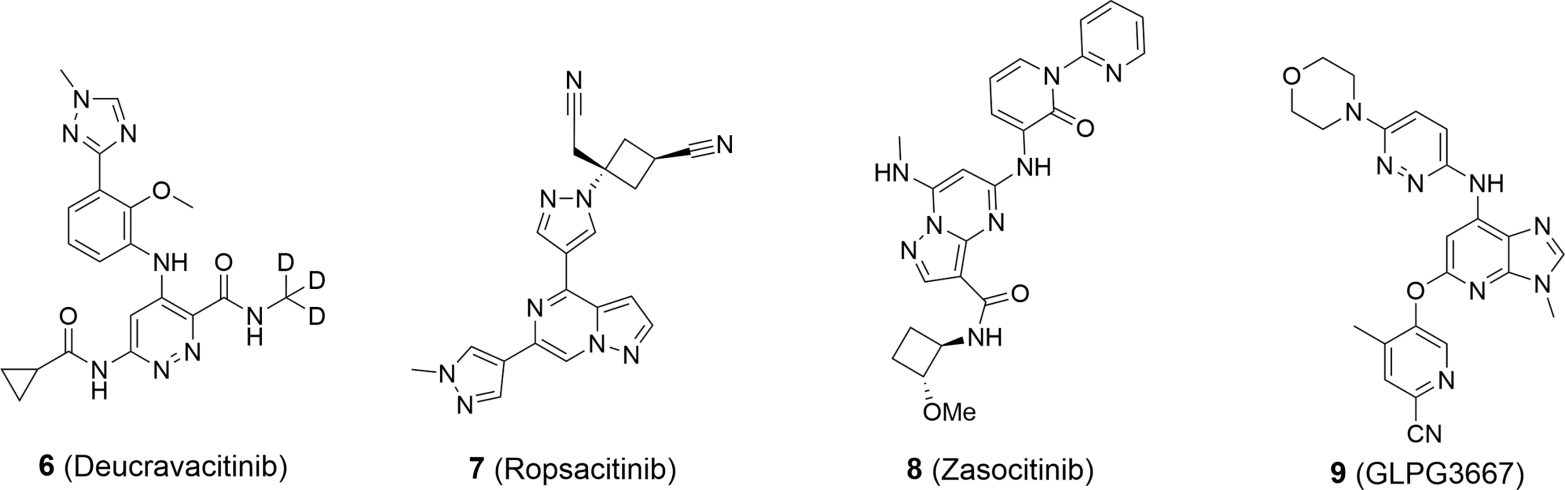
Investigational tyrosine kinase 2 inhibitors.^31,35,37^

Data from clinical trials of two small-molecule TYK2 inhibitors (Figure 2) have recently been published. Deucravacitinib **6** is an allosteric inhibitor that selectively blocks TYK2 kinase activity by stabilizing its pseudokinase domain.^31^ Efficacy of deucravacitinib has been demonstrated in patients with psoriasis,^32^ psoriatic arthritis,^33^ and SLE,^34^ with limited adverse effects, highlighting the potential benefits of this class for the treatment of inflammatory diseases. Ropsacitinib **7** (PF-06826647)^35^ is an adenosine triphosphate (ATP) mimic TYK2-selective inhibitor that displayed efficacy in a phase 2b psoriasis trial.^36^ More recently, new TYK2 inhibitors have been described or announced, notably, **8** (zasocitinib)—an allosteric TYK2-selective inhibitor—which was described as effective in a small phase 1b study in psoriasis patients^37^ and in a phase 2b study,^38^ and VTX958,^39^ which is also an allosteric inhibitor that just completed a phase 1 study in healthy volunteers.^39^

In a manuscript currently in development, we described a 1*H*-imidazo[4,5-*c*]pyridine series capable of dual JAK1/TYK2 inhibition and selective against JAK2 and JAK3.^40^ In the present article, we describe our efforts to progress to a TYK2-selective inhibitor with reduced potency for JAK1 and suitable for clinical progression. These efforts culminated in the discovery of **9** (GLPG3667), a selective TYK2 inhibitor targeting the catalytic domain, which is currently in clinical development for the treatment of inflammatory diseases (Figure 2).

## Results and Discussion

### Scaffold-Hopping Exercise to Improve JAK1/TYK2 Selectivity

**Figure 3:**
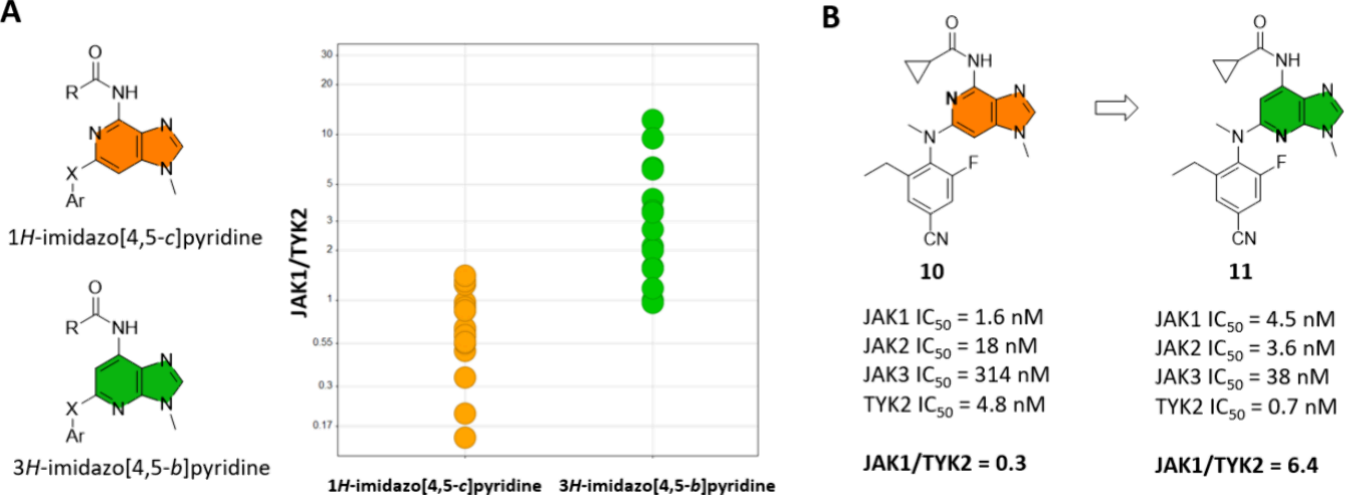
Selectivity comparison between the (A) 1*H*-imidazo[4,5*-c*]pyridine and the 3*H*-imidazo[4,5*-b*]pyridine scaffolds and (B) matched pairs **10** and **11**. IC_50_ values obtained from fluorescence-based biochemical assays using the catalytic domains of the four JAK members. Selectivity determined as the ratio of the IC_50_ values for JAK1/TYK2.

To initiate this project, a 3*H*-imidazo[4,5-*b*]pyridine series was designed and synthesized as part of a scaffold-hopping exercise. A comparison of this scaffold with the 1*H*-imidazo[4,5-*c*]pyridine series, both bearing a carbonyl-amino substituent at the top, clearly indicated that the 3*H*-imidazo[4,5-*b*]pyridine scaffold possessed a more favorable TYK2 selectivity profile (Figure 3A). Comparison of matched pairs **10**(40) and **11**, both possessing a cyclopropane carboxamide at the top (positions C4 and C7, respectively), showed a 21-fold improvement in JAK1/TYK2 selectivity (Figure 3B).

**Figure 4:**
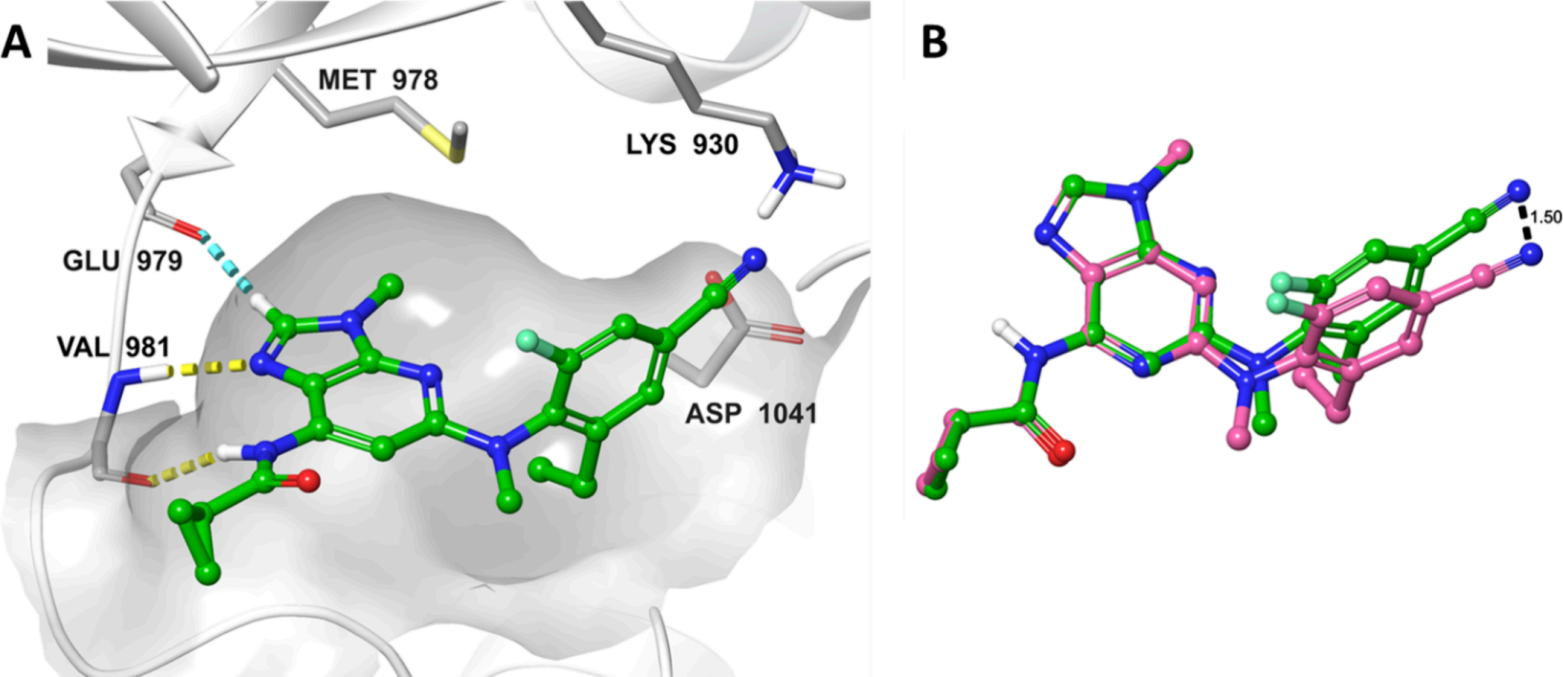
(A) Compound **11** docked in TYK2 structure (Protein Data Bank code 3LXN). Compound **11** is displayed in green ball-and-sticks. Only key amino acids of TYK2 are shown (gray sticks). Hydrogen bonds are highlighted with yellow dotted lines, and the aromatic H-bond is materialized with a dotted blue line. (B) Quantum mechanics minimized structures of **10** (pink) and **11** (green), overlaid on the imidazole ring. A distance of 1.5 Å could be measured between the nitrogen atoms of the cyano group.

Docking of **10** and **11** in JAK1 and TYK2 structures showed very similar binding modes in both proteins (Figure 4A). In the kinase hinge region, the imidazole nitrogen established a hydrogen bond with the backbone NH of Val981 (TYK2 numbering), while the hydrogen borne by the carbon between both nitrogen atoms formed a weak C–H–O interaction with the backbone carbonyl of Glu979. The amide NH was also involved in a hydrogen bond to the carbonyl oxygen of Val981 in the hinge region. The nitrile group pointed toward the Gly-rich loop, but without any clear interaction. The ethyl substituent filled a hydrophobic pocket (lined by Leu1030, Gly1040, and the backbone of Asn1028; residues not shown for clarity). Quantum mechanics minimization of **10** and **11** docking poses and overlay of the resulting structures showed that the different position of the nitrogen atom in the bicyclic core induced a shift in the location of the aryl and cyano groups that point toward the Gly-rich loop (Figure 4B). Engagement of the Gly-rich loop has often been cited as a way of modulating the selectivity profile within the JAK family,^35^ so this difference may explain the increased selectivity of 3*H*-imidazo[4,5-*b*]pyridine compounds.

### Initial Structure–Activity Relationship Exploration at the C7 Position

The initial structure–activity relationship (SAR) exploration focused on the C7 position of the 3*H*-imidazo[4,5-*b*]pyridine scaffold (Table 1). All compounds were potent TYK2 inhibitors (half maximal inhibitory concentration [IC_50_] < 1 nM). Compared with **11**, the urea **12** maintained good selectivity for TYK2 versus JAK1 and showed improved lipophilic efficiency (LipE), an important parameter in lead optimization;^41^ however, there was a major 37-fold selectivity with the pyrimidin-4-amine **13**. All compounds displayed very good selectivity (>30×) for TYK2 against JAK3, but low selectivity against JAK2 (<5×). A lower biochemical selectivity against JAK2, though undesirable, was less concerning because JAK2 inhibitors have been shown to undergo larger biochemical to cellular potency shifts than JAK1 and TYK2 inhibitors.^42^ Different hypotheses were developed to explain this disconnect, i.e., the low ATP K_m_ of JAK2 compared with other JAK family members^43^ or a difference in contribution to signaling between the JAK family members.^44^ All compounds had a high *in vitro* unbound intrinsic clearance^45,46^ of >100 L/h/kg in mouse liver microsomes.

**Table 1:**
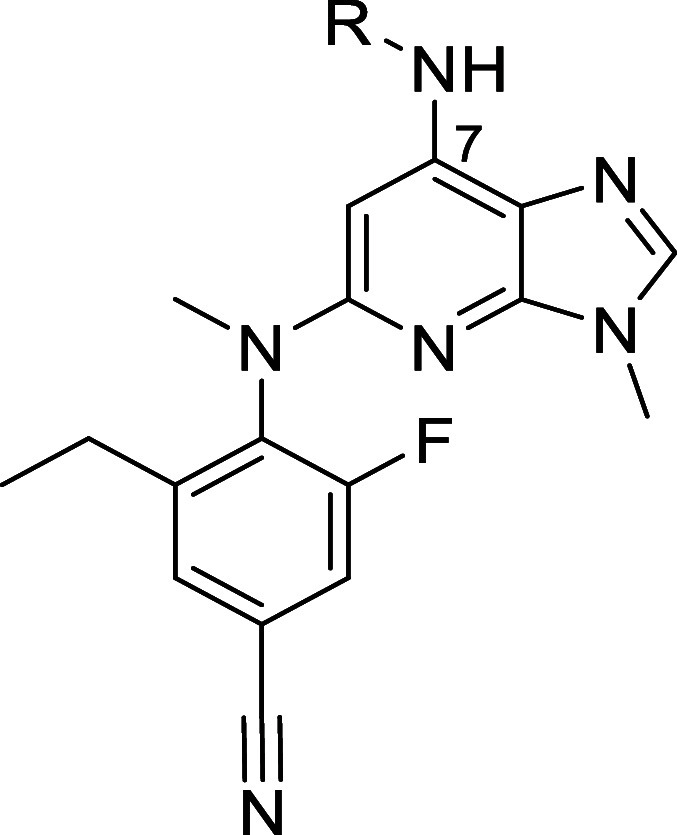

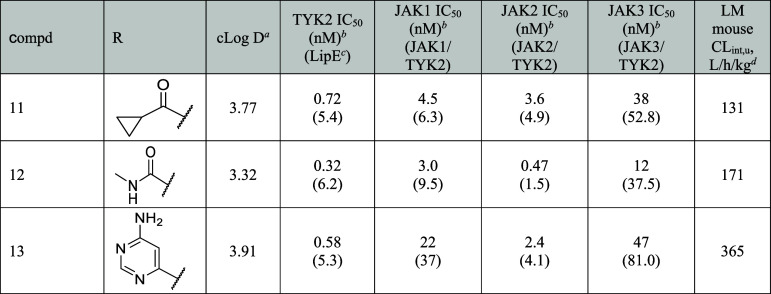
SAR Generated by Variations at the C7 Position of the 3*H*-imidazo[4,5*-b*]pyridine Series

a Calculated by Simulation Plus;^47^

b geometric mean of at least two experiments;

c LipE = pIC_50_ – cLog D;

d intrinsic unbound clearance in mouse liver microsomes (LM).

**Figure 5:**
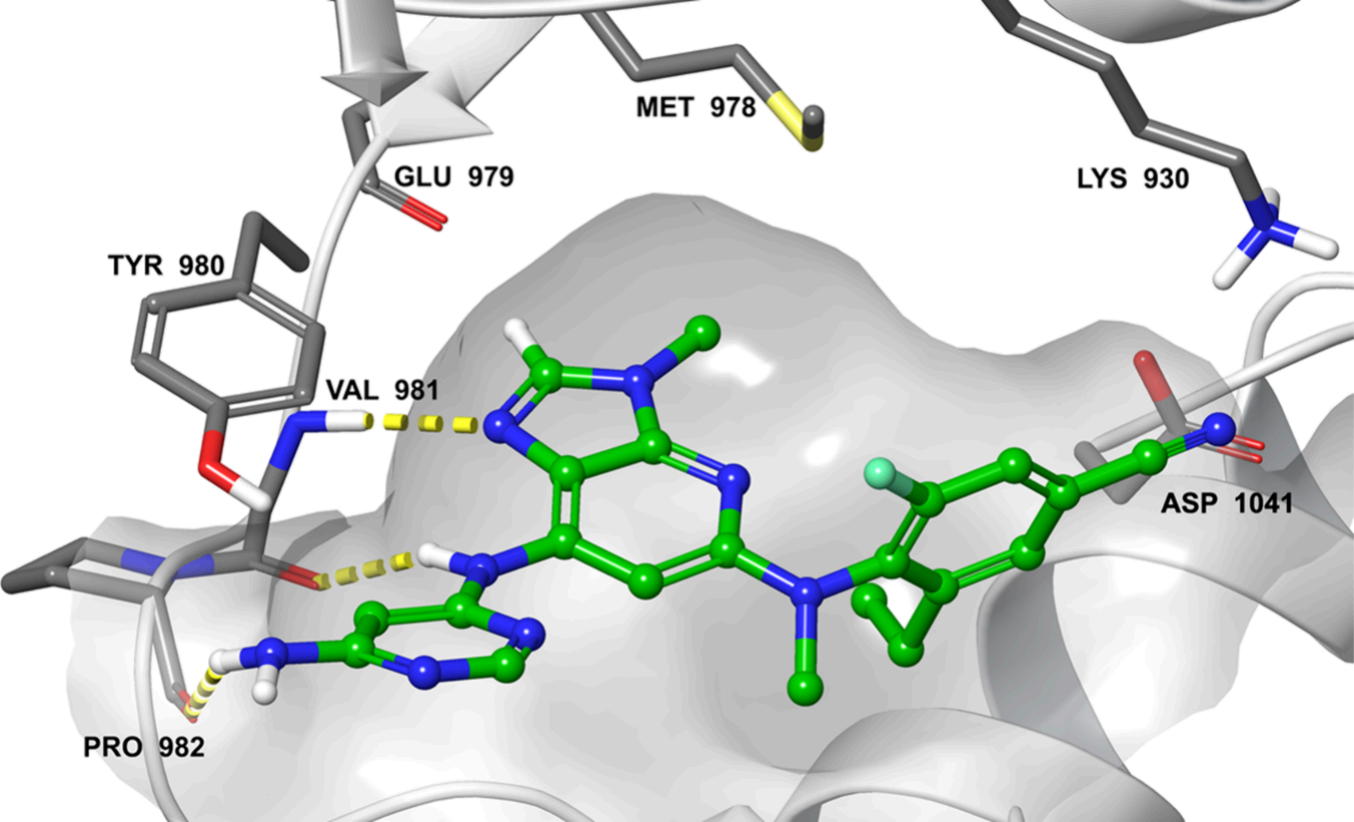
Compound **13** docked in TYK2 structure (Protein Data Bank code 3LXN). Compound **13** is displayed in green ball-and-sticks. Only key amino acids of TYK2 are shown (gray sticks). Hydrogen bonds are highlighted with yellow dotted lines.

Docking of **13** in TYK2 and JAK1 structures suggested that the aminopyrimidine group points toward the solvent along the hinge region, with the amino group establishing a hydrogen bond with the backbone oxygen of Pro982 (Figure 5). These interactions are common to both TYK2 and JAK1 and do not account for the observed TYK2 selectivity. However, molecular dynamics simulations of this compound in both proteins showed that in TYK2, the amino group can also interact with the oxygen atom of Tyr980 (Phe958 in JAK1). This hydrogen bond was not stable but appeared frequently during the simulation, suggesting a weak interaction in TYK2 but not in JAK1.

### SAR Exploration at the C5 Position

The next focus was the C5 position of the 3*H*-imidazo[4,5*-b*]pyridine scaffold, retaining the pyrimidin-4-amine at the C7 position in light of its improved TYK2 selectivity profile (Table 2). A key strategy was to explore the use of alkyl tails in this position to reduce the number of aromatic rings to increase saturation, reported as an approach to improving clinical success.^48^ Compounds **14** and **15** led to a reduction in potency for TYK2 compared with **13**, and insufficient improvements in *in vitro* clearance despite lower lipophilicity. TYK2 potency was regained with **16** but JAK1/TYK2 selectivity was reduced approximately 3-fold compared with **15** and the metabolic stability in mouse microsomes did not improve, likely owing to the relatively high lipophilicity. The replacement of the methylamino linker with an NH linker in **17** provided a major improvement in terms of *in vitro* clearance, while retaining potency and selectivity toward TYK2; however, *in vitro* clearance in mouse microsomes was still above 10 L/h/kg. The O-linker in **18** also provided an improvement in *in vitro* clearance, though not to the same extent as the NH linker. Efforts were also made to improve the compounds with an aromatic group at the C5 position. The introduction of a pyridine moiety in **19** led to a considerable reduction in *in vitro* clearance, while conserving potency and selectivity. Replacing the ethyl group in the aromatic tail of **19** with a methyl group in **20** led to approximately a 2-fold reduction in *in vitro* clearance and an improvement in JAK2/TYK2 selectivity. While the *in vitro* clearance was still rather high, these improvements clearly demonstrated that modification of the aromatic tail had potential to improve metabolic stability. Utilizing the lessons learned from matched pairs **17** and **18**, the linker connecting the aromatic tail to the core was modified in **21** and **22** to further improve *in vitro* clearance; the O-linked **22** had an *in vitro* clearance of below 4.1 L/h/kg. Despite some loss of JAK1/TYK2 selectivity, the improved metabolic stability of **22** was considered to provide a balanced profile. The less potent O-linked **23** also showed good metabolic stability, with an *in vitro* clearance value in mouse liver microsomes <10 L/h/kg.

**Table 2:**
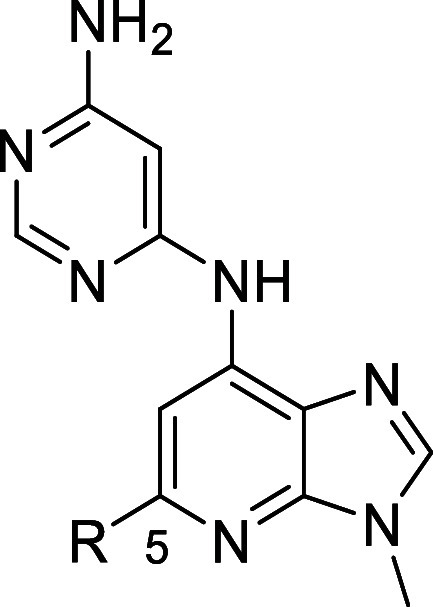

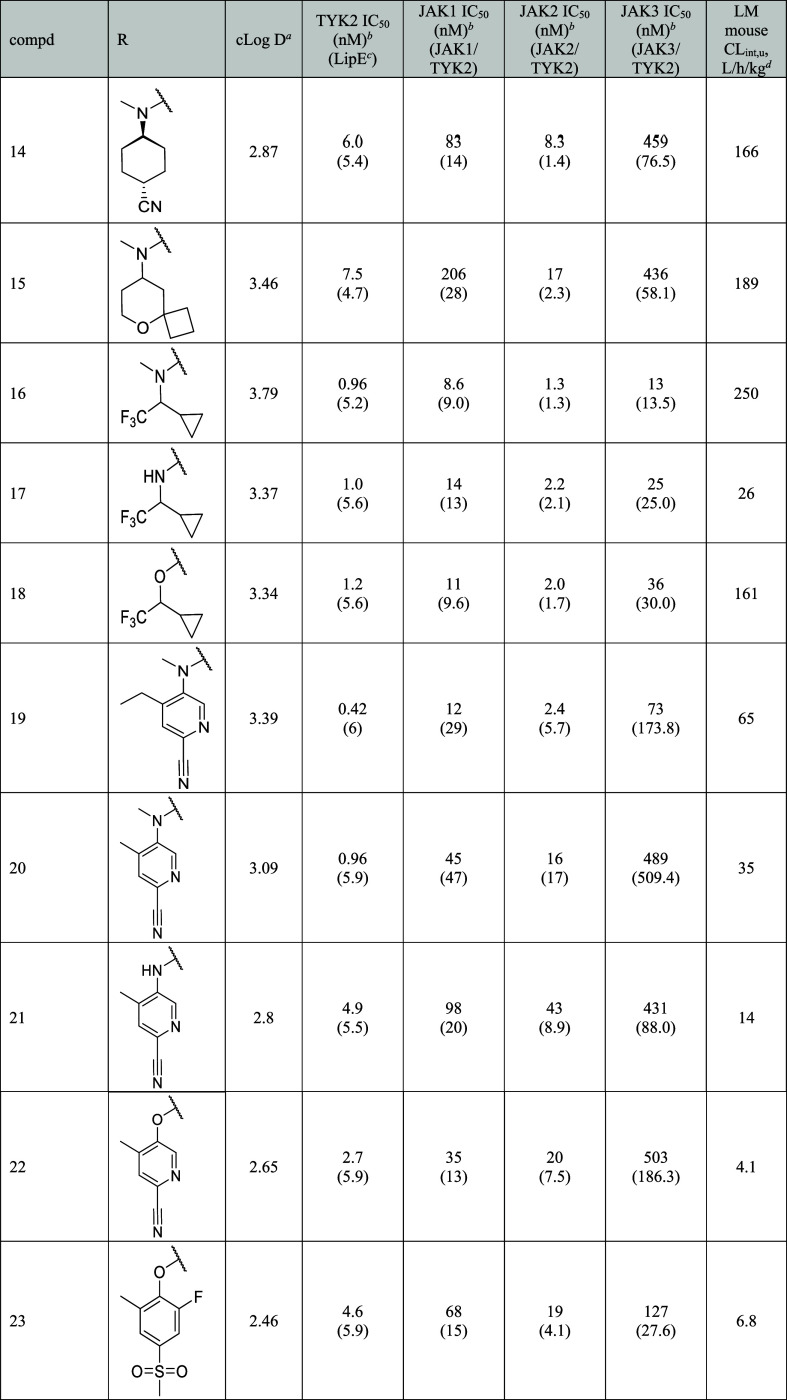
SAR Generated by Variations at the C5 Position

a Calculated by Simulation Plus;^47^

b geometric mean of at least two experiments;

c LipE = pIC_50_ – cLog D;

d intrinsic unbound clearance in mouse liver microsomes (LM).

The compounds described in Table 2 indicated that aromatic groups at the C5 position conferred superior selectivity and, in some cases, potency compared with their alkyl counterparts. Analysis of a set of compounds bearing the aminopyrimidine group at the C7 position reinforced these conclusions (Figure S1, Supporting Information). The compounds bearing alkyl substituents had lower potency and selectivity compared with the compounds bearing aromatic tails.

**Figure 6:**
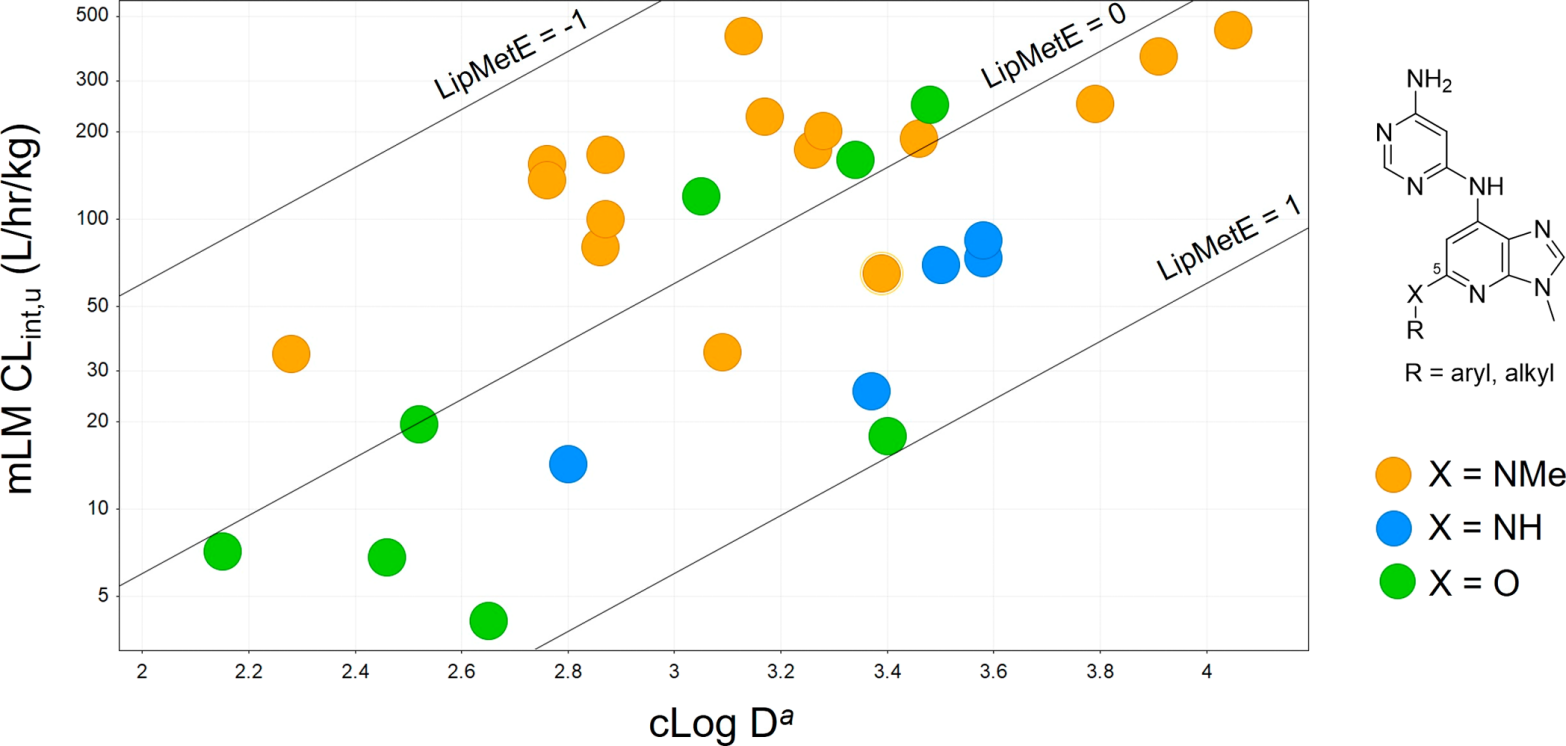
Relationship between calculated distribution coefficient (cLog D) and unbound intrinsic clearance (CL_int,u_) from incubations with mouse liver microsomes (mLM). ^*a*^Calculated by Simulation Plus.^47^ Solid lines show regions with equal lipophilic metabolic efficiency (LipMetE) (LipMetE = cLog D – log10[CL_int,u_], where CL_int,u_ is expressed in mL/min/kg).

The improved *in vitro* clearance of compounds **17**, **18**, **21**, and **22** in mouse liver microsomes appears to have stemmed from modifications of the linker. These modifications were further analyzed by considering the lipophilicity. The inherent stability of a set of compounds bearing an aminopyrimidine group at the C7 position was assessed, using the lipophilic metabolic efficiency metric (LipMetE)^49^ to remove the contribution of lipophilicity (Figure 6). This analysis indicated an NH- or an O-linker was more stable than an NMe-linker. Owing to the additional hydrogen bonding donor of the NH linker, a property which should be carefully considered for compound progression,^50^ the O-linked **22** was selected for further optimization.

### Advanced SAR Exploration at the C7 Position

Two subseries of compounds with carboxamide or aniline groups connected to the C7 position protruding toward the solvent were designed (Table 3) to improve on the thermodynamic solubility of **22**, which was low (<100 μg/mL) in all media.

**Table 3:**
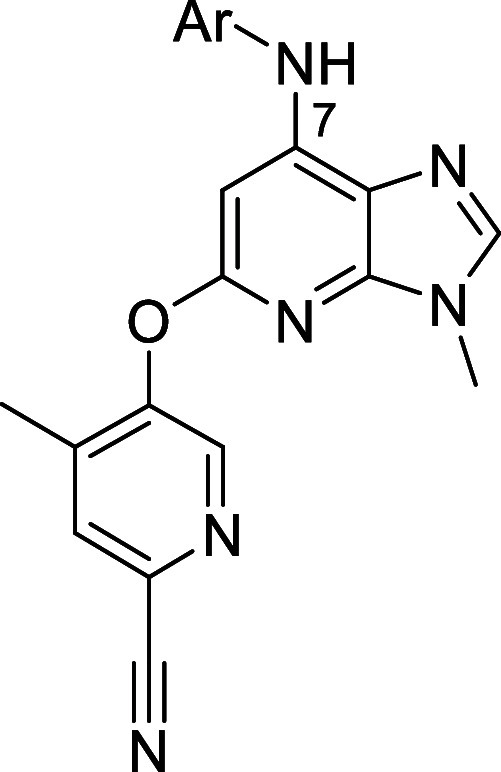

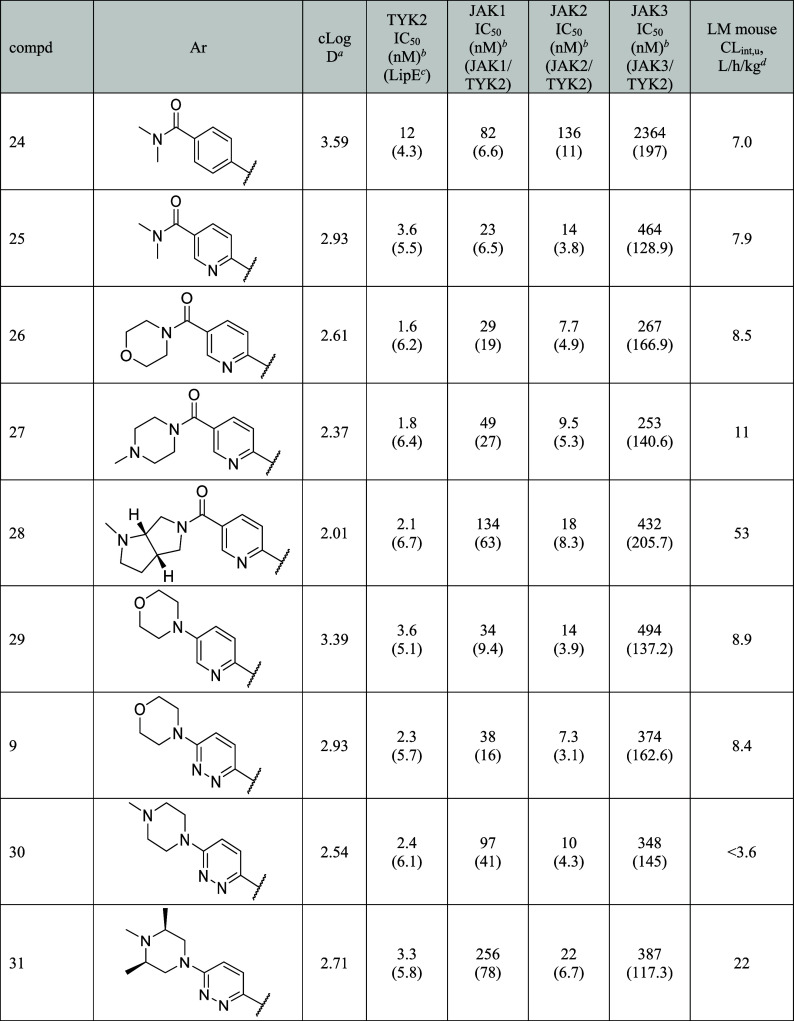
SAR Generated by Variations at the C7 Position

a Calculated by Simulation Plus;^47^

b geometric mean of at least two experiments;

c LipE = pIC_50_ – cLog D;

d intrinsic unbound clearance in mouse liver microsomes (LM).

In the carboxamide subseries, potency and LipE lost with **24** were regained by replacing the benzene ring with pyridine in **25**; however, both compounds had low JAK1/TYK2 selectivity (<10×). The morpholine in **26** and the *N*-methylpiperazine in **27** all increased potency and JAK1/TYK2 selectivity by at least 3-fold compared with **25**. Compounds **24**–**27** all had good *in vitro* metabolic stability in mouse liver microsomes around 10 mL/h/kg. The chirally pure **28** showed exquisite JAK1/TYK2 selectivity (63×), but metabolic stability was compromised. In the aniline subseries, **29** displayed good potency and JAK1/TYK2 selectivity. The replacement of the pyridine with a pyridazine in **9** improved selectivity 2-fold, with a slight increase in potency and LipE. As with the carboxamides, the introduction of basic groups was generally associated with an increase in selectivity. **30** and **31** showed (41×) and (78×) JAK1/TYK2 selectivity, respectively, but metabolic stability was eroded with **31**.

Most of the compounds shown in Table 3 had an attractive profile with good potency, LipE, selectivity, and metabolic stability (in mouse liver microsomes). Some compounds were therefore subjected to more advanced absorption, distribution, metabolism, excretion, and toxicity profiling (Table 4). In general, permeability was moderate to good but efflux ratios were high, indicating that the compounds could be P-glycoprotein substrates.^51^ The carboxamide **27** with basic bearing group had a particularly high efflux ratio. Neutral carboxamide **26** had better solubility than **22**, with moderate solubility in acidic media. Basic groups in **27** and **30** further improved solubility in acidic media, as expected. **9** showed a considerable 10-fold improvement in solubility in fasted state simulated gastric fluid compared with **22**. The compounds had acceptable *in vitro* metabolic stability in mouse and human liver microsomes. Human ether-à-go-go-related gene (hERG) inhibition was low for all compounds except **30**, which bears a basic piperazine (68% inhibition at 10 μM).

**Table 4:**
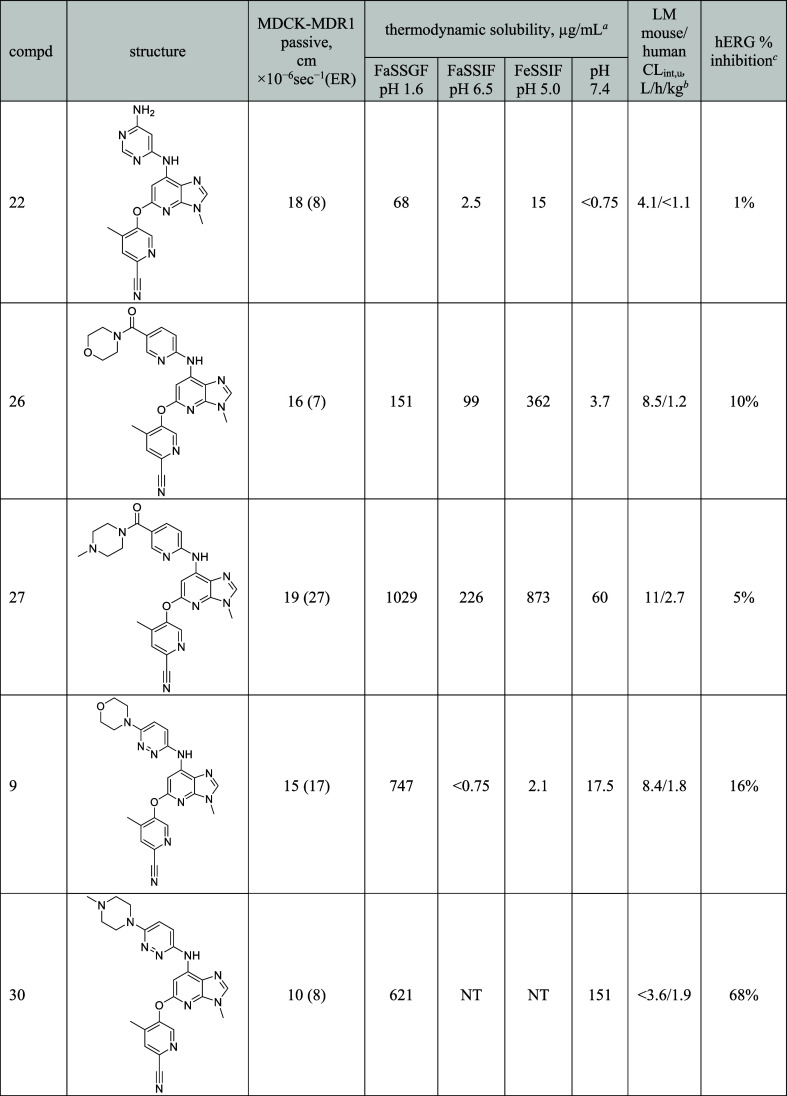
Selected Absorption, Distribution, Metabolism, Excretion, and Toxicity (ADMET) Properties of Advanced Compounds

a Solid state form not determined;

b intrinsic unbound clearance in mouse/human liver microsomes (LM);

c percentage of inhibition at 10 μM compound, single dose; assay conditions: automated patch clamp. NT, not tested.

### *In Vivo* Pharmacokinetic Profiles of Advanced Compounds

Compounds **26**, **27**, and **9** were progressed to mouse pharmacokinetic (PK) experiments (Table 5). Compounds **26** and **27** had moderate *in vivo* clearance. The volume of distribution was moderate for all compounds, and the basic group of **27** did not lead to a high volume of distribution. The combination of these factors led to half-lives of less than 1 h for all compounds. Bioavailability of **26** and **27** was low to moderate. **9** displayed lower clearance, with a volume of distribution of ∼2 L/kg, resulting in a good half-life (1.6 h) and improved bioavailability.

**Table 5:** Mouse Pharmacokinetic Properties of Compounds **26**, **27**, and **9**

	mouse PK^a^ (1 mg/kg (iv)^b^			mouse PK^a^ (5 mg/kg po)^c^				
compd	CL (CL_unbound_) (L/h/kg)	V_ss_ (L/kg)	half-life effective (h)	*F* (%)	AUC_0–inf_ (ng·h/mL)	*C*_max_ (ng/mL)	*T*_max_ (h)	mouse PPB^d^
**26**	1.5 (9.0)	1.2	0.55	29	1130	619	0.5	83.9
**27**	2.6 (22)	2.5	0.67	31	589	500	0.25	87.8
**9**	0.82 (9.9)	1.9	1.6	45	2744	1570	1.5	91.7

a *n* = 6 male CD1 mice. All values were obtained from plasma;

b vehicle was polyethylene glycol 200/water for injection (60/40; v/v);

c vehicle was Solutol HS15/methyl cellulose 0.5% (2/98; v/v);

d plasma protein binding.

Compounds **26**, **27**, and **9** were tested *in vitro* in cellular and whole blood assays to assess potency and selectivity against the JAK family members in more relevant systems (Table 6). In the cellular assays performed on fresh human peripheral blood mononuclear cells (PBMCs), the three compounds showed dose-dependent inhibition of IFNα-induced STAT1 phosphorylation (JAK1/TYK2-dependent), IL-2-induced STAT5 phosphorylation (JAK1/JAK3-dependent) and granulocyte macrophage colony-stimulating factor (GM-CSF)-induced STAT5 phosphorylation (JAK2-dependent) and displayed more than 10-fold selectivity when comparing the assays on TYK2-dependent pathways (e.g., IFNα-driven assays) with the assays on JAK1/JAK3- and JAK2-dependent assays. In human whole blood assays, the potency of the three compounds was highest in the TYK2-dependent IFNα-induced STAT1 phosphorylation assay compared with the IL-6-induced STAT1 phosphorylation (JAK1-dependent), GM-CSF-induced STAT5 phosphorylation, and IL-2-induced STAT5 phosphorylation assays. Comparison with data obtained with a JAK1-selective inhibitor (filgotinib) and the TYK2-selective inhibitor deucravacitinib further validate the TYK2 selectivity of compound **9**. Overall, these results indicate that compounds **26**, **27**, and **9** are selective TYK2 inhibitors in human PBMC and whole blood assays, further reinforcing the data obtained with the biochemical assays. Of note, potency of all compounds decreased in whole blood assays compared with PBMC. This is likely due to the absence of serum in the PBMC assays that delivers free fraction activity, while in whole blood, part of the molecules are trapped by plasma protein as frequently documented.^52,53^

**Table 6:** Potency of **9**, **26**, **27**, Deucravacitinib, and Filgotinib in Cellular and Whole Blood Assays

	human peripheral blood mononuclear cell assays^a^			human whole blood assays^b^			
assay	IFNα/pSTAT1	IL-2/pSTAT5	GM-CSF/pSTAT5	IFNα/pSTAT1	IL-6/pSTAT1	IL-2/pSTAT5	GM-CSF/pSTAT5
kinase dependency	JAK1/TYK2	JAK1/JAK3	JAK2	JAK1/TYK2	JAK1	JAK1/JAK3	JAK2
**9**	70 nM	1113 nM	1022 nM	623 nM	7974 nM	17,512 nM	12,784 nM
	(*n* = 6)	(*n* = 6)	(*n* = 6)	(*n* = 13)	(*n* = 6)	(*n* = 3)	(*n* = 6)
**26**	65 nM	882 nM	1794 nM	478 nM	3466 nM	NT	NT
	(*n* = 1)	(*n* = 1)	(*n* = 1)	(*n* = 5)	(*n* = 3)		
**27**	61 nM	595 nM	917 nM	138 nM	2518 nM	NT	9274 nM
	(*n* = 13)	(*n* = 13)	(*n* = 13)	(*n* = 9)	(*n* = 3)		(*n* = 3)
deucravacitinib	NT	NT	NT	34 nM	660 nM	8500 nM	26,900 nM
				(*n* = 3)	(*n* = 2)	(*n* = 3)	(*n* = 3)
filgotinib	NT	NT	NT	1127 nM	629 nM	1127 nM	17,453 nM
				(*n* = 6)	(*n* = 7)	(*n* = 5)	(*n* = 7)

a Experiments performed on peripheral blood mononuclear cells triggered with IFNα, IL-2, or GM-CSF.

b Experiments performed in human whole blood triggered with IFNα, IL-6, IL-2, and GM-CSF. In all cases, *n* refers to the number of experiments performed to determine IC_50_ values. The results are the geometrical mean of the different tests. NT, not tested.

These three compounds were subsequently tested in an *in vitro* micronucleus test in the human lymphoblastoid TK6 cell line in the presence or absence of S9 fraction. Compounds **26** and **27** induced structural DNA damage, in contrast to compound **9** which did not and was selected for further profiling.

### *In Vitro* Profiling of **9**

Due to its good global profile, **9** was further tested for general kinase selectivity against a panel of 365 kinases (performed at Eurofins Discovery, Cerep, France), at a concentration of 1 μM (Figure S2, Supporting Information). The compound only hit 21 non-JAK kinases with at least 50% inhibition. Biochemical IC_50_ was <100 nM for three kinases (AURKB, FLT3, and FLT4), and cellular IC_50_ values (generated at Reaction Biology, Freiburg, Germany) were in the micromolar range for AURKB and FLT3 (FLT4 was not tested) (Table S1, Supporting Information). No off-target activity was reported in the diversity panel (performed at Eurofins Discovery, Cerep, France) at 10 μM for any of the 97 binding, enzyme, and uptake assays.

### Additional Profiling of **9**

Further *in vitro* profiling is listed in Table 7. Compound **9** was highly stable *in vitro* and in mouse, rat, dog, and human liver microsomes and hepatocytes. It was highly permeable in Caco-2 cells, with a low efflux ratio. The IC_50_ for cytochrome P450 (CYP) inhibition was >33 μM for each of the CYP isoenzymes tested and there was no evidence of time-dependent inhibition of CYP3A4. Additionally, there was no meaningful mRNA induction of CYP3A4 in cryopreserved human hepatocytes (2.2% vs rifampicin at 10 μM). The mutagenic potential of **9** was also investigated with a bacterial reverse mutation test (Ames test) using three different bacterial strains, which was negative. As reported above, compound **9** did not induce structural DNA damage in an *in vitro* micronucleus test in the human lymphoblastoid TK6 cell line in the presence or absence of S9 fraction. **9** was highly stable in plasma of mouse, rat, dog, and human, and chemically stable in solutions of pH 1.2, 5.0, 7.4, and 9.0 for up to 24 h, with >80% of compound remaining at 2 and 24 h (Table S2, Supporting Information). The profiling of **9** did not reveal liabilities and the compound passed the preclinical toxicity studies to advance to clinical studies.

**Table 7:** *In Vitro* Profile of **9**

Assays	Analogues **9**
Tyrosine kinase 2 IC_50_ (nM)	2.3
Cellular assay IFNα/pSTAT1 IC_50_ (nM)	70
Whole blood IFNα/pSTAT1 IC_50_ (nM)	623
Thermodynamic solubility (μg/mL)	pH 7.4: 6.05–17.5
	FeSSIF: 3.02–2.09
	FaSSIF: <0.75
	FaSSGF: 289–747
Microsome stability CL_int,u_ (L/h/kg)	Mouse: 8.43
	Rat: 2.75
	Dog: 2.13
	Human: 1.78
Hepatocyte stability CL_int,u_ (L/h/kg)	Mouse: 4.75
	Rat: 0.90
	Dog: <0.90
	Human: <0.47
Intestinal permeability Caco-2 (pH 6.5) P_app_ A_2_B (×10^–6^ cm/s)/ER	23/1.33
CYP inhibition HLM IC_50_ (μM)	>33 μM for CYP1A2/CYP2C19/CYP2C9/CYP2D6/CYP3A4 (midazolam/testosterone)
CYP3A4 inhibition TDI midazolam/testosterone IC_50_ fold shift	No shift (all IC_50_ > 33 μM)
CYP3A4 induction (mRNA) fold increase/% of rifampicin response	1.18/2.2%
Plasma protein binding (%)	Mouse: 91.8
	Rat: 92.5
	Dog: 84.7
	Human: 92.6
Ames	Negative
μNucleus	Negative
Plasma stability	Stable in mouse, rat, dog, and human
Chemical stability	Stable at pH 1.2, 5.0, 7.4, and 9.0

**Figure 7:**
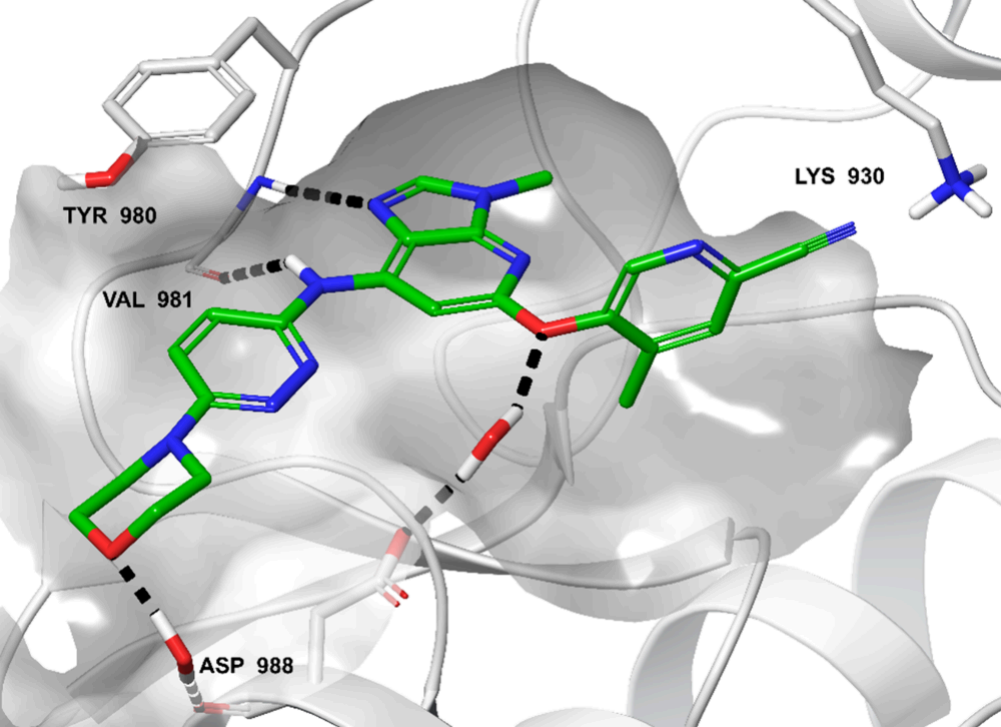
Representative frame of a molecular dynamics simulation of compound **9** docked in TYK2 structure (Protein Data Bank code 3LXN). Compound **9** is displayed in green sticks. Only key amino acids of TYK2 are shown (gray sticks). Bridging water molecules that were stable during the simulation are displayed. Hydrogen bonds are highlighted with black dotted lines.

Additionally, compound **9** was docked and its binding mode was compared to that of the earlier compound **13** in the SAR campaign (Figure 7). The resulting model suggests that in the kinase hinge region, the imidazole nitrogen establishes a hydrogen bond with the backbone NH of Val981 (human TYK2 numbering), as for compound **13.** The nitrile group points toward the Gly-rich loop, but without any clear interaction. The methyl substituent (ethyl in compound **13**) fills a hydrophobic pocket (lined by Leu1030, Gly1040, and the backbone of Asn1028; residues not shown for clarity). The NH linker could also be involved in a hydrogen bond to the carbonyl oxygen of Val981 in the hinge region (as is the amide NH in compound **13**). The morpholine moiety points toward the solvent-exposed region. Two water molecules might bridge the oxygen of the morpholine moiety to the backbone of Asp988, and its side chain to the ether linker of the ligand. These water-mediated interactions could not be present in compound **13**, due to the absence of acceptor atoms in the substituents.

### *In Vivo* Profiling of **9**

PK profiles were further explored in mouse, rat, and dog (Table 8). Compound **9** was characterized by low clearance, moderate volume of distribution, moderate half-life, rapid absorption following oral administration, and good oral bioavailability. In dogs, a modified formulation for oral administration was used due to low solubility and allowed to reach a bioavailability of 35%, which was considered suitable for progression.

**Table 8:** Mouse, Rat, and Dog Pharmacokinetic Properties of **9**

	mouse^a^	rat^a^	dog^b^
**Strain**	Male CD-1	Male Sprague–Dawley	Male Beagle
**Dosing**	1 mg/kg i.v.^c^	1 mg/kg i.v.^c^	0.25 mg/kg i.v.^c^
**CL (CL**_**un**_) **(L/h/kg)**	0.82 (9.91)	0.70 (7.18)	0.56 (3.63)
**V_ss_(L/kg)**	1.89	1.75	1.44
**Half-life (h) (i.v.) effective**	1.60	1.73	1.79
**Dosing**	5 mg/kg p.o.^d^	5 mg/kg p.o.^d^	30 mg/kg p.o.^e^
**Bioavailability (%)**	44.9	34.9	35.0
**AUC**_**0-inf**_**(L/h/kg)**	2744	2581	19,800
***C***_**max**_**(L/h)**	1570	1436	2720
***T***_**max**_**(h)**	1.5	0.25	3

a *n* = 6 per group, all values were obtained from plasma;

b *n* = 3 per group, all values were obtained from plasma;

c vehicle was polyethylene glycol 200/water for injection (60/40; v/v);

d vehicle was Solutol HS15/methylcellulose 0.5% (2/98; v/v);

e single gavage of a solid dispersion in corn oil. CL_u_, unbound clearance calculated using plasma protein binding data.

### Efficacy of **9** in a Murine Model of IL-23-Induced Psoriasis

**Figure 8:**
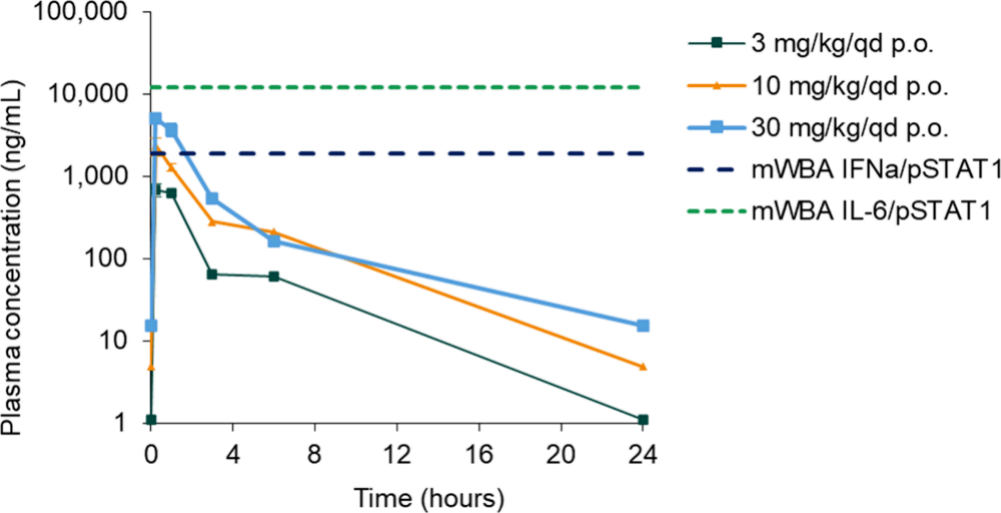
Pharmacokinetics after 4 days of treatment with **9**, dosed at 3, 10, and 30 mg/kg (q.d.). IC_50_ values of **9** for the two mouse pathways are deduced from mouse whole blood assays (see Supporting Information).

As shown by Gerstenberger et al. (2020),^35^ TYK2 inhibitors display limited selectivity for TYK2 in mouse compared with human due to decreased inhibition of TYK2 and increased inhibition of JAK1. For that reason, we only assayed our compound in a unique mouse model to verify that it displayed pharmacological effects when given orally to the animals. Compound **9** was evaluated at three doses (once daily [q.d.] oral administration at 3, 10, and 30 mg/kg) in the mouse model of IL-23-induced psoriasis.^54,55^ The exposure increased dose-proportionally between the dose range (Figure 8) allowing a limited coverage of the TYK2-dependent IFNα pathway and an absence of coverage of the JAK1-dependent IL-6 pathway for the two higher doses used.

**Figure 9:**
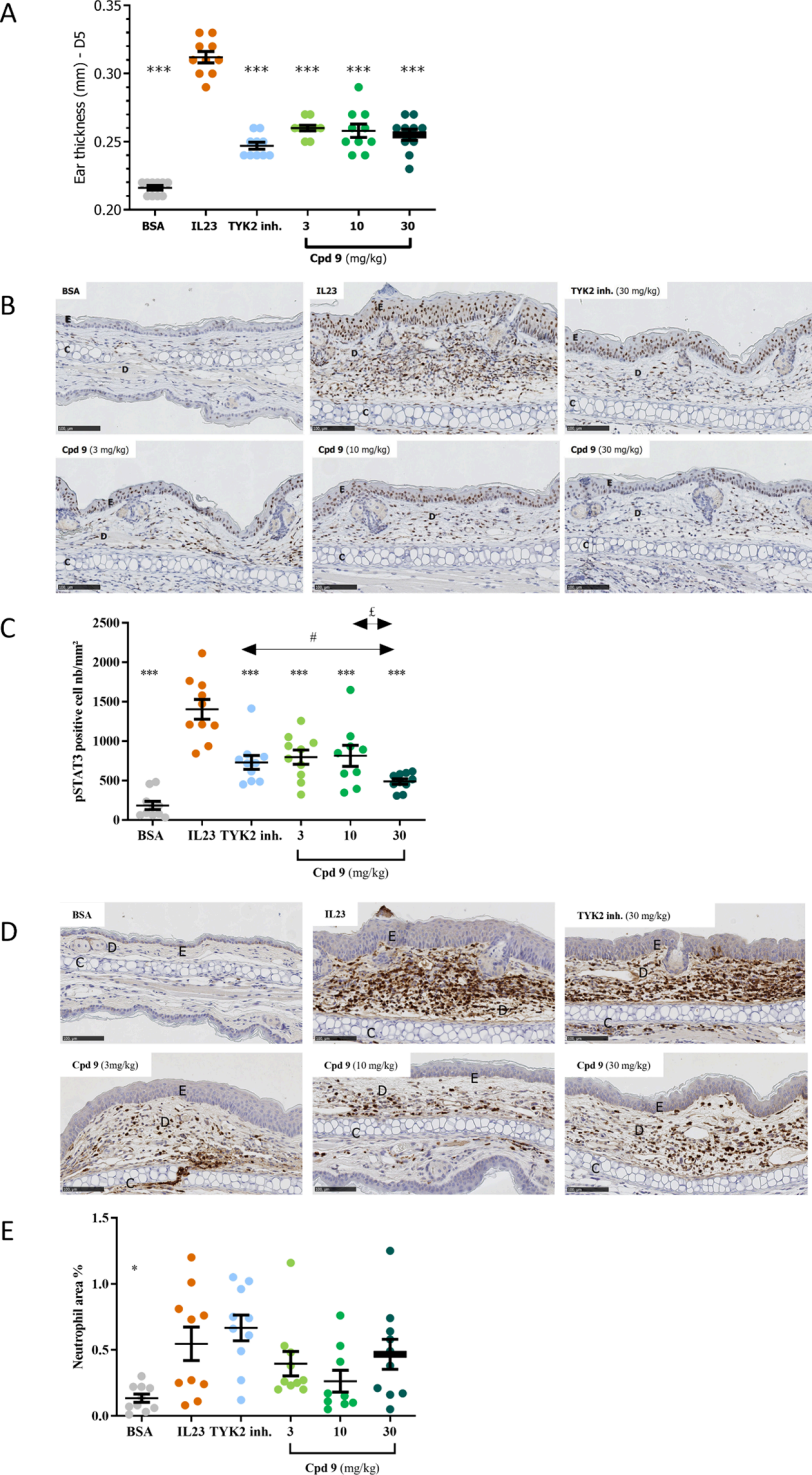
Effect of **9** dosed at 3, 10, and 30 mg/kg (q.d.) and TYK2 inhibitor used as positive control^56^ dosed at 30 mg/kg (q.d.) on IL-23-induced psoriasis-like inflammation in the ear skin of mice on (A) quantification of ear thickening and (B) pictures of inflammation and STAT3 phosphorylation in the ear tissue, (C) quantification of phosphorylated STAT3, (D) pictures of neutrophil (NIMP-R14-positive cells) infiltration in the ear tissue, and (E) quantification of neutrophil percentage area. Data shown are average ± standard error of the mean of *n* = 9–10 mice per group. Groups were compared using a one-way analysis of variance followed by Dunnett’s multiple comparisons test and, for part B, *t* test for two groups comparison: *p < 0.05, **p < 0.01, ***p < 0.001 versus vehicle group. ^#^*p* < 0.05 for TYK2 inh. group versus Cpd **9** 30 mg/kg group. ^£^*p* < 0.05 for Cpd **9** 10 mg/kg group versus Cpd **9** 30 mg/kg group. BSA, bovine serum albumin; C, cartilage; D, dermis; E, epidermis.

No dose-dependency was observed, and the effect of **9** was similar to the effect of a TYK2 inhibitor which was used as a positive control^56^ for preventing ear pinna thickening induced by intradermal injections of IL-23 (Figure 9A). While phosphorylated STAT3 (pSTAT3) is rarely expressed in healthy ear skin, IL-23 triggering led to a marked induction of STAT3 phosphorylation in keratinocytes and in some immune cells infiltrated in the dermis which is in line with the fact that STAT3 is the main target of IL-23. In the same experiment, compound **9** and the positive control TYK2 inhibitor prevented STAT3 phosphorylation in the epidermis and dermis at all tested doses, confirming target engagement and effect of the compound at the three doses used (Figure 9B, 9C). The absence of a dose–response relationship is likely due to the involvement of other pathways not impacted by TYK2 inhibition, and to the fact that mass balance studies in mice showed that compound **9** was highly concentrated in the skin (data not shown), suggesting that despite unfavorable PK, it displayed a strong effect in this psoriasis-like model. In addition, it is noteworthy that Leit et al. (2023)^52^ observed the same partial effects with the TYK2 inhibitor TAK-279, while this compound is more potent than compound **9** and displayed more favorable PK. Other compounds from the same series also gave a partial effect, strongly suggesting that in this model TYK2 inhibitors may not be able to fully counteract all the effects of IL-23.

We also looked at the presence of neutrophils in the ears of mice. Figure 9D shows that IL-23 injection induces a massive infiltration of neutrophils essentially in the dermis. Compound **9** was able to prevent this infiltration with no apparent a dose–response relationship (Figure 9E). Taken together, these data define a minimal effective dose of 3 mg/kg q.d. and define **9** as a selective TYK2 inhibitor that blocks the IL-23 pathway *in vivo*.^57^

### Human Dose Prediction and Clinical Efficacy of Compound **9**

Based on pharmacokinetics in mouse, rat, and dog, body weight allometry (rule of exponents with correction for maximum lifespan potency) predicted human blood clearance of 0.17 L/h/kg. Human volume of distribution at steady state (V_ss_) was estimated at 1.85 L/kg using the geometric mean of V_ss_ in mouse, rat, and dog. Human bioavailability was assumed to be 50% minimum. The human target concentration was based on coverage of human whole blood assay IFNα/pSTAT1 IC_50_ (623 nM) for 6 h in blood. Based on these assumptions, the human efficacious dose of compound **9** was predicted to be 100 mg, which is in line with efficacy signals seen with GLPG3667 at 150 mg q.d. in a phase 1b study of GLPG3667 in patients with moderate-to-severe psoriasis,^58^ in which compound **9** given orally at a dose of 150 mg q.d. displayed efficacy after only 4 weeks of treatment with PASI 75 comparable with what was observed with deucravacitinib without any safety alert.^59^

## Chemistry

**Scheme 1:**
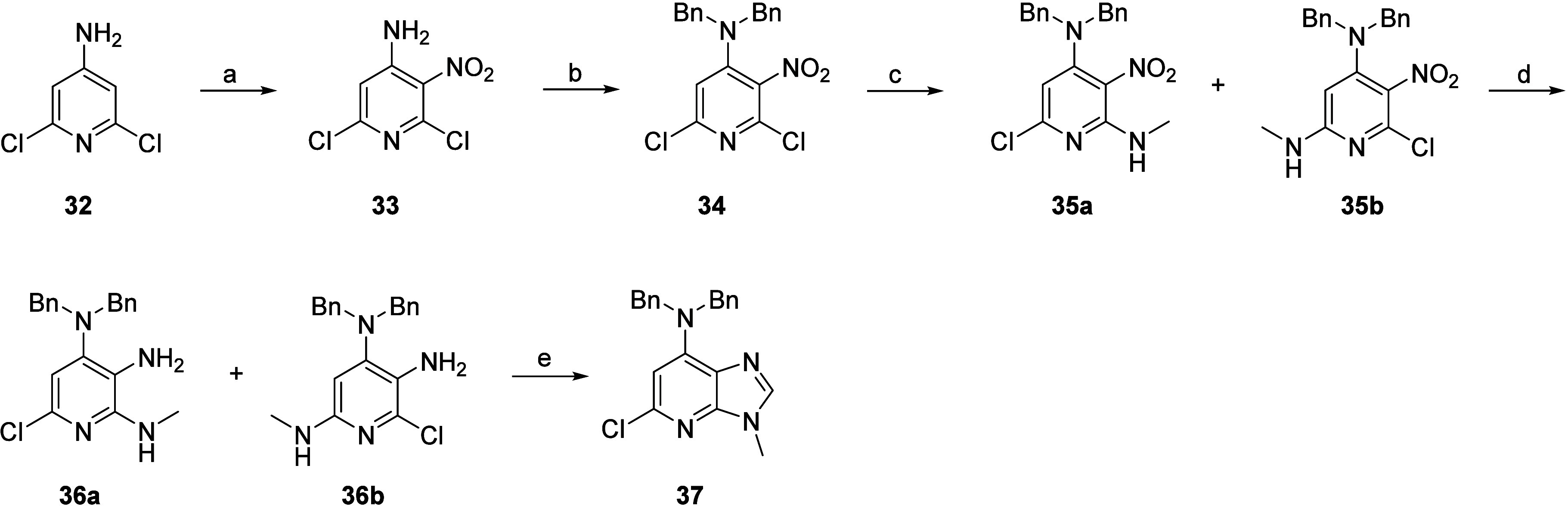
Synthesis of Core **37** Reagents and conditions: (a) HNO_3_, H_2_SO_4_, 10 °C for 30 min then 80 °C for 30 min, 86%; (b) benzyl bromide, K_2_CO_3_, DMF, 80 °C, 1 h, 84%; (c) 33% MeNH_2_ in EtOH, Et_3_N, THF, rt, 16 h; (d) Na_2_S_2_O_4_, NaHCO_3_, THF/H_2_0, rt, 1 h, crude; (e) triethyl orthoformate, MeCN, 80 °C, 16 h, 55%.

The preparation of the core **37**, required for synthesis of the compounds listed in Table 1, started with the treatment of commercially available 2,4-dichloro-3-aminopyridine **32** with a mixture of concentrated nitric acid and concentrated sulfuric acid to yield nitro intermediate **33**. Subsequent protection by treatment with two equivalents of benzyl bromide afforded intermediate **34**. The nucleophilic aromatic substitution with methylamine yielded a mixture of regioisomers **35a/35b**, which were reduced using sodium dithionite. The crude mixture of regioisomers **36a/36b** was cyclized by condensation with triethyl orthoformate. The scaffold **37** with the desired regioselectivity was obtained after silica flash chromatography (Scheme 1).

**Scheme 2:**
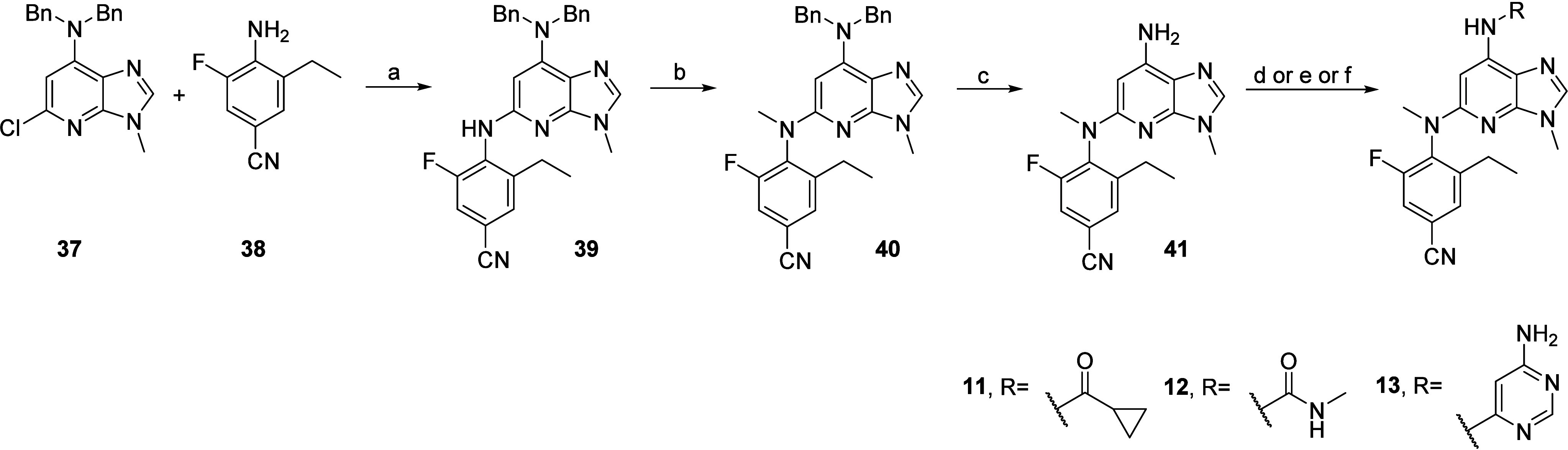
Synthesis of **11**, **12**, and **13** Reagents and conditions: (a) XantPhos, XantPhos Pd G3, Cs_2_CO_3_, 1,4-dioxane, 110 °C, 16 h; (b) MeI, NaH, THF, 0 °C then rt, 1 h, 74%; (c) TfOH, DCM, rt, 16 h; (d) for **11**: cyclopropanecarbonyl chloride, pyridine, DCM, rt, 2 h; (e) for **12**: CDT, pyridine, DCM, 50 °C, 1 h, then 2 M MeNH_2_ in THF, 1 h; (f) for **13**: 6-chloropyrimidin-4-amine, BrettPhos, BrettPhos Pd G3, Cs_2_CO_3_, 1,4-dioxane, 110 °C, 16 h.

Scheme 2 describes the synthesis of compounds **11**, **12**, and **13** listed in Table 1. Prerequisite trisubstituted aniline **38** was readily obtained in two steps from 4-amino-3-fluorobenzonitrile after iodination followed by Suzuki–Miyaura coupling with triethyl borane (Scheme S1, Supporting Information). Subsequent Buchwald–Hartwig cross-coupling between scaffold **37** and aniline **38**, performed using 2,2′-bis(diphenylphosphino)-1,1′-binaphthyl (BINAP), Pd(OAc)_2_ and Cs_2_CO_3_, yielded intermediate **39**. Methylation using methyl iodide led to **40**, which was followed by acid-promoted benzyl deprotection to yield the common intermediate **41**, allowing derivatization. **11** was obtained via acylation using cyclopropanecarbonyl chloride with pyridine in dichloromethane (DCM). **12** was obtained by activation of the aniline moiety with *N,N*′-carbonyl-di(1,2,4-triazole) followed by nucleophilic substitution of the second triazole with methyl amine. Finally, **13** was obtained via Buchwald–Hartwig cross-coupling with 6-chloropyrimidin-4-amine, using a combination of BrettPhos and precatalyst BrettPhos Pd G3 in the presence of Cs_2_CO_3_.

**Scheme 3:**
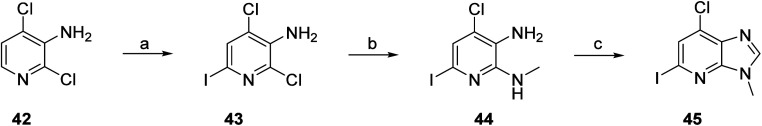
Synthesis of Core **45** Reagents and conditions: (a) *N*-iodosuccinimide, TFA, THF, rt, 16 h, 88%; (b) 2 M MeNH_2_ in THF, NMP, 180 °C, 48 h, 61%; (c) trimethyl orthoformate, formic acid, 60 °C, 1 h, 89%.

The preparation of the core **45**, required for synthesis of the compounds listed in Table 2, started with iodination of commercially available dichloro aniline pyridine **42** with *N*-iodo succinimide in tetrahydrofuran (THF) in the presence of trifluoroacetic acid (TFA). Halogenated pyridine **43** was subjected to aromatic nucleophilic substitution with 2 M methyl amine in THF to yield **44**, which was cyclized using trimethyl orthoformate and formic acid to yield core **45** (Scheme 3).

**Scheme 4:**
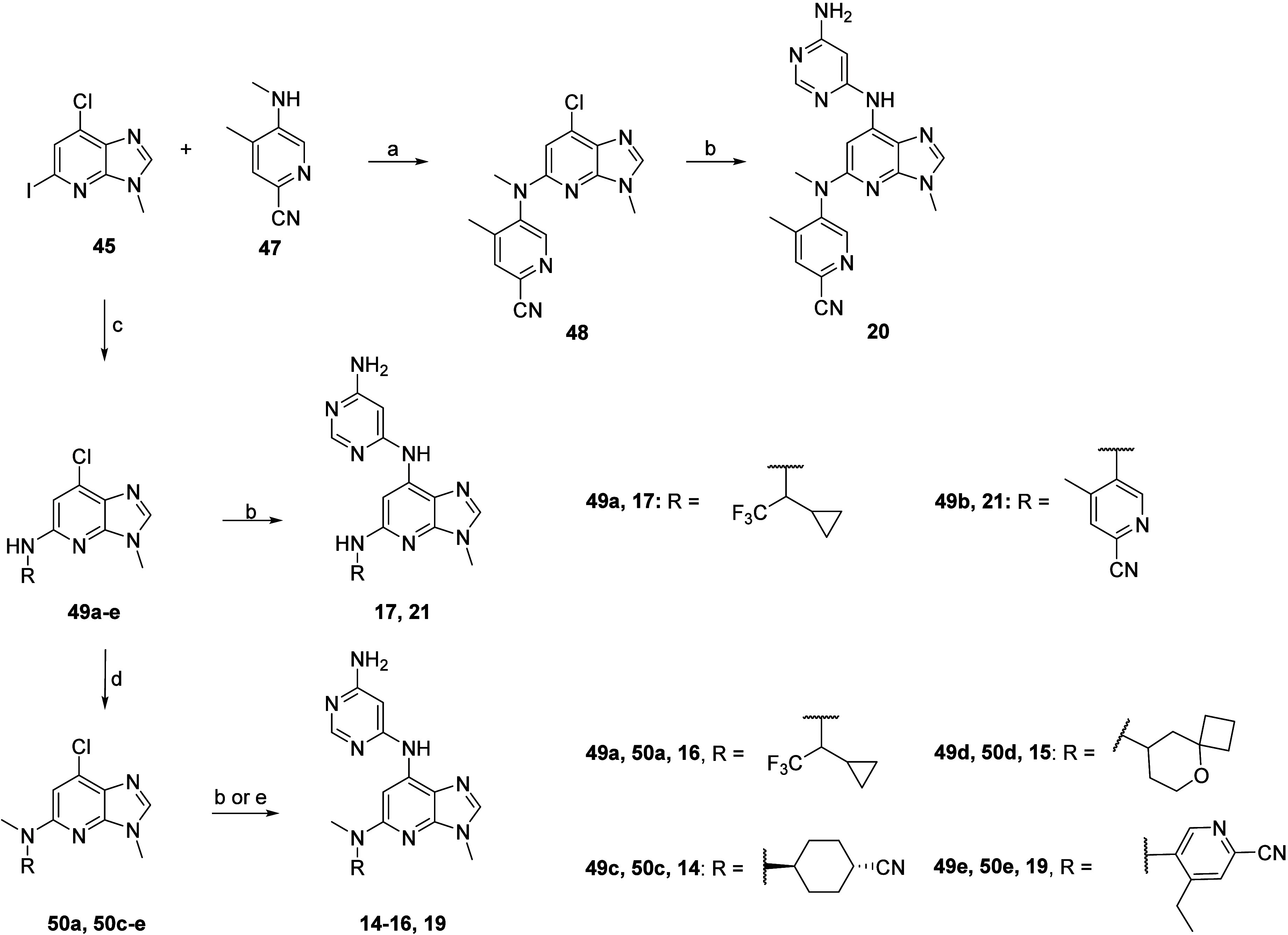
Synthesis of **14**, **15**, **16**, **17**, **19**, **20**, and **21** Reagents and conditions: (a) RuPhos, RuPhos Pd G3, K_3_PO_4_, 1,4-dioxane, 110 °C, 18 h, 43%; (b) pyrimidine-4,6-diamine, MorDalPhos, MorDalPhos Pd G3, Cs_2_CO_3_, 1,4-dioxane, 110 °C, 18 h; (c) for **49a** and **49d**: RNH_2_, XantPhos, XantPhos Pd G3, Cs_2_CO_3_, 1,4-dioxane, 110 °C, 16 h, 39–65%; for **49b**: **2b**, XantPhos, XantPhos Pd G3, Cs_2_CO_3_, 1,4-dioxane, 70 °C, 18 h, 78%; for **49c**: RNH_2_, XantPhos, XantPhos Pd G3, K_3_PO_4_, diglyme, 110 °C, 18 h; for **49e**: **3b**, XantPhos, XantPhos Pd G3, K_3_PO_4_, diglyme, 80 °C, 18 h; (d) NaH, MeI, 0 °C to rt; (e) for **19**: pyrimidine-4,6-diamine, tBuXPhos, tBuXPhos Pd G3, K_3_PO_4_, 1,4-dioxane, 110 °C, 18 h, 73%.

The synthesis of compounds listed in Table 2 was completed as shown in Scheme 4. **20** was synthesized in two steps starting via Buchwald–Hartwig arylamination between **45** and readily prepared methylated aniline **47** from commercially available 5-amino-2-chloro-4-methyl-pyridine subjected to palladium-catalyzed cyanation followed by methylation using methyl iodide (Scheme S2, Supporting Information). Final Buchwald–Hartwig coupling of chloro-intermediate **48** with pyrimidine-4,6-diamine in the presence of MorDalPhos, MorDalPhos precatalyst, and Cs_2_CO_3_ yielded **20**. For **17** and **21**, respectively, initial Buchwald–Hartwig cross-coupling of core **45** with commercially available racemic 1-cyclopropyl-2,2,2-trifluoroethan-1-amine or with readily prepared aniline **2b** (Scheme S2, Supporting Information) led to the aryl chloride intermediates **49a** and **49b**, which were subjected to an additional Buchwald–Hartwig cross-coupling with pyrimidine-4,6-diamine to yield the desired analogs. For analogs **14**, **15**, **16**, and **19**, an additional methylation step was performed on the aryl chloride intermediates **49a**, **49c**, **49d**, and **49e**, which were also obtained using commercially available alkyl amines or using readily prepared aniline **3b** obtained from commercially available 6-bromo-4-ethylpyridin-3-amine subjected to palladium-catalyzed cyanation (Scheme S3, Supporting Information), using sodium hydride as base and methyl iodide as alkylating agent, to yield **50a**, **50c**, **50d**, and **50e**, prior to final Buchwald–Hartwig cross-coupling with pyrimidine-4,6-diamine to yield desired analogs.

**Scheme 5:**
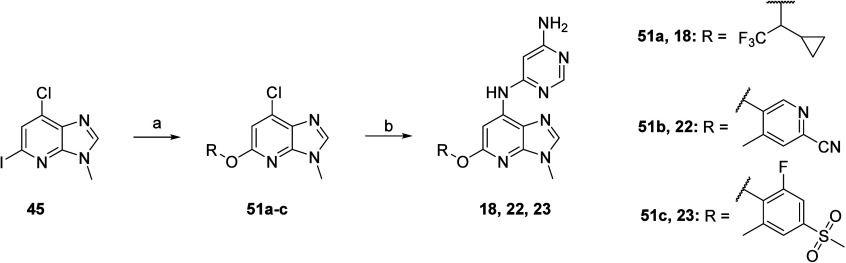
Synthesis of **18**, **22**, and **23** Reagents and conditions: (a) For **51a**: ROH, CuI, 3,4,7,8-tetramethyl-1,10-phenanthroline, Cs_2_CO_3_, DMF, 80 °C, 3 h; for **51b** (using **4b**) and **51c** (using **5e**), CuI, 2,2,6,6-tetramethyl-3,5-heptanedione, Cs_2_CO_3_, DMF, 85 °C, 72 h; (b) pyrimidine-4,6-diamine, MorDalPhos, MorDalPhos Pd G3, Cs_2_CO_3_, 1,4-dioxane, 110 °C, 18 h.

The synthesis of O-linked analogs **18**, **22**, and **23** is described in Scheme 5. The first steps involved a copper-catalyzed Ullmann coupling of core **45** with commercially available aliphatic racemic 1-cyclopropyl-2,2,2-trifluoroethan-1-ol or with readily prepared phenols **4b**, obtained via Buchwald–Hartwig cross-coupling of commercially available 5-bromo-2-cyano-4-methylpyridine with cesium hydroxide or with **5e**, obtained from 2-fluoro-4-methylsulfonyl-phenol (Scheme S4 and Scheme S5, Supporting Information). Last stage Buchwald–Hartwig cross-coupling with pyrimidine-4,6-diamine on the different chloro-intermediates **51a, 51b**, and **51c** yielded analogs **18**, **22**, and **23**.

**Scheme 6:**
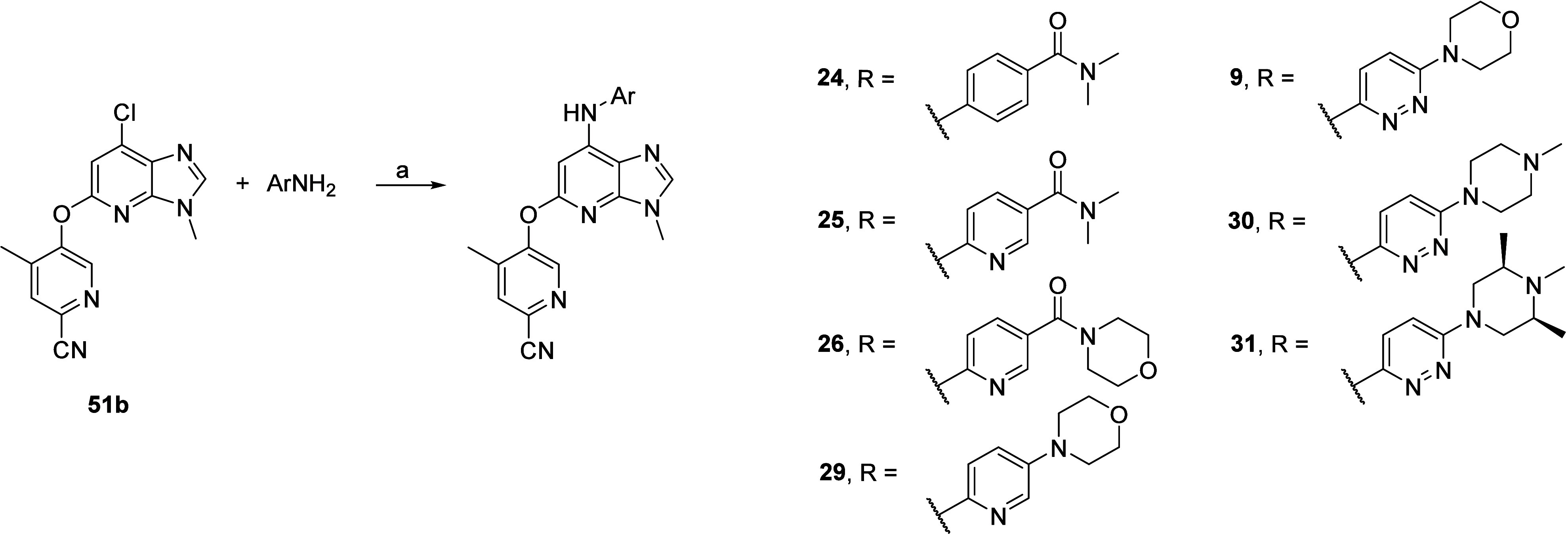
Synthesis of Compounds **9**, **24**, **25**, **26**, **29**, **30**, and **31** Reagents and conditions: (a) For **24**–**25**: ArNH_2_, MorDalPhos, MorDalPhos Pd G3, Cs_2_CO_3_, 1,4-dioxane, 110 °C, 18 h; for **9**, **26**, **30** (using **6c**), and **31** (using **7c**): ArNH_2_, MorDalPhos, [(Allyl)PdCl]_2_, Cs_2_CO_3_, 1,4-dioxane, 110 °C, 18 h; for **29**: ArNH_2_, MorDalPhos Pd G4, Cs_2_CO_3_, 1,4-dioxane, 110 °C, 18 h.

Intermediate **51b** was also key to synthesizing the compounds listed in Table 3 via Buchwald–Hartwig cross-coupling with commercially available carboxamides and anilines to afford analogs **24**, **25**, **26**, **29**, and **9** (Scheme 6). Specifically, for **30** and **31** analogs, the required 6-(4-methylpiperazin-1-yl)pyridazin-3-amine **6c** and pyrazine 6-((3*R*,5*S*)-3,4,5-trimethylpiperazin-1-yl)pyridazin-3-amine **7c** (Scheme S6 and S7, Supporting Information) were readily prepared using copper-catalyzed Ullmann coupling between commercially available 6-iodopyridazin-3-amine and relevant commercially available piperazine.

**Scheme 7:**
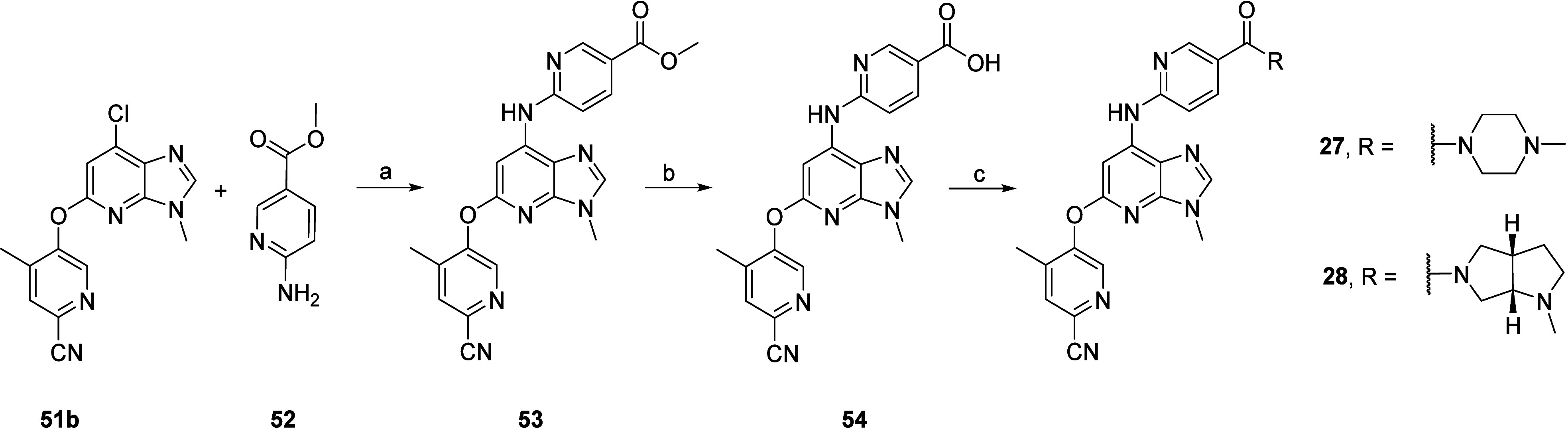
Synthesis of Compounds **27** and **28** Reagents and conditions: (a) MorDalPhos, [(Allyl)PdCl]_2_, Cs_2_CO_3_, 1,4-dioxane, 110 °C, 75%; (b) LiI, pyridine, 115 °C, 24 h, quantitative; (c) RNH_2_, HATU, Et_3_N, NMP, rt, 4–18 h.

Finally, compounds **27** and **28** were obtained by late-stage amide coupling with **54**, which was obtained via Buchwald–Hartwig cross-coupling of intermediate **51b** with methyl 6-aminonicotinate **52**, followed by LiI-promoted saponification (Scheme 7).^56^

## Conclusions

TYK2-selective compounds belonging to an 3*H*-imidazo[4,5*-b*]pyridine series were obtained by scaffold hopping from 3*H*-imidazo[4,*5-c*]pyridine-based compounds possessing dual activity against JAK1/TYK2. During SAR optimization, potency and selectivity toward TYK2 was increased. Modification of the linker between the core and an aromatic subunit improved metabolic stability, and further refinements, produced through derivatization of a vector protruding toward the solvent front, led to the discovery of **9** (GLPG3667).

TYK2 selectivity for GLPG3667 was confirmed in PBMC and whole blood assays. GLPG3667 also reduced dermal ear inflammation in a mouse IL-23-induced model of psoriasis. The PK profile in preclinical species was favorable. A phase 1b study of GLPG3667 in patients with moderate-to-severe psoriasis is completed and reported clinical efficacy,^58^ and GLPG3667 is now in phase 2 trials for the treatment of dermatomyositis (NCT05695950) and SLE (NCT05856448).

## Experimental Section

### General Experimental Methods

All reagents were of commercial grade and were used as received without further purification, unless otherwise stated. Commercially available anhydrous solvents were used for reactions conducted under nitrogen atmosphere. Reagent-grade solvents were used in all other cases, unless otherwise specified. Silica flash chromatography was performed on silica gel 60 (35–70 μm). Thin-layer chromatography was carried out using precoated silica gel F-254 plates (thickness 0.25 mm). Purification with preparatory high-performance liquid chromatography (HPLC) was performed with a Waters FractionLynx system coupled to a 2996 photodiode-array detection detector and a Waters mass detector QDa (quadrupole Dalton). For basic method, column used: Waters XBridge prep (C18, 10 μm OBD, 19 × 100 mm), eluent used: water +0.5% NH_3_, for acidic method, column used: Waters XSelect CSH (C18, 5 μm OBD, 19 × 100 mm), eluent used: water +0.1% HCOOH. Flow rate: 20 mL/min. ^1^H and ^13^C NMR spectra were recorded on a Bruker DPX 300 spectrometer (300 MHz) and a Bruker DPX 400 NMR spectrometer (400 MHz). Chemical shifts (δ) for ^1^H NMR spectra are reported in parts per million (ppm) relative to tetramethyl silane (δ 0.00) or the appropriate residual solvent peak, i.e., DMSO (δ 2.50), as internal reference. Multiplicities are given as singlet (s), doublet (d), triplet (t), quartet (q), multiplet (m), and broad (br). Ultraviolet and electrospray mass spectrometry spectra were obtained on a Waters Acquity H-Class UPLC coupled to a Waters mass detector QDa. Purities were determined by liquid chromatography–mass spectrometry (LCMS) analysis (UV traces determined with an Acquity PDA detector) using two methods: **Method A**: Acquity UPLC @ CSH C18 2.1 × 50 mm 1.7 μm (Waters) column; MeCN/H_2_O gradients (H_2_O contains 0.1% formic acid); **Method B**: Acquity UPLC @ BEH C18 2.1 × 50 mm 1.7 μm (Waters) column; MeCN/H_2_O gradients (H_2_O contains 13.4 mM NH_3_). All reported final compounds were analyzed with one of these analytical methods and all had purities ≥95%.

High-resolution mass spectrometry (HRMS) samples were prepared at 0.1 mg/mL concentration in MeCN either by dilution of DMSO stock solution (10 mM) or by dissolving solid compound. HRMS data were recorded by eluting samples using an Agilent 1260 Infinity II ultra-HPLC system on C18 column (Waters Acquity BEH C18, 2.1 × 150 mm, 1.7 μm particle size) linked to the Agilent 6540 UHD Q-Tof mass spectrometer. LC method was generic using 0.1% formic acid in water and 0.1% formic acid in MeCN from 3% to 97% of organic modifier over 5 min (flow rate: 0.5 mL/min). Ionization mode used was ESI positive with mass range from 100 to 3200 Da. The melting points (mp) were taken in open capillaries on a Mettler Toledo MP50 apparatus and are uncorrected.

### Dibenzyl-(5-chloro-3-methyl-3H-imidazo[4,5-*b*]pyridin-7-yl)-amine (**37**)

#### Step 1: 2,6-Dichloro-3-nitro-pyridin-4-ylamine (**33**)

2,6-dichloro-pyridin-4-ylamine **32** (3.0 g, 18.5 mmol) was added to concentrated H_2_SO_4_ (23 mL, 0.8 M) in a round-bottom flask at −5 °C. The mixture was stirred at −5 °C until a homogeneous solution was obtained, then nitric acid (1.4 mL, 22.5 mmol, 1.2 equiv) in 5 mL of H_2_SO_4_ was slowly added keeping the internal temperature below 10 °C. The mixture was stirred between 0 and 10 °C for 30 min, then heated to 80 °C for 30 min. The mixture was cooled to rt and then poured into ice. The resulting yellow suspension was neutralized by slow addition of aqueous NH_3_ to pH ∼ 4. The product was filtered and washed with ice-cold water to afford the title compound (3.37 g, 88% yield). LCMS (ESI) *m*/*z* 207.9 [M + H]^+^.

#### Step 2: Dibenzyl-(2,6-dichloro-3-nitro-pyridin-4-yl)-amine (**34**)

To a solution of 2,6-dichloro-3-nitro-pyridin-4-ylamine **33** (5.0 g, 24.1 mmol) in anhydrous DMF (240 mL, 0.1 M) was added benzyl bromide (8.6 mL, 72.5 mmol, 3 equiv) and K_2_CO_3_ (16.7 g, 120.5 mmol, 5 equiv) and the mixture was stirred at 80 °C for l h. The mixture was quenched with water and diluted with EtOAc. The two layers were separated. The organic layer was washed with a saturated solution of NaHCO_3_, separated, dried over anhydrous Na_2_SO_4_, filtered, and evaporated. The crude was purified by silica flash chromatography (PE/EtOAc: 100/0 to 80/20) to afford the title compound (8.76 g, 84% yield). LCMS (ESI) *m*/*z* 387.9 [M + H]^+^.

#### Step 3: N^4^,N^4^-Dibenzyl-6-chloro-N^2^-methyl-3-nitropyridine-2,4-diamine (**35a**) and N^4^,N^4^-Dibenzyl-6-chloro-N^2^-methyl-5-nitropyridine-2,4-diamine (**35b**)

To a mixture of dibenzyl-(2,6-dichloro-3-nitro-pyridin-4-yl)-amine **34** (6.9 g, 17.8 mmol, 1 equiv) and Cs_2_CO_3_ (5.8 g, 17.8 mmol, 1 equiv) in anhydrous THF (90 mL, 0.2 M) was added MeNH_2_ (2 M in THF, 8.9 mL, 17.8 mmol, 1 equiv) at 0 °C and the mixture was stirred at rt for 24 h. Reaction mixture was then concentrated *in vacuo* and the residue was dissolved in DCM and washed twice with water and brine. The two layers were separated. The organic layer was dried over anhydrous Na_2_SO_4_, filtered, and concentrated under reduced pressure to afford crude title regioisomer products used in the next step without further purification. LCMS (ESI) *m*/*z* 383.0 [M + H]^+^.

#### Step 4: N^4^,N^4^-Dibenzyl-6-chloro-N^2^-methyl-pyridine-2,3,4-triamine (**36a**) and N^4^,N^4^-Dibenzyl-6-chloro-N^2^-methyl-5-nitropyridine-2,4-diamine (**36b**)

To a solution of *N*^*4*^,*N*^*4*^-dibenzyl-6-chloro-*N*^*2*^-methyl-3-nitro-pyridine-2,4-diamine **35a** and *N*^4^,*N*^4^-dibenzyl-6-chloro-*N*^2^-methyl-5-nitropyridine-2,4-diamine **35b** (5.92 g, 15.5 mmol, 1 equiv) in MeOH/THF (1:1) (100 mL) was added zinc (5.0 g, 77.5 mmol, 5 equiv) and NH_4_Cl (170 mg, 3 mmol, 0.2 equiv). The resulting mixture was stirred at rt. After one night, the mixture was heated to 50 °C until completion of the reaction was observed by LCMS. The reaction mixture was then cooled down to rt then filtered over Celite. The filtrate was concentrated *in vacuo* and the residue was dissolved in DCM and washed with a saturated solution of NaHCO_3_. The organic layer was separated, dried over anhydrous Na_2_SO_4_, filtered, and concentrated to afford crude title regioisomer products used in the next step without further purification. LCMS (ESI) *m*/*z* 353.0 [M + H]^+^.

#### Step 5: Dibenzyl-(5-chloro-3-methyl-3H-imidazo[4,5-*b*]pyridin-7-yl)-amine (**37**)

To a suspension of *N*^4^,*N*^4^-dibenzyl-6-chloro-*N*^2^-methyl-pyridine-2,3,4-triamine **36a** and *N*^4^,*N*^4^-dibenzyl-6-chloro-*N*^2^-methyl-5-nitropyridine-2,4-diamine **36b** (420 mg, 1.1 mmol, 1 equiv) in MeCN (5 mL, 0.2 M) was added triethyl orthoformate (365 μL, 2.2 mmol, 2 equiv) and the mixture was stirred at 80 °C for 18 h. MeCN was removed *in vacuo* and the residue was dissolved in DCM and washed with a saturated solution of NaHCO_3_. Organic phase was separated, dried over anhydrous Na_2_SO_4_, filtered, and concentrated. Crude material was purified by silica flash chromatography (PE/EtOAc: 100/0 to 70/30) to afford the title compound (220 mg, 55% yield). LCMS (ESI) *m*/*z* 363.0 [M + H]^+^.

### 4-((7-Amino-3-methyl-3H-imidazo[4,5-*b*]pyridin-5-yl)(methyl)amino)-3-ethyl-5-fluorobenzonitrile (**41**)

#### Step 1: 4-((7-(Dibenzylamino)-3-methyl-3H-imidazo[4,5-*b*]pyridin-5-yl)amino)-3-ethyl-5-fluorobenzonitrile (**39**)

A mixture of *N,N*-dibenzyl-5-chloro-3-methyl-3*H*-imidazo[4,5*-b*]pyridin-7-amine **37** (200 mg, 0.552 mmol, 1 equiv), 4-amino-3-ethyl-5-fluorobenzonitrile **38** (Scheme S1, Supporting Information; 181 mg, 1.10 mmol, 2 equiv), XantPhos (30 mg, 0.055 mmol, 0.1 equiv), XantPhos Pd G3 (52 mg, 0.055 mmol, 0.1 equiv) and Cs_2_CO_3_ (326 mg, 1.10 mmol, 2 equiv) in anhydrous 1,4-dioxane (3 mL, 0.2 M) was degassed under nitrogen atmosphere and heated for 18 h. Reaction mixture was diluted with DCM and washed with brine. The two layers were separated. The organic layer was dried over anhydrous Na_2_SO_4_, filtered, and concentrated under reduced pressure to afford the title compound as crude used in the next step without further purification. LCMS (ESI) *m*/*z* 491.3 [M + H]^+^.

#### Step 2: 4-((7-(Dibenzylamino)-3-methyl-3H-imidazo[4,5-*b*]pyridin-5-yl)(methyl)amino)-3-ethyl-5-fluorobenzonitrile (**40**)

To a solution of 4-((7-(dibenzylamino)-3-methyl-3*H*-imidazo[4,5*-b*]pyridin-5-yl)amino)-3-ethyl-5-fluorobenzonitrile **39** (202 mg, 0.56 mmol, 1 equiv) in anhydrous THF (3 mL, 0.2 M) at 0 °C was added sodium hydride (60% in mineral oil) (49 mg, 1.21 mmol, 2.2 equiv). After 5 min, methyl iodide (69 μL, 1.10 mmol, 2 equiv) was added and the reaction mixture was allowed to warm up to rt and stirred overnight. Reaction mixture was quenched with MeOH and then concentrated under reduced pressure. The residue was purified by silica flash chromatography (PE/EtOAc 80:20 to 65:35) to afford the title compound (206 mg, 74% yield). LCMS (ESI) *m*/*z* 505.3 [M + H]^+^.

#### Step 3: 4-((7-Amino-3-methyl-3H-imidazo[4,5-*b*]pyridin-5-yl)(methyl)amino)-3-ethyl-5-fluorobenzonitrile (**41**)

To a solution of 4-((7-(dibenzylamino)-3-methyl-3*H*-imidazo[4,5*-b*]pyridin-5-yl)(methyl)amino)-3-ethyl-5-fluorobenzonitrile **40** (206 mg, 0.40 mmol, 1 equiv) in DCM (2 mL, 0.2 M) at 0 °C was added triflic acid (289 μL, 3.27 mmol, 3 equiv). The solution was allowed to warm up to rt and stirred overnight. The reaction was quenched with a saturated solution of NaHCO_3_ and extracted with DCM. The two layers were separated. The organic layer was washed with a saturated solution of brine, dried over anhydrous Na_2_SO_4_, filtered, and concentrated under reduced pressure to afford crude title product used in the next step without further purification. LCMS (ESI) *m*/*z* 325.1 [M + H]^+^.

### N-(5-((4-Cyano-2-ethyl-6-fluorophenyl)(methyl)amino)-3-methyl-3H-imidazo[4,5-*b*]pyridin-7-yl)cyclopropanecarboxamide (**11**)

To a solution of 4-((7-amino-3-methyl-3*H*-imidazo[4,5*-b*]pyridin-5-yl)(methyl)amino)-3-ethyl-5-fluorobenzonitrile **41** (40 mg, 0.123 mmol, 1 equiv) and pyridine (20 μL, 0.246 mmol, 2 equiv) in DCM (2.5 mL, 0.05 M) was added cyclopropanecarbonyl chloride (15 μL, 0.148 mmol, 1.2 equiv) at rt. After 1 h, cyclopropanecarbonyl chloride (10 μL, 0.111 mmol, 0.9 equiv) was added again and stirred at rt for 2 h. The mixture was diluted with DCM and washed with an aqueous solution of NH_4_Cl. The organic layer was dried over anhydrous Na_2_SO_4_, filtered, and concentrated under reduced pressure and the crude was purified by preparatory HPLC (gradient from 25% MeCN to 50% MeCN in water/0.1% formic acid) to afford the title compound (13 mg, 27% yield). LCMS (ESI), Method B, *R*_t_ 1.37 min, purity >95%, *m*/*z* 393.1 [M + H]^+^. HRMS: Calculated mass for C_21_H_22_FN_6_O (M+H)^+^ 393,18336; found 393,1832; difference 0.42 ppm.

### 1-(5-((4-Cyano-2-ethyl-6-fluorophenyl)(methyl)amino)-3-methyl-3H-imidazo[4,5-*b*]pyridin-7-yl)-3-methylurea (**12**)

To a solution of 4-((7-amino-3-methyl-3*H*-imidazo[4,5*-b*]pyridine-5-yl)(methyl)amino)-3-ethyl-5-fluorobenzonitrile **41** (46 mg, 0.142 mmol, 1 equiv) and pyridine (57 μL, 0.710 mmol, 5 equiv) in DCM (3 mL, 0.05 M) was added *N,N*′-carbonyl-di(1,2,4-triazole) (35 mg, 0.213 mmol, 1.5 equiv) at rt. After 1 h at 50 °C, MeNH_2_ (2 M in THF, 284 μL, 0.568 mmol, 4 equiv) was added and the reaction was heated again at 50 °C for 1 h. The mixture was diluted with DCM and washed with an aqueous solution of NaHCO_3_. The organic layer was dried over anhydrous Na_2_SO_4_, filtered, and concentrated under reduced pressure. The residue was triturated with MeCN and the resulting precipitate was filtered off and was purified by preparatory HPLC (gradient from 30% MeCN to 55% MeCN in water/0.5% NH_3_) to afford the title compound (6 mg, 11% yield). LCMS (ESI) Method B, *R*_t_ 1.27 min, purity >99%, *m*/*z* 382.1 [M + H]^+^. HRMS: Calculated mass for C_19_H_21_FN_7_O (M+H)^+^ 382.17861; found 382.17863; difference 0.04 ppm. ^1^H NMR (400 MHz, DMSO-*d*_*6*_) δ 8.89 (s, 1H), 7.94 (s, 1H), 7.86 (dd, *J* = 9.8 Hz, 1.9 Hz, 1H), 7.80 (s, 1H), 7.00 (s, 1H), 6.92 (q, *J* = 4.6 Hz, 1H), 3.67 (s, 3H), 3.31 (s, 3H), 2.59 (d, *J* = 4.5 Hz, 3H), 2.55 (q, *J* = 7.5 Hz, 2H), 1.12 (t, *J* = 7.6 Hz, 3H).

### 4-((7-((6-Aminopyrimidin-4-yl)amino)-3-methyl-3H-imidazo[4,5-*b*]pyridin-5-yl)(methyl)amino)-3-ethyl-5-fluorobenzonitrile (**13**)

A mixture of 4-((7-amino-3-methyl-3*H*-imidazo[4,5*-b*]pyridin-5-yl)(methyl)amino)-3-ethyl-5-fluorobenzonitrile **41** (173 mg, 0.534 mmol, 1 equiv), 6-chloropyrimidin-4-amine (207 mg, 1.60 mmol, 3 equiv), BrettPhos Pd G3 (48 mg, 0.053 mmol, 0.1 equiv), BrettPhos (28 mg, 0.053 mmol, 0.1 equiv) and Cs_2_CO_3_ (348 mg, 1.07 mmol, 2 equiv) in anhydrous 1,4-dioxane (2.7 mL, 0.2 M) was degassed under nitrogen atmosphere and heated at 110 °C for 18 h. The mixture was diluted with DCM and washed with water. The organic layer was dried over anhydrous Na_2_SO_4_, filtered, and concentrated under reduced pressure and the crude was purified by preparatory HPLC (gradient from 35% MeCN to 60% MeCN in water/0.5% NH_3_) to afford the title compound (6 mg, 3% yield). LCMS (ESI), Method B, *R*_t_ 1.27 min, purity >99%, *m*/*z* 418.2 [M + H]^+^. ^1^H NMR (400 MHz, DMSO-*d*_*6*_) δ 9.22 (s, 1H), 7.96 (s, 1H), 7.86 (dd, *J* = 9.7, 1.9 Hz, 1H), 7.83 (s, 1H), 7.81 (s, 1H), 7.35 (s, 1H), 6.45 (s, 2H), 6.16 (s, 1H), 3.68 (s, 3H), 3.35 (s, 3H), 2.58 (q, *J* = 7.5 Hz, 2H), 1.14 (t, *J* = 7.5 Hz, 3H).

### 7-Chloro-5-iodo-3-methyl-3H-imidazo[4,5-*b*]pyridine (**45**)

#### Step 1: 2,4-Dichloro-6-iodo-pyridin-3-ylamine (**43**)

To a solution of 2,4-dichloro-3-aminopyridine **42** (1 g, 6.17 mmol, 1 equiv) in anhydrous THF (12 mL, 0.5 M) under N_2_ atmosphere at rt was added *N*-iodosuccinimide (1.53 g, 6.79 mmol, 1.1 equiv) and TFA (142 μL, 1.85 mmol, 0.3 equiv). The mixture was stirred at 40 °C for 18 h. The reaction mixture was then quenched with a saturated solution of Na_2_S_2_O_3_ and diluted with EtOAc. The organic phase was separated and further washed with a saturated solution of NaHCO_3_. The two layers were separated. The organic layer was dried over anhydrous Na_2_SO_4_, filtered, and concentrated under reduced pressure. The crude was purified by silica flash chromatography (cyclohexane/EtOAc: 90/10) to afford the title compound (1.56 g, 88% yield). LCMS (ESI) *m*/*z* 289.9 [M + H]^+^.

#### Step 2: 4-Chloro-6-iodo-N2-methyl-pyridine-2,3-diamine (**44**)

In a sealed tube, to a solution of 2,4 -dichloro-6-iodo-pyridin-3-amine **43** (2.74 g, 9.51 mmol, 1 equiv) in anhydrous NMP (9.5 mL, 1 M) was added methylamine (2 M in THF, 9.5 mL, 19.0 mmol, 2 equiv) under N_2_ at rt. The mixture was stirred at 180 °C for 18 h and then cooled to rt. The reaction mixture was then quenched with water and diluted with EtOAc. The organic phase was separated and further washed with a saturated solution of brine. The two layers were separated. The organic layer was dried over anhydrous Na_2_SO_4_, filtered, and concentrated under reduced pressure. The crude was purified by silica flash chromatography (PE/EtOAc: 80/20 to 75/25) to afford the title compound (1.63 g, 61% yield). LCMS (ESI) *m*/*z* 283.8 [M + H]^+^.

#### Step 3: 7-Chloro-5-iodo-3-methyl-3H-imidazo[4,5-*b*]pyridine (**45**)

To a solution of 4-chloro-6-iodo-*N*-2-methyl-pyridine-2,3-diamine **44** (20.7 g, 73.1 mmol, 1 equiv) in formic acid (30 mL) was added trimethyl orthoformate (24.0 mL, 219 mmol, 3 equiv). The mixture was stirred at 60 °C for 1 h. Reaction was concentrated to dryness after which the residue was diluted with DCM and quenched with a saturated solution of NaHCO_3_. The organic phase was separated and further washed with a saturated solution of brine. The two layers were separated. The organic layer was dried over anhydrous Na_2_SO_4_, filtered, and concentrated under reduced pressure. The crude was triturated in MTBE. Solid was isolated by filtration and dried under reduced pressure to afford the title compound (19.0 g, 89% yield). LCMS (ESI) *m*/*z* 293.9 [M + H]^+^. ^1^H NMR (300 MHz, DMSO-*d*_*6*_) δ 8.46 (s, 1H), 7.83 (s, 1H), 3.81 (s, 3H).

### 5-[(7-Chloro-3-methyl-3H-imidazo[4,5-*b*]pyridin-5-yl)-methylamino]-4-methylpyridine-2-carbonitrile (**48**)

A mixture of 7-chloro-5-iodo-3-methyl-3*H*-imidazo[4,5*-b*]pyridine **45** (375 mg, 1.28 mmol, 1 equiv), 4-methyl-5-(methylamino) pyridine-2-carbonitrile **47** (Scheme S2, Supporting Information; 226 mg, 1.54 mmol, 1.2 equiv), RuPhos Pd G3 (32 mg, 0.004 mmol, 0.03 equiv), RuPhos (18 mg, 0.004 mmol, 0.03 equiv) and K_3_PO_4_ (543 mg, 2.56, 2 equiv) in anhydrous 1,4-dioxane (6 mL, 0.2 M) was degassed under nitrogen atmosphere and heated for 18 h at 110 °C. The reaction was reloaded with RuPhos Pd G3 (32 mg, 0.004 mmol, 0.03 equiv) and RuPhos (18 mg, 0.004 mmol, 0.03 equiv), degassed under nitrogen atmosphere, and heated for an additional 3 h at 110 °C. The reaction mixture was diluted with EtOAc and washed with brine. The two layers were separated. The organic layer was dried over anhydrous Na_2_SO_4_, filtered, and concentrated under reduced pressure. Crude material was purified by silica flash chromatography (EtOAc/PE, 50:50 to 100:0) to afford the title compound (172 mg, 43% yield). LCMS (ESI) *m*/*z* 313.1 [M + H]^+^.

### General Procedure for the Preparation of **49a** and **49d**

#### 7-Chloro-N-(1-cyclopropyl-2,2,2-trifluoroethyl)-3-methyl-3H-imidazo[4,5-*b*]pyridin-5-amine (**49a**)

A mixture of 7-chloro-5-iodo-3-methyl-3H-imidazo[4,5-*b*]pyridine **45** (250 mg, 0.852 mmol, 1 equiv), 1-cyclopropyl-2,2,2-trifluoroethan-1-amine (165 mg, 0.937 mmol, 1.1 equiv), XantPhos Pd G3 (24 mg, 0.03 mmol, 0.03 equiv), XantPhos (15 mg, 0.03 mmol, 0.03 equiv) and Cs_2_CO_3_ (833 mg, 2.556 mmol, 3 equiv) in 1,4-dioxane (4 mL, 0.2 M) was degassed under inert atmosphere and heated at 110 °C for 16 h. Reaction mixture was diluted with DCM and washed with brine. The two layers were separated. The organic layer was dried on Na_2_SO_4_, filtered, and concentrated under reduced pressure. Crude material was purified by silica flash chromatography (EtOAc/cyclohexane, 70:30 to 100:0) to afford the title compound (191 mg, 65% yield). LCMS (ESI) *m*/*z* 305.0 [M + H]^+^.

#### 7-Chloro-3-methyl-N-(5-oxaspiro[3.5]nonan-8-yl)-3H-imidazo[4,5-*b*]pyridin-5-amine (**49d**)

This compound was prepared from **45** and 5-oxaspiro[3.5]nonan-8-amine according to the general procedure used for the preparation of **49a**. 39% yield. LCMS (ESI) *m*/*z* 307.1 [M + H]^+^.

#### 5-(7-Chloro-3-methyl-3H-imidazo[4,5-*b*]pyridin-5-ylamino)-4-methyl-pyridine-2-carbonitrile (**49b**)

A mixture of 7-chloro-5-iodo-3-methyl-3*H*-imidazo[4,5*-b*]pyridine **45** (300 mg, 1.02 mmol, 1.0 equiv), 5-amino-4-methylpyridine-2-carbonitrile **2b** (Scheme S2, Supporting Information; 200 mg, 1.54 mmol, 1.5 equiv), XantPhos Pd G3 (29 mg, 0.03 mmol, 0.03 equiv), XantPhos (18 mg, 0.03 mmol, 0.03 equiv) and Cs_2_CO_3_ (998 mg, 3.07 mmol, 3 equiv) in anhydrous 1,4-dioxane (2 mL, 0.5 M) was degassed under nitrogen atmosphere and heated at 70 °C for 18 h. The reaction mixture was diluted with DCM and washed with brine. The two layers were separated. The organic layer was dried over anhydrous Na_2_SO_4_, filtered, and concentrated under reduced pressure. Crude material was purified by silica flash chromatography (EtOAc/PE, 70:30 to 100:0) to afford the title compound (238 mg, 78% yield). LCMS (ESI) *m*/*z* 299.0 [M + H]^+^.

#### (1r,4r)-4-((7-Chloro-3-methyl-3H-imidazo[4,5-*b*]pyridin-5-yl)amino)cyclohexane-1-carbonitrile (**49c**)

A mixture of 7-chloro-5-iodo-3-methyl-3*H*-imidazo[4,5*-b*]pyridine **45** (100 mg, 0.34 mmol, 1.0 equiv), *trans*-4-aminocyclohexanecarbonitrile hydrochloride (66 mg, 0.41 mmol, 1.2 equiv), XantPhos Pd G3 (10 mg, 0.01 mmol, 0.03 equiv), XantPhos (6 mg, 0.01 mmol, 0.03 equiv) and K_3_PO_4_ (144 mg, 0.68 mmol, 2 equiv) in diglyme (1.1 mL, 0.3 M) was degassed under nitrogen atmosphere and heated at 110 °C for 18 h. Reaction mixture was diluted with DCM and washed with brine. The two layers were separated. The organic layer was dried over anhydrous Na_2_SO_4_, filtered, and concentrated under reduced pressure. Crude reaction mixture used as such in the next step. LCMS (ESI) *m*/*z* 290.1 [M + H]^+^.

#### 5-((7-Chloro-3-methyl-3H-imidazo[4,5-*b*]pyridin-5-yl)amino)-4-ethylpicolinonitrile (**49e**)

A mixture of 7-chloro-5-iodo-3-methyl-3*H*-imidazo[4,5*-b*]pyridine **45** (100 mg, 0.34 mmol, 1.0 equiv), 5-amino-4-ethyl-pyridine-2-carbonitrile **3b** (Scheme S3, Supporting Information; 60 mg, 0.41 mmol, 1.2 equiv), XantPhos Pd G3 (10 mg, 0.01 mmol, 0.03 equiv), XantPhos (6 mg, 0.01 mmol, 0.03 equiv) and K_3_PO_4_ (144 mg, 0.68 mmol, 2 equiv) in diglyme (1.1 mL, 0.3 M) was degassed under nitrogen atmosphere and heated at 80 °C for 18 h. Reaction mixture was diluted with DCM and washed with brine. The two layers were separated. The organic layer was dried over anhydrous Na_2_SO_4_, filtered, and concentrated under reduced pressure. Crude material was purified by silica flash chromatography (EtOAc/PE, 70:30 to 100:0) to afford the title compound (78 mg, 73% yield). LCMS (ESI) *m*/*z* 313.0 [M + H]^+^.

### General Procedure for the Preparation of **50a**, **50c**, **50d**, and **50e**

#### (1r,4r)-4-((7-Chloro-3-methyl-3H-imidazo[4,5-*b*]pyridin-5-yl)(methyl)amino)cyclohexane-1-carbonitrile (**50c**)

To a solution of (1*r*,4*r*)-4-((7-chloro-3-methyl-3*H*-imidazo[4,5*-b*]pyridin-5-yl)amino)cyclohexane-1-carbonitrile **49c** (24 mg, 0.083 mmol, 1 equiv) in anhydrous THF (1 mL, 0.1 M) at 0 °C was added sodium hydride (60% in mineral oil) (5 mg, 0.125 mmol, 1.5 equiv). After 10 min, methyl iodide (7 μL, 0.108 mmol, 1.3 equiv) was added and the reaction mixture heated at 40 °C for 16 h. The reaction was quenched with MeOH and concentrated under reduced pressure. Crude material was purified by silica flash chromatography (EtOAc/PE, 60:40 to 100:0) to afford the title compound (9 mg, 36% yield). LCMS (ESI) *m*/*z* 304.1 [M + H]^+^.

#### 7-Chloro-N-(1-cyclopropyl-2,2,2-trifluoroethyl)-N,3-dimethyl-3H-imidazo[4,5-*b*]pyridin-5-amine (**50a**)

This compound was prepared from **49a** according to the general procedure used for the preparation of **50c**. 79% yield. LCMS (ESI) *m*/*z* 319.0 [M + H]^+^.

#### 7-Chloro-N,3-dimethyl-N-(5-oxaspiro[3.5]nonan-8-yl)-3H-imidazo[4,5-*b*]pyridin-5-amine (**50d**)

This compound was prepared from **49d** according to the general procedure used for the preparation of **50c**. 7% yield. LCMS (ESI) *m*/*z* 321.1 [M + H]^+^.

#### 5-((7-Chloro-3-methyl-3H-imidazo[4,5-*b*]pyridin-5-yl)(methyl)amino)-4-ethylpyridine-2-carbonitrile (**50e**)

This compound was prepared from **49e** according to the general procedure used for the preparation of **50c**. Crude reaction mixture used as such in the next step. LCMS (ESI) *m*/*z* 327.0 [M + H]^+^.

#### (7-Chloro-3-methyl-3H-imidazo[4,5-*b*]pyridin-5-yl)-(l-cyclopropyl-2,2,2-trifluoroethyl)-amine (**51a**)

To a mixture of 7-chloro-5-iodo-3-methyl-3*H*-imidazo[4,5*-b*]pyridine **45** (450 mg, 1.53 mmol, 1 equiv), CuI (29 mg, 0.153 mmol, 0.1 equiv), 3,4,7,8-tetramethyl-l,10-phenanthroline (72 mg, 0.30 mmol, 0.2 equiv) and Cs_2_CO_3_ (1.00 g, 3.06 mmol, 2 equiv) in DMF (2.2 mL, 0.7 M) was added 1-cyclopropyl-2,2,2-trifluoroethan-1-ol (858 mg, 6.13 mmol, 4 equiv) and the mixture was heated at 80 °C for 3 h. The reaction mixture was diluted with EtOAc and washed with brine. The two layers were separated, and the organic layer was dried over anhydrous Na_2_SO_4_ and concentrated. The resulting crude was purified by silica flash chromatography (PE/EtOAc 80/20 to 20/80) to afford the title compound (300 mg, 64% yield). LCMS (ESI) *m*/*z* 306.1 [M + H]^+^.

### General Procedure for the Preparation of **51b** and **51c**

#### 5-(7-Chloro-3-methyl-3H-imidazo [4,5-*b*]pyridin-5-yloxy)-4-methyl-pyridine-2-carbonitrile (**51b**)

A mixture of 7-chloro-5-iodo-3-methyl-3*H*-imidazo[4,5*-b*]pyridine **45** (68.5 g, 234 mmol, 1 equiv), 5-hydroxy-4-methylpyridine-2-carbonitrile **4b** (Scheme S4, Supporting Information; 47.0 g, 351 mmol, 1.5 equiv), CuI (8.89 g, 46.8 mmol, 0.2 equiv), 2,2,6,6-tetramethylheptane-3,5-dione (97.4 mL, 468 mmol, 2 equiv) and Cs_2_CO_3_ (152 g, 468 mmol, 2 equiv) in DMF (234 mL, 1 M) was stirred at 85 °C under air for 48 h. Additional CuI (4.45 g, 23.4 mmol, 0.1 equiv) and 2,2,6,6-tetramethylheptane-3,5-dione (48.7 mL, 234 mmol, 1 equiv) were added, after which the mixture was stirred further at 85 °C for another 24 h. The mixture was cooled to 0 °C and the resulting thick paste was then filtered and the cake was washed with ice-cooled DMF (2 × 20 mL) and then ice-cooled MTBE (3 × 150 mL). After drying the cake was stirred in a 10% aqueous solution of TMEDA for 2 h. The solid was filtrated and washed with H_2_O, then dried to afford the title compound (41.62 g, 60% yield). LCMS (ESI) *m*/*z* 300.0 [M + H]^+^.

#### N-[5-(2-Fluoro-4-methanesulfonyl-6-methyl-phenoxy)-3-methyl-3H-imidazo[4,5-*b*]pyridin-7-yl]-pyrimidine-4,6-diamine (**51c**)

This compound was prepared from **45** and 2-fluoro-6-methyl-4-methylsulfonyl-phenol **5e** (Scheme S5, Supporting Information) according to the general procedure used for the preparation of **51b**. 19% yield. LCMS (ESI) *m*/*z* 370.1 [M + H]^+^.

### General Procedure for the Preparation of **14**, **15**, **16**, **17**, **18**, **20**, **21**, **22**, **23**, **24**, and **25**

#### (1r,4r)-4-((7-((6-Aminopyrimidin-4-yl)amino)-3-methyl-3H-imidazo[4,5-*b*]pyridin-5-yl)(methyl)amino)cyclohexane-1-carbonitrile (**14**)

A mixture of (1*r*,4*r*)-4-((7-chloro-3-methyl-3*H*-imidazo[4,5*-b*]pyridin-5-yl)(methyl)amino)cyclohexane-1-carbonitrile **50c** (9 mg, 0.030 mmol, 1 equiv), pyrimidine-4,6-diamine (7 mg, 0.060 mmol, 2 equiv), MorDalPhos Pd G3 (2.5 mg, 0.003 mmol, 0.1 equiv), MorDalPhos (1.4 mg, 0.003 mmol, 0.1 equiv) and Cs_2_CO_3_ (20 mg, 0.060 mmol, 2 equiv) in anhydrous 1,4-dioxane (1 mL, 0.03 M) was degassed under nitrogen atmosphere and heated at 110 °C for 18 h. The mixture was filtered over Celite, concentrated, and the crude was purified by preparatory HPLC (gradient from 20% MeCN to 45% MeCN in water/0.5% NH_3_) to afford the title compound (2 mg, 18% yield). LCMS (ESI), Method B, *R*_t_ 1.10 min, purity >99%, *m*/*z* 378.1 [M + H]^+^. ^1^H NMR (400 MHz, DMSO-*d*_*6*_) δ 9.02 (s, 1H), 8.15 (d, *J* = 1.0 Hz, 1H), 7.88 (s, 1H), 7.61 (s, 1H), 6.46 (s, 2H), 6.22 (d, *J* = 1.1 Hz, 1H), 4.47–4.33 (m, 1H), 3.68 (s, 3H), 2.87 (s, 3H), 2.76–2.64 (m, 1H), 2.19–2.11 (m, 2H), 1.75–1.54 (m, 6H).

#### N^7^-(6-Aminopyrimidin-4-yl)-N^5^,3-dimethyl-N^5^-(5-oxaspiro[3.5]nonan-8-yl)-3H-imidazo[4,5-*b*]pyridine-5,7-diamine (**15**)

This compound was prepared from **50d** and pyrimidine-4,6-diamine according to the general procedure used for the preparation of **14**. 60% yield. LCMS (ESI), Method B, *R*_t_ 1.15 min, purity >99%, *m*/*z* 395.2 [M + H]^+^. HRMS: Calculated mass for C_20_H_27_N_8_O (M+H)^+^ 395.23023; found 395.23008; difference 0.39 ppm. ^1^H NMR (400 MHz, DMSO-*d*_*6*_) δ 9.05 (s, 1H), 8.11 (d, *J* = 1.0 Hz, 1H), 7.88 (s, 1H), 7.71 (s, 1H), 6.46 (s, 2H), 6.24 (d, *J* = 1.0 Hz, 1H), 4.66–4.55 (m, 1H), 3.77 (dd, *J* = 11.8, 4.1 Hz, 1H), 3.67 (s, 3H), 3.50 (dd, *J* = 12.3, 10.1 Hz, 1H), 2.92 (s, 3H), 2.34–2.25 (m, 1H), 2.10–1.83 (m, 4H), 1.82–1.48 (m, 5H).

#### N^7^-(6-Aminopyrimidin-4-yl)-N^5^-(1-cyclopropyl-2,2,2-trifluoroethyl)-N^5,^3-dimethyl-3H-imidazo[4,5-*b*]pyridine-5,7-diamine (**16**)

This compound was prepared from **50a** and pyrimidine-4,6-diamine according to the general procedure used for the preparation of **14**. 25% yield. LCMS (ESI), Method B, *R*_t_ 1.27 min, purity >99%, *m*/*z* 393.2 [M + H]^+^. HRMS: Calculated mass for C_17_H_20_F_3_N_8_ (M+H)^+^ 393.17575; found 393.1749; difference 2.18 ppm. ^1^H NMR (400 MHz, DMSO-*d*_*6*_) δ 9.20 (s, 1H), 8.17 (d, *J* = 0.9 Hz, 1H), 7.94 (s, 1H), 7.74 (s, 1H), 6.51 (s, 2H), 6.26 (d, *J* = 1.1 Hz, 1H), 5.07–4.94 (m, 1H), 3.64 (s, 3H), 3.07 (s, 3H), 1.48–1.37 (m, 1H), 0.87–0.76 (m, 1H), 0.70–0.61 (m, 1H), 0.60–0.52 (m, 1H), 0.25–0.16 (m, 1H). ^13^C NMR (101 MHz, DMSO-*d*_*6*_) δ 164.48, 160.52, 157.96, 156.92, 146.03, 141.23, 139.93, 126.98 (q, *J* = 286.6 Hz), 119.46, 90.06, 88.26, 59.88 (q, *J* = 27.7 Hz), 32.28, 29.37, 8.32, 5.98, 2.51. mp = 221.7–222.7 °C.

#### N^7^-(6-Aminopyrimidin-4-yl)-N^5^-(1-cyclopropyl-2,2,2-trifluoroethyl)-3-methyl-3H-imidazo[4,5-*b*]pyridine-5,7-diamine (**17**)

This compound was prepared from **49a** and pyrimidine-4,6-diamine according to the general procedure used for the preparation of **14**. 69% yield. LCMS (ESI), Method B, *R*_t_ 1.16 min, purity >99%, *m*/*z* 379.2 [M + H]^+^. HRMS: Calculated mass for C_16_H_18_F_3_N_8_ (M+H)^+^ 379.1601; found 379.1594; difference 1.86 ppm. ^1^H NMR (400 MHz, DMSO-*d*_*6*_) δ 9.05 (s, 1H), 8.16 (d, *J* = 1.0 Hz, 1H), 7.85 (s, 1H), 7.52 (s, 1H), 7.00 (d, *J* = 9.2 Hz, 1H), 6.49 (s, 2H), 6.21 (d, *J* = 1.1 Hz, 1H), 4.60 (q, *J* = 8.3 Hz, 1H), 3.64 (s, 3H), 1.21–1.09 (m, 1H), 0.65–0.55 (m, 1H), 0.52–0.37 (m, 3H). ^13^C NMR (101 MHz, DMSO-*d*_*6*_) δ 164.43, 160.52, 157.90, 156.25, 145.94, 140.48, 138.84, 127.07 (q, *J* = 284.3 Hz), 119.59, 92.60, 88.14, 54.36 (q, *J* = 28.4 Hz), 29.44, 10.41 (d, *J* = 2.2 Hz), 3.15, 1.91. mp >250 °C.

#### N^4^-(5-(1-Cyclopropyl-2,2,2-trifluoroethoxy)-3-methyl-3H-imidazo[4,5-*b*]pyridin-7-yl)pyrimidine-4,6-diamine (**18**)

This compound was prepared from **51a** and pyrimidine-4,6-diamine according to the general procedure used for the preparation of **14**. 7% yield. LCMS (ESI), Method A, *R*_t_ 1.06 min, purity >99%, *m*/*z* 380.1 [M + H]^+^. HRMS: Calculated mass for C_16_H_17_F_3_N_7_O (M+H)^+^ 380.14412; found 380.14329; difference 2.19 ppm. ^1^H NMR (400 MHz, DMSO-*d*_*6*_) δ 9.52 (s, 1H), 8.22 (d, *J* = 0.9 Hz, 1H), 8.12 (s, 1H), 7.84 (s, 1H), 6.58 (s, 2H), 6.30 (d, *J* = 1.1 Hz, 1H), 5.42 (dt, *J* = 8.7, 6.8 Hz, 1H), 3.73 (s, 3H), 1.32–1.22 (m, 1H), 0.77–0.66 (m, 1H), 0.64–0.55 (m, 3H). ^13^C NMR (101 MHz, DMSO-*d*_*6*_) δ 164.55, 160.32, 159.97, 158.01, 144.15, 142.36, 141.22, 125.26 (q, *J* = 282.3 Hz), 121.79, 92.56, 88.76, 73.88 (q, *J* = 29.9 Hz), 29.75, 9.80, 2.89, 2.73. mp >250 °C.

#### 5-((7-((6-Aminopyrimidin-4-yl)amino)-3-methyl-3H-imidazo[4,5-*b*]pyridin-5-yl)(methyl)amino)-4-methylpicolinonitrile (**20**)

This compound was prepared from **48** and pyrimidine-4,6-diamine according to the general procedure used for the preparation of **14**. 14% yield. LCMS (ESI), Method B, *R*_t_ 1.08 min, purity >99%, *m*/*z* 387.2 [M + H]^+^. HRMS: Calculated mass for C_19_H_19_N_10_ (M+H)^+^ 387.17887; found 387.17881; difference 0.15 ppm. ^1^H NMR (400 MHz, DMSO-*d*_*6*_) δ 9.32 (s, 1H), 8.63 (s, 1H), 8.08 (s, 1H), 8.00 (s, 1H), 7.92 (d, *J* = 1.0 Hz, 1H), 7.43 (s, 1H), 6.49 (s, 2H), 6.18 (d, *J* = 1.0 Hz, 1H), 3.67 (s, 3H), 3.48 (s, 3H), 2.18 (s, 3H). ^13^C NMR (101 MHz, DMSO-*d*_*6*_) δ 164.42, 160.44, 157.54, 155.50, 150.73, 146.77, 146.52, 146.32, 140.85, 140.35, 131.57, 128.79, 120.05, 118.25, 92.76, 88.35, 38.49, 29.57, 18.10. mp = 136.1–137.7 °C.

#### 5-((7-((6-Aminopyrimidin-4-yl)amino)-3-methyl-3H-imidazo[4,5-*b*]pyridin-5-yl)amino)-4-methylpicolinonitrile (**21**)

This compound was prepared from **49b** and pyrimidine-4,6-diamine according to the general procedure used for the preparation of **14**. 5% yield. LCMS (ESI), Method B, *R*_t_ 1.01 min, purity >99%, *m*/*z* 373.2 [M + H]^+^. HRMS: Calculated mass for C_18_H_17_N_10_ (M+H)^+^ 373.16322; found 373.16267; difference 1.47 ppm. ^1^H NMR (400 MHz, DMSO-*d*_*6*_) δ 9.45 (s, 1H), 9.39 (s, 1H), 8.72 (s, 1H), 8.20 (d, *J* = 1.0 Hz, 1H), 8.07 (s, 1H), 8.02 (s, 1H), 7.82 (s, 1H), 6.57 (s, 2H), 6.27 (d, *J* = 1.0 Hz, 1H), 3.71 (s, 3H), 2.36 (s, 3H).

#### 5-((7-((6-Aminopyrimidin-4-yl)amino)-3-methyl-3H-imidazo[4,5-*b*]pyridin-5-yl)oxy)-4-methylpicolinonitrile (**22**)

This compound was prepared from **51b** and pyrimidine-4,6-diamine according to the general procedure used for the preparation of **14**. 7% yield. LCMS (ESI), Method B, *R*_t_ 1.06 min, purity >99%, *m*/*z* 374.2 [M + H]^+^. HRMS: Calculated mass for C_18_H_16_N_9_O (M+H)^+^ 374.14723; found 374.14773; difference 1.33 ppm. ^1^H NMR (400 MHz, DMSO-*d*_*6*_) δ 9.73 (s, 1H), 8.52 (s, 1H), 8.21 (s, 1H), 8.17 (s, 2H), 8.10 (s, 1H), 6.62 (s, 2H), 6.34 (s, 1H), 3.59 (s, 3H), 2.28 (s, 3H). ^13^C NMR (101 MHz, DMSO-*d*_*6*_) δ 164.59, 160.29, 159.68, 157.97, 153.02, 144.81, 144.76, 142.80, 142.12, 140.93, 132.08, 127.85, 122.59, 118.00, 93.70, 88.96, 29.85, 15.87. mp >250 °C.

#### N^4^-(5-(2-Fluoro-6-methyl-4-(methylsulfonyl)phenoxy)-3-methyl-3H-imidazo[4,5-*b*]pyridin-7-yl)pyrimidine-4,6-diamine (**23**)

This compound was prepared from **51c** and pyrimidine-4,6-diamine according to the general procedure used for the preparation of **14**. 8% yield. LCMS(ESI), Method B, *R*_t_ 1.07 min, purity >99%, *m*/*z* 444.2 [M + H]^+^. HRMS: Calculated mass for C_19_H_19_FN_7_O_3_S (M+H)^+^ 444.12486; found 444.12439; difference 1.07 ppm. ^1^H NMR (400 MHz, DMSO-*d*_*6*_) δ 9.68 (s, 1H), 8.21 (d, *J* = 1.0 Hz, 1H), 8.15 (s, 1H), 8.13 (s, 1H), 7.82–7.75 (m, 2H), 6.61 (s, 2H), 6.34 (d, *J* = 1.0 Hz, 1H), 3.54 (s, 3H), 3.32 (s, 3H) 2.29 (s, 3H). ^13^C NMR (101 MHz, DMSO-*d*_*6*_) δ 164.58, 160.33, 160.00, 157.95, 154.95 (d, *J* = 251.2 Hz), 144.65, 144.02 (d, *J* = 12.5 Hz), 142.65, 141.72, 138.07 (d, *J* = 6.5 Hz), 135.81, 125.60 (d, *J* = 3.0 Hz), 122.23, 113.66 (d, *J* = 22.0 Hz), 92.12, 88.92, 43.81, 29.80, 16.48 (d, *J* = 2.1 Hz). mp >250 °C.

#### 4-((5-((6-Cyano-4-methylpyridin-3-yl)oxy)-3-methyl-3H-imidazo[4,5-*b*]pyridin-7-yl)amino)-N,N-dimethylbenzamide (**24**)

This compound was prepared from **51b** and 4-amino-*N*,*N*-dimethylbenzamide according to the general procedure used for the preparation of **14**. 21% yield. LCMS (ESI), Method B, *R*_t_ 1.16 min, purity >99%, *m*/*z* 428.2 [M + H]^+^. HRMS: Calculated mass for C_23_H_22_N_7_O_2_ (M+H)^+^ 428.18295; found 428.18318; difference 0.54 ppm. ^1^H NMR (400 MHz, DMSO-*d*_*6*_) δ 9.48 (s, 1H), 8.51 (s, 1H), 8.14 (s, 1H), 8.09 (s, 1H), 7.49–7.39 (m, 4H), 6.64 (s, 1H), 3.58 (s, 3H), 2.98 (s, 6H), 2.26 (s, 3H). ^13^C NMR (101 MHz, DMSO-*d*_*6*_) δ 170.33, 160.09, 153.01, 145.49, 145.30, 144.70, 141.91, 141.74, 140.85, 132.07, 131.13, 128.85, 127.81, 122.26, 120.80, 117.99, 88.12, 29.86, 15.83.

#### 6-((5-((6-Cyano-4-methylpyridin-3-yl)oxy)-3-methyl-3H-imidazo[4,5-*b*]pyridin-7-yl)amino)-N,N-dimethylnicotinamide (**25**)

This compound was prepared from **51b** and 6-amino-*N,N*-dimethylpyridine-3-carboxamide according to the general procedure used for the preparation of **14**. 28% yield. LCMS (ESI), Method B, *R*_t_ 1.14 min, purity >99%, *m*/*z* 429.2 [M + H]^+^. HRMS: Calculated mass for C22H21N8O2 (M+H)^+^ 429.1782; found 429.17728; difference 2.14 ppm. ^1^H NMR (400 MHz, DMSO-*d*_*6*_) δ 10.16 (s, 1H), 8.54 (s, 1H), 8.45–8.39 (m, 1H), 8.29 (s, 1H), 8.20 (s, 1H), 8.11 (s, 1H), 7.79 (dd, *J* = 8.6, 2.4 Hz, 1H), 7.50 (d, *J* = 8.6 Hz, 1H), 3.60 (s, 3H), 3.00 (s, 6H), 2.29 (s, 3H). ^13^C NMR (101 MHz, DMSO-*d*_*6*_) δ 168.56, 159.93, 155.84, 153.00, 146.70, 144.92, 144.69, 142.53, 142.12, 141.02, 137.47, 132.10, 127.92, 125.16, 122.48, 118.00, 112.96, 92.74, 29.87, 15.89. mp = 165.8–166.9 °C.

#### 5-((7-((6-Aminopyrimidin-4-yl)amino)-3-methyl-3H-imidazo[4,5-*b*]pyridin-5-yl)(methyl)amino)-4-ethylpicolinonitrile (**19**)

A mixture of 5-((7-chloro-3-methyl-3*H*-imidazo[4,5*-b*]pyridin-5-yl)(methyl)amino)-4-ethylpyridine-2-carbonitrile **50e** (75 mg, 0.250 mmol, 1 equiv), pyrimidine-4,6-diamine (55 mg, 0.500 mmol, 2 equiv), *t*BuXPhos Pd G3 (20 mg, 0.025 mmol, 0.1 equiv), *t*BuXPhos (11 mg, 0.025 mmol, 0.1 equiv) and K_3_PO_4_ (106 mg, 0.500 mmol, 2 equiv) in anhydrous 1,4-dioxane (2.5 mL, 0.1 M) was degassed under nitrogen atmosphere and heated at 110 °C for 18 h. The mixture was filtered over Celite, concentrated, and the crude was purified by preparatory HPLC (gradient from 30% MeCN to 55% MeCN in water/0.5% NH_3_) to afford the title compound (45 mg, 25% yield). LCMS (ESI), Method B, *R*_t_ 1.15 min, purity >99%, *m*/*z* 401.2 [M + H]^+^. ^1^H NMR (400 MHz, DMSO-*d*_*6*_) δ 9.28 (s, 1H), 8.61 (s, 1H), 8.14 (s, 1H), 7.98 (s, 1H), 7.89 (d, *J* = 1.0 Hz, 1H), 7.40 (s, 1H), 6.47 (s, 2H), 6.17 (d, *J* = 1.1 Hz, 1H), 3.67 (s, 3H), 3.46 (s, 3H), 2.55 (q, *J* = 7.6 Hz, 2H), 1.13 (t, *J* = 7.5 Hz, 3H).

#### 4-Methyl-5-((3-methyl-7-((5-morpholinopyridin-2-yl)amino)-3H-imidazo[4,5-*b*]pyridin-5-yl)oxy)picolinonitrile (**29**)

A mixture of 5-(7-chloro-3-methyl-3*H*-imidazo [4,5*-b*]pyridin-5-yloxy)-4-methyl-pyridine-2-carbonitrile **51b** (100 mg, 0.333 mmol, 1 equiv), 5-(morpholin-4-yl)pyridin-2-amine (72 mg, 0.400 mmol, 1.2 equiv), MorDalPhos Pd G4 (7 mg, 0.008 mmol, 0.024 equiv) and Cs_2_CO_3_ (269 mg, 0.833 mmol, 2.5 equiv) in anhydrous 1,4-dioxane (1.5 mL, 0.2 M) was degassed under nitrogen atmosphere and heated at 110 °C for 18 h. The mixture was filtered over Celite then concentrated. The crude was purified by preparatory HPLC (gradient from 30% MeCN to 55% MeCN in water/0.1% formic acid) to afford the title compound (61 mg, 41% yield). LCMS (ESI), Method A, *R*_t_ 1.16 min, purity >99%, *m*/*z* 443.2 [M + H]^+^. HRMS: Calculated mass for C_23_H_23_N_8_O_2_ (M+H)^+^ 443.19385; found 443.19259; difference 2.85 ppm. ^1^H NMR (400 MHz, DMSO-*d*_*6*_) δ 9.66 (s, 1H), 8.50 (s, 1H), 8.13 (s, 1H), 8.11 (s, 1H), 8.09 (s, 1H), 8.01 (d, *J* = 3.0 Hz, 1H), 7.45 (dd, *J* = 9.1, 3.1 Hz, 1H), 7.37 (d, *J* = 9.0 Hz, 1H), 3.78–3.72 (m, 4H), 3.59 (s, 3H), 3.12–3.05 (m, 4H), 2.28 (s, 3H). ^13^C NMR (101 MHz, DMSO-*d*_*6*_) δ 160.02, 153.17, 148.38, 144.66, 144.51, 143.49, 142.50, 141.52, 140.77, 134.18, 132.05, 127.69, 126.83, 122.05, 118.02, 114.32, 90.94, 66.47, 49.32, 29.81, 15.86. mp = 108.1–110.3 °C.

### General Procedure for the Preparation of **9**, **26**, **30**, and **31**

#### 4-Methyl-5-[3-methyl-7-(6-morpholin-4-yl-pyridazin-3-ylamino)-3H-imidazo[4,5-*b*]pyridin-5-yl]oxypyridine-2-carbonitrile (**9**)

A mixture of 5-(7-chloro-3-methyl-3*H*-imidazo [4,5*-b*]pyridin-5-yloxy)-4-methyl-pyridine-2-carbonitrile **51b** (500 mg, 1.672 mmol, 1 equiv), 6-(morpholin-4-yl)pyridazin-3-amine (301 mg, 1.672 mmol, 1 equiv), MorDalPhos (31 mg, 0.067 mmol, 0.04 equiv), [(Allyl)PdCl]_2_ (12 mg, 0.033 mmol, 0.02 equiv) and Cs_2_CO_3_ (654 mg, 2.006 mmol, 1.2 equiv) in anhydrous 1,4-dioxane (6 mL, 0.3 M) was degassed under nitrogen atmosphere and heated at 110 °C for 18 h. The mixture was filtered over Celite and concentrated. The resulting crude was purified by silica flash chromatography (DCM/MeOH, 98/2 to 95/5) and combined fractions were triturated with MeCN. The precipitate obtained was filtered off and dried to afford the title compound (250 mg, 34% yield). LCMS (ESI), Method B, *R*_t_ 1.13 min, purity >99%, *m*/*z* 444.2 [M + H]^+^. HRMS: Calculated mass for C_22_H_22_N_9_O_2_ (M+H)^+^ 444.1891; found 444.18862; difference 1.08 ppm. ^1^H NMR (400 MHz, DMSO-*d*_*6*_) δ 9.83 (s, 1H), 8.52 (s, 1H), 8.18 (s, 2H), 8.09 (s, 1H), 7.63 (d, *J* = 9.7 Hz, 1H), 7.41 (d, *J* = 9.7 Hz, 1H), 3.78–3.71 (m, 4H), 3.61 (s, 3H), 3.49–3.42 (m, 4H), 2.30 (s, 3H). ^13^C NMR (101 MHz, DMSO-*d*_*6*_) δ 159.89, 157.41, 153.16, 152.23, 144.57, 144.41, 142.83, 141.91, 140.62, 132.07, 127.66, 122.27, 121.67, 118.02, 117.43, 92.39, 66.32, 46.23, 29.88, 15.84. mp = 248.1–249.1 °C.

#### 4-Methyl-5-((3-methyl-7-((5-(morpholine-4-carbonyl)pyridin-2-yl)amino)-3H-imidazo[4,5-*b*]pyridin-5-yl)oxy)picolinonitrile (**26**)

This compound was prepared from **51b** and (6-aminopyridin-3-yl)(morpholino)methanone according to the general procedure used for the preparation of **9**. 65% yield. LCMS (ESI), Method A, *R*_t_ 1.11 min, purity >95%, *m*/*z* 471.2 [M + H]^+^. HRMS: Calculated mass for C_24_H_23_N_8_O_3_ (M+H)^+^ 471.18876; found 471.18953; difference 1.63 ppm. ^1^H NMR (400 MHz, DMSO-*d*_*6*_) δ 10.19 (s, 1H), 8.53 (s, 1H), 8.42 (d, *J* = 2.3 Hz, 1H), 8.29 (s, 1H), 8.21 (s, 1H), 8.11 (s, 1H), 7.79 (dd, *J* = 8.6, 2.4 Hz, 1H), 7.52 (d, *J* = 8.6 Hz, 1H), 3.66–3.59 (m, 7H), 3.58–3.50 (m, 4H), 2.28 (s, 3H). ^13^C NMR (101 MHz, DMSO-*d*_*6*_) δ 167.66, 159.91, 156.06, 152.99, 146.83, 144.90, 144.70, 142.48, 142.14, 140.99, 137.60, 132.10, 127.92, 124.24, 122.50, 118.00, 113.12, 92.81, 66.58, 29.87, 15.88. mp = 245.1–246.1 °C.

#### 4-Methyl-5-((3-methyl-7-((6-(4-methylpiperazin-1-yl)pyridazin-3-yl)amino)-3H-imidazo[4,5-*b*]pyridin-5-yl)oxy)picolinonitrile (**30**)

This compound was prepared from **51b** and 6-(4-methylpiperazin-1-yl)pyridazin-3-amine (**6c**) (Scheme S6, Supporting Information) according to the general procedure used for the preparation of **9**. 22% yield. LCMS (ESI), Method B, *R*_t_ 1.11 min, purity >99%, *m*/*z* 457.2 [M + H]^+^. HRMS: Calculated mass for C_23_H_25_N_10_O (M+H)^+^ 457.22073; found 457.2205; difference 0.51 ppm. ^1^H NMR (400 MHz, DMSO-*d*_*6*_) δ 9.82 (s, 1H), 8.53 (s, 1H), 8.19 (s, 1H), 8.18 (s, 1H), 8.11 (s, 1H), 7.60 (d, *J* = 9.7 Hz, 1H), 7.42 (d, *J* = 9.9 Hz, 1H), 3.61 (s, 3H), 3.52–3.46 (m, 4H), 2.47–2.41 (m, 4H), 2.30 (s, 3H), 2.23 (s, 3H).

#### 4-Methyl-5-((3-methyl-7-((6-((3*S*,5*R*)-3,4,5-trimethylpiperazin-1-yl)pyridazin-3-yl)amino)-3H-imidazo[4,5-*b*]pyridin-5-yl)oxy)picolinonitrile (**31**)

This compound was prepared from **51b** and 6-((3*R*,5*S*)-3,4,5-trimethylpiperazin-1-yl)pyridazin-3-amine **7c** (Scheme S7, Supporting Information) according to the general procedure used for the preparation of **9**. 1% yield. LCMS (ESI), Method B, *R*_t_ 1.21 min, purity >99%, *m*/*z* 485.2 [M + H]^+^. HRMS: Calculated mass for C_25_H_29_N_10_O (M+H)^+^ 485.25203; found 485.25211; difference 0.16 ppm. ^1^H NMR (400 MHz, DMSO-*d*_*6*_) δ 9.79 (s, 1H), 8.52 (s, 1H), 8.19 (s, 1H), 8.17 (s, 1H), 8.10 (s, 1H), 7.59 (d, *J* = 9.8 Hz, 1H), 7.42 (d, *J* = 9.8 Hz, 1H), 4.12–4.04 (m, 2H), 3.61 (s, 3H), 3.56–3.27 (m, 2H), 2.64–2.53 (m, 2H), 2.31 (s, 3H), 2.19 (s, 3H), 1.08 (d, *J* = 6.1 Hz, 6H). ^13^C NMR (101 MHz, DMSO-*d*_*6*_) δ 159.86, 156.78, 153.20, 151.72, 144.57, 144.34, 142.89, 141.89, 140.56, 132.10, 127.63, 122.26, 121.66, 118.02, 117.35, 92.33, 57.23, 52.60, 37.96, 29.88, 18.30, 15.84. mp = 108.4–109.4 °C.

### 6-((5-((6-Cyano-4-methylpyridin-3-yl)oxy)-3-methyl-3H-imidazo[4,5-*b*]pyridin-7-yl)amino)nicotinic acid (**54**)

#### Step 1: Methyl 6-((5-((6-cyano-4-methylpyridin-3-yl)oxy)-3-methyl-3H-imidazo[4,5-*b*]pyridin-7-yl)amino)nicotinate (**53**)

This compound was prepared from 5-(7-chloro-3-methyl-3*H*-imidazo [4,5*-b*]pyridin-5-yloxy)-4-methyl-pyridine-2-carbonitrile **51b** and methyl 6-aminopyridine-3-carboxylate **52** according to the general procedure used for the preparation of **9**. Crude material was purified by silica flash chromatography (DCM/MeOH, 97:3 to 94:6) to afford the title compound (7.30 g, 75% yield). LCMS (ESI) *m*/*z* 416.1 [M + H]^+^.

#### Step 2: 6-((5-((6-Cyano-4-methylpyridin-3-yl)oxy)-3-methyl-3H-imidazo[4,5-*b*]pyridin-7-yl)amino)nicotinic acid (**54**)

To a suspension of methyl 6-((5-((6-cyano-4-methylpyridin-3-yl)oxy)-3-methyl-3*H*-imidazo[4,5*-b*]pyridin-7-yl)amino)nicotinate **53** (7.30 g, 17.6 mmol, 1 equiv) in anhydrous pyridine (59 mL, 0.3 M) under N_2_ atmosphere was added LiI (7.07 g, 52.8 mmol, 3 equiv) and it was heated to 115 °C for 16 h. Additional LiI (3.00 g, 22.4 mmol, 1.3 equiv) was added and the reaction was heated to 115 °C for 16 h. The reaction mixture was cooled down to 0 °C and diluted with water. The pH of the aqueous phase was adjusted to 5–6 with a solution of 2 N HCl. The resulting precipitate was filtered and dried overnight *in vacuo* at 50 °C to afford the title compound (quantitative). LCMS (ESI) *m*/*z* 402.1 [M + H]^+^.

### General Procedure for the Preparation of **27** and **28**

#### 4-Methyl-5-((3-methyl-7-((5-(4-methylpiperazine-1-carbonyl)pyridin-2-yl)amino)-3H-imidazo[4,5-*b*]pyridin-5-yl)oxy)picolinonitrile (**27**)

A mixture of 6-((5-((6-cyano-4-methylpyridin-3-yl)oxy)-3-methyl-3*H*-imidazo[4,5*-b*]pyridin-7-yl)amino)nicotinic acid **54** (60 mg, 0.150 mmol, 1 equiv), 1-methylpiperazine (19 mg, 0.187 mmol, 1.25 equiv), *O*-(7-azabenzotriazol-1-yl)-*N,N,N′,N′*-tetramethyluronium-hexafluoro-phosphate (HATU; 71 mg, 0.187 mmol, 1.25 equiv) and Et_3_N (52 μL, 0.224 mmol, 1.5 equiv) in NMP (1.5 mL, 0.05 M) was stirred at rt for 16 h. The reaction mixture was purified as such by preparatory HPLC (gradient from 30% MeCN to 55% MeCN in water/0.5% NH_3_) to afford the title compound (2 mg, 3% yield). LCMS (ESI), Method B, *R*_t_ 0.88 min, purity >99%, *m*/*z* 484.3 [M + H]^+^. HRMS: Calculated mass for C_25_H_26_N_9_O_2_ (M+H)^+^ 484.2204; found 484.22129; difference 1.85 ppm. ^1^H NMR (400 MHz, DMSO-*d*_*6*_) δ 10.18 (s, 1H), 8.53 (s, 1H), 8.38 (d, *J* = 2.3 Hz, 1H), 8.28 (s, 1H), 8.20 (s, 1H), 8.10 (s, 1H), 7.76 (dd, *J* = 8.6, 2.4 Hz, 1H), 7.51 (d, *J* = 8.6 Hz, 1H), 3.60 (s, 3H), 3.59–3.45 (m, 4H), 2.37–2.30 (m, 4H), 2.28 (s, 3H), 2.20 (s, 3H). ^13^C NMR (101 MHz, DMSO-*d*_*6*_) δ 167.49, 159.91, 155.97, 153.00, 146.63, 144.88, 144.70, 142.50, 142.13, 140.98, 137.48, 132.09, 127.92, 124.62, 122.49, 117.99, 113.12, 92.79, 54.97, 46.07, 29.87, 15.88. mp = 193.1–194.1 °C.

#### rel-4-Methyl-5-((3-methyl-7-((5-((3aS,6aS)-1-methyloctahydropyrrolo[3,4-*b*]pyrrole-5-carbonyl)pyridin-2-yl)amino)-3H-imidazo[4,5-*b*]pyridin-5-yl)oxy)picolinonitrile (**28**)

This compound was prepared from **54** and *rel*-(3a*R*,6a*R*)-octahydro-1-methylpyrrolo[3,4-*b*]pyrrole according to the general procedure used for the preparation of **27**. 43% yield. LCMS (ESI), Method A, *R*_t_ 0.93 min, purity >99%, *m*/*z* 510.3 [M + H]^+^. HRMS: Calculated mass for C_27_H_28_N_9_O_2_ (M+H)^+^ 510.23605; found 510.23663; difference 1.14 ppm. ^1^H NMR (400 MHz, DMSO-*d*_*6*_) δ 10.18 (s, 1H), 8.54 (s, 1H), 8.52–8.47 (m, 1H), 8.30 (s, 1H), 8.21 (s, 1H), 8.11 (s, 1H), 7.86 (dd, *J* = 8.7, 2.4 Hz, 1H), 7.49 (d, *J* = 8.6 Hz, 1H), 3.60 (s, 6H), 2.99 (t, *J* = 8.4 Hz, 1H), 2.76 (q, *J* = 3.8 Hz, 2H), 2.34–2.10 (m, 8H), 1.95 (d, *J* = 35.9 Hz, 1H), 1.52 (s, 1H). mp = 118.1–119.1 °C.

### Ligand Docking

All calculations were carried out using Schrödinger software suite release 2017–3. All docked compounds were built and protonated using LigPrep software (Schrödinger, New York, USA), whereas ionization states at pH 7 ± 2 were calculated with Epik (Schrödinger, New York, USA).^60,61^

TYK2 (Protein Data Bank [PDB] code: 3LXN(62,63)) and JAK1 (PDB code: 3EYG(64,65)) structures were downloaded from the PDB.^66,67^ Hydrogen atoms were added to the protein through the Protein Preparation Wizard tool.^68^ In order to optimize the hydrogen bond network, the most probable protonation state of the residues was carefully selected by visual inspection and hydrogen atoms were minimized using OPLS3 force field.^69^ Before running the docking procedure all water molecules present in the structure were removed.

Docking of the ligands was carried out with Glide.^70−72^ Docking grids were generated using TYK2 and JAK1 prepared structures. The cocrystallized ligand was selected as the center of the grid, a hydrogen bond constraint with the hinge hydrogen-bond donor (Val981 NH for TYK2) was created, and the rest of the settings were kept as default. For the docking run, the flexible docking standard precision option was selected, together with an enhanced sampling protocol (four times) for the ligands. The constraint was applied to all docking runs, whereas the number of poses to return was set as five for each ligand.

The binding modes were then selected based on the spatial geometries of the ligand within the binding cavity, complementarity with the pocket (shape and electrostatic complementarity), hydrogen bond geometries of the protein–ligand interactions, and docking score.

### Molecular Dynamics Simulations

Molecular dynamics simulations were run using the Desmond package included in the Schrödinger suite. Ligand geometries were obtained from the docking studies and parametrized using OPLS3 force field. The complexes generated were initially solvated in a cubic box with SPC water molecules, leaving at least 10 Å between the solute atoms and the border of the box. The systems were then neutralized with seven Na^+^ counterions, and NaCl salt concentration of 0.15 M was added. The systems generated were equilibrated and gradually heated from 0 to 300 K using the Desmond default equilibration protocol. After the equilibration, the systems were subjected to 50 ns MD simulations in the NPT ensemble at 1.01325 bar (by Martyna–Tobias–Klein barostat) and 300 K (by Nosé–Hoover chain thermostat), setting a cutoff of 9 Å for the short-range nonbonded interactions. Trajectories were saved every 50 ps. Analysis of the simulation was based on protein and ligand RMSD values, hydrogen bond occupancy along 50 ns simulation, and visual inspection of the binding geometries.

### Quantum Mechanics Geometry Optimization

Geometry optimization of the docking poses of **10** and **11** in TYK2 was carried out with the Jaguar package^73^ included in the Schrödinger suite. B3LYP-D3/6-31G** functional and basis sets were used.^74−76^ All other parameters were kept to their default values.

### JAK1, JAK2, JAK3, and TYK2 Assays

The inhibitory effect of the described compounds against human TYK2 and its selectivity over JAK1, JAK2, and JAK3 was evaluated in the ADP-glo, radioactive assay kinase biochemical assays (Supporting Information; protocols).

### Cellular Assay, Mouse and Human Whole Blood Assays

These protocols can be found in the Supporting Information.

### Mouse Model of IL-23-Induced Psoriasis

Female BALB/c mice (Janvier Laboratories, France) were maintained in a specific pathogen-free animal facility (22 ± 2 °C) on a 12 h light/dark cycle (07:00–19:00). Food and filtered tap water were provided ad libitum. The study was performed according to the Animal Institutional Care and Use Committee of Galapagos. The animal care unit is authorized by the French “Ministère de l’Alimentation et de l’Agriculture” (Agreement No C93–063–06). Animal experiments were performed according to ethical guidelines of animal experimentation with respect to European Directive 2010. After a 7-day acclimatization period, the mice were randomly assigned to a treatment group (*n* = 10 per group), ensuring a homogeneous body weight distribution. On day 1, the left ears of the mice were shaved under anesthesia with a cocktail of ketamine and xylazine. Intradermal injections in the left ears of either 1 μg rmIL-23 (R&D system) in 20 μL of PBS/0.1% BSA or 20 μL of PBS/0.1% BSA alone were performed daily from day 1 through day 4. Compound **9** or TYK2 inhibitor used as positive reference^54^ were dosed orally once a day from day 1 through day 5. On day 4, blood sampling was performed for compound **9** treated mice at the retro-orbital sinus (under isoflurane anesthesia) at different time points and plasma was prepared to assess the concentration of circulating compound. The thickness of the left ear was measured daily before an intradermal injection of rmIL-23, using an electronic gage. On day 5, the mice were weighed and anesthetized by isoflurane inhalation. After measurement of ear thickness, mice were sacrificed by cervical elongation at *T*_max_ +1 h postdosing (120 min for TYK2 inhibitor and 75 min for **9**). The left ear was collected excluding cartilage: half ear was fixed in 4% formalin for pSTAT3 evaluation. The half ears were embedded in paraffin blocks and two different 4 μm thick sections were stained with anti-pSTAT3 antibody (Cell Signaling, #9145L) or with an antibody recognizing the neutrophil marker, 6A608, (Santa Cruz Biotechnology, reference sc-71674) by immunohistochemistry with the BOND-RX automate (Leica). The brown staining was quantified by image analysis (Calopix, Tribvn) as the immunopositive cell number per area of epidermis and dermis. Only STAT3 was analyzed from the STAT family as it is the main target of IL-23.

## Supporting Information Available

Additional figures and tables, description of *in vitro* assay systems, assay statistics, experimental procedures for compounds, analytical data for compounds (^1^H and ^13^C NMR spectra, and HPLC and LCMS conditions), table of compound SMILE data (PDF)
Molecular formula strings with associated data (CSV)

## Abbreviations Used

AUC: area under the curve
BINAP: 2,2′-bis(diphenylphosphino)-1,1′-binaphthyl
BrettPhos: 2-(dicyclohexylphosphino)3,6-dimethoxy-2′,4′,6′-triisopropyl-1,1′-biphenyl
CDT: 1,1′-carbonyl-di(1,2,4-triazole)
CL: clearance
CL_intu_: unbound intrinsic clearance
cLog D: calculated distribution coefficient
CL_u_: unbound clearance
CYP: cytochrome P450
ER: efflux ratio
ESI: electrospray ionization
EtOAc: ethyl acetate
F: bioavailability
FaSSGF: fasted state simulated gastric fluid
FaSSIF: fasted state simulated intestinal fluid
FeSSIF: fed state simulated intestinal fluid
fu: fraction unbound
GM-CSF: granulocyte macrophage colony-stimulating factor
HATU: O-(7-azabenzotriazol-1-yl)-*N*,*N*,*N′*,*N′*-tetramethyluronium-hexafluoro-phosphate
hERG: human Ether-à-go-go-Related Gene
HLM: human liver microsomes
HPLC: high-performance liquid chromatography
HRMS: high-resolution mass spectrometry
IFNα: interferon alpha
IL: interleukin
JAK: Janus kinase
LCMS: liquid chromatography–mass spectrometry
LipE: lipophilic efficiency
LipMetE: lipophilic metabolism efficiency
MD: molecular dynamics
MDCK-MDR1: Madin–Darby canine kidney cells with the multidrug resistance 1 gene (*ABCB1*)
MeCN: acetonitrile
MorDalPhos: di(1-adamantyl)-2-morpholinophenylphosphine
NMP: *N*-methyl-2-pyrrolidone
NT: not tested
OBD: optimum bed density
(p)IC_50_: (log10 or calculated logarithm) of IC_50_, half maximal inhibitory concentration
P_app_ A_2_B: apparent permeability from apical to basal chambers
Ppassive: passive permeability
PBMC: peripheral blood mononuclear cells
PDA: photo diode array
PE: petroleum ether
po: *per os* (oral administration)
(p)STAT: (phosphorylated) signal transducer and activator of transcription
q.d.: quaque die (once daily)
RuPhos: 2-dicyclohexylphosphino-2′,6′-diisopropoxybiphenyl
SLE: systemic lupus erythematosus
TDI: time-dependent inhibition
TMEDA: tetramethylethylenediamine
TYK2: tyrosine kinase 2
UHD: ultra high definition
UPLC: ultra high performance liquid chromatography
V_ss_: steady-state volume of distribution
Xantphos: 4,5-bis(diphenylphosphino)-9,9-dimethylxanthene
