# Supplementary material for: Discovery of GLPG3667, a Selective ATP Competitive Tyrosine Kinase 2 Inhibitor for the Treatment of Autoimmune Diseases
Source: J Med Chem. 2024 May 28;67(11):8545–68. doi: 10.1021/acs.jmedchem.4c00769 (PMC11181332; doi:10.1021/acs.jmedchem.4c00769)
Supplement: Supplementary file 1 — jm4c00769_si_001.pdf [file jm4c00769_si_001.pdf]

Supporting Information

Discovery of GLPG3667, a Selective ATP Competitive Tyrosine Kinase 2 Inhibitor for the Treatment of Autoimmune Diseases

Oscar Mammoliti,[a] Sébastien Martina,[a]* Pieter Claes,[a] Ghjuvanni Coti,[a] Roland Blanque,[b] Catherine Jagerschmidt,[b] Kenji Shoji,[b] Monica Borgonovi,[b] Steve De Vos,[a] Florence Marsais,[b] Line Oste,[a] Evelyne Quinton,[b] Miriam López-Ramos,[b] David Amantini,[b] Reginald Brys,[a] Juan-Miguel Jimenez,[a] René Galien[b] and Steven van der Plas[a]

[a]Galapagos NV, Generaal De Wittelaan L11, A3, 2800 Mechelen, Belgium

[b]Galapagos SASU, 102 Avenue Gaston Roussel, 93230 Romainville, France

*Corresponding author:

Sébastien Martina – Email: sebastien.martina@glpg.com

Table of Contents

Figure S1. Comparison between alkyl and aromatic substituents at the C5 position. S2

Figure S2. Kinome selectivity profile of compound **9**. S3

Table S1. Selectivity Profile of Compound **9** in the 21 Kinases with ≥50% Inhibition S4

Table S2. Chemical Stability of Compound **9** S5

Protocols for JAK1, JAK2, JAK3, TYK2, Cellular, Mouse Whole Blood, and Human Whole Blood Assays. S6

Experimental procedures for the intermediates **38**, **47**, **3b**, **4b**, **5e**, **6c,** and **7c**. S14

Analytical Data for **11**, **12**, **13**, **14**, **15**, **16**, **17**, **18**, **19**, **20**, **21**, **22**, **23**, **24**, **25**, **26**, **27**, **28**, **29**, **9**, **30,** and **31**. S20

Molecular Formula Strings List. S48

Compounds SMILE. S48

Reference S49

Figure S1. Comparison between alkyl and aromatic substituents at the C5 position. Alk, alkyl; Ar, aromatic; JAK, Janus kinase; TYK2, tyrosine kinase 2.

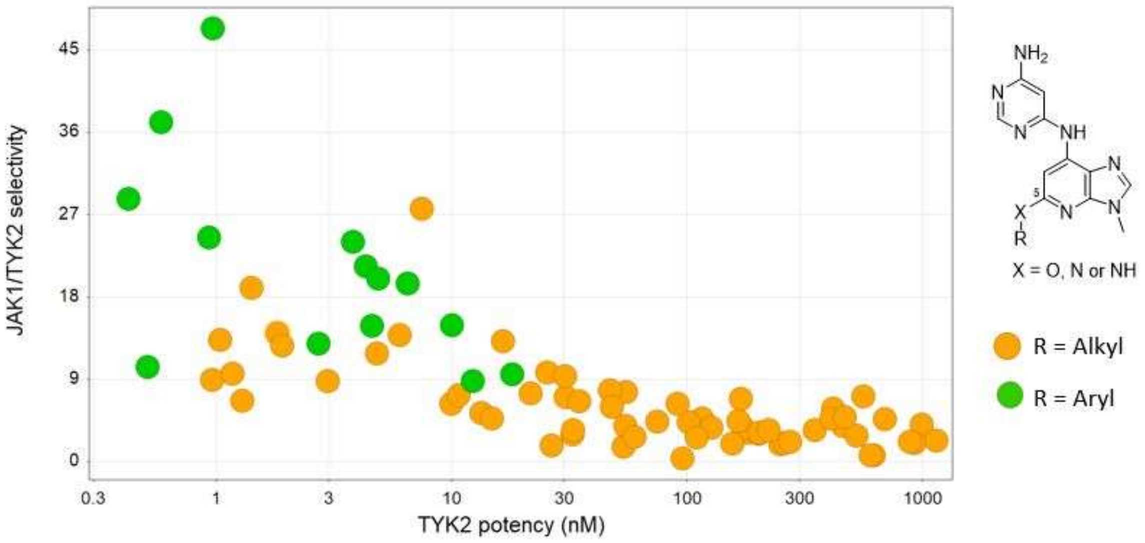

Figure S2. Kinome selectivity profile of compound **9**. Kinome-wide selectivity profiling of compound **9** with the KinaseProfile assay by Eurofins Discovery, Cerep, France. Measurements were performed at a concentration of 1 μM of the inhibitor in duplicate. The percent control means remaining active kinase percentage.

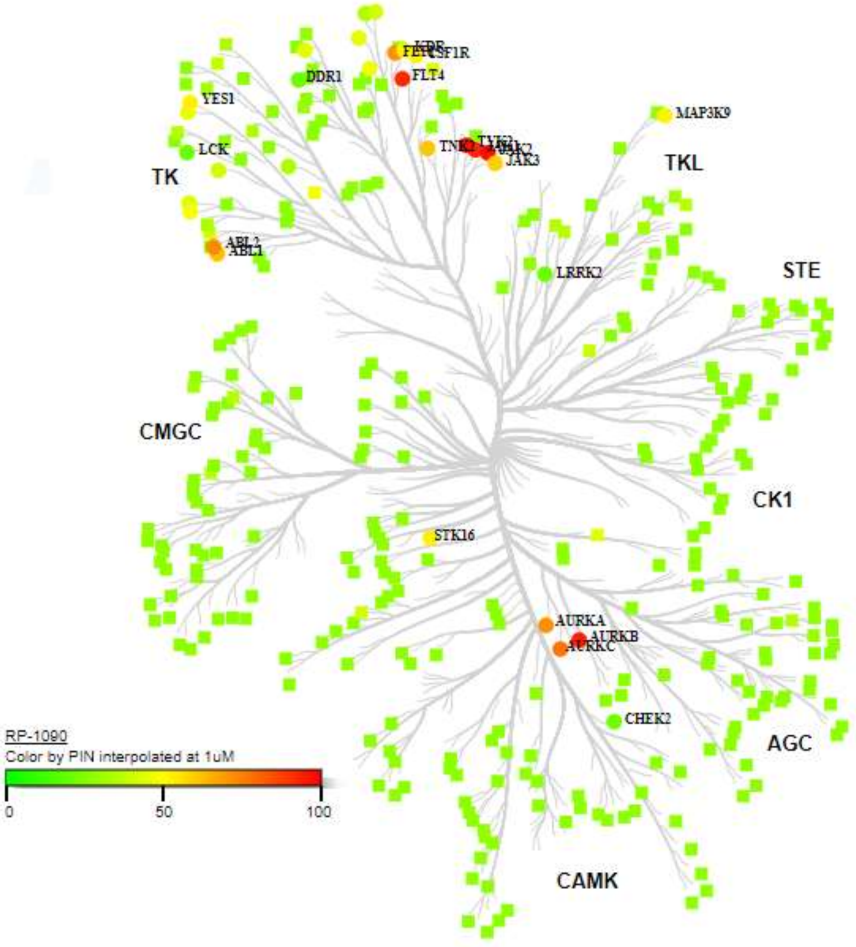

Table S1. Selectivity Profile of Compound **9** in the 21 Kinases with ≥50% Inhibition

kinase	single dose inhibition (%)[a]	biochemical IC_{50} (nM)[a]	cellular IC_{50} (nM)[b]	selectivity for tyrosine kinase 2
ABL1	92	324	NT	141
ABL2	73	179	NT	78
AURKA	73	202	NT	88
AURKB	93	21	2500	9
AURKC	73	142	NT	62
CSF1R	76	614	NT	267
DDR1	65	>10,000	NT	>4300
FGFR1	51	1474	NT	641
FGFR2	60	>3333	NT	>1400
FGFR3	55	>4350	NT	>1800
FLT1	85	202	NT	88
FLT3	91	44	3600	19
FLT4	95	39	NT	17
KDR	59	647	NT	281
LRRK2	55	>10,000	NT	>4300
MAP3K9	57	623	NT	271
NTRK1	52	>3924	NT	>1600
STK16	62	584	NT	254
TEC	50	>2286	NT	>1000
TNK2	70	336	NT	146
Yes	50	604	NT	263

[a]Experiments performed at Eurofins Discovery, Cerep, France using 1 µM against a panel of 365 kinases;

[b]experiments performed using ProQinase at Reaction Biology, Freiburg, Germany.

IC_{50}, half maximal inhibitory concentration; NT, not tested.

Table S2. Chemical Stability of Compound **9**

**pH**	**Percentage remaining (2 h)**[a]	**Percentage remaining (24 h)**[a]
1.2	100	111
5.0	94.0	108
7.4	102	115
9.0	96.7	103

[a]Stability of 2 µM of compound **9** in solution at various pH (pH 1.2, 5.0, 7.4, and 9.0) after incubation at 37°C for 2 or 24 h followed by LCMS measurement.

Protocols for JAK1, JAK2, JAK3, TYK2, Cellular, Mouse Whole Blood, and Human Whole Blood Assays.

JAK1 Inhibition Assay.

Recombinant human JAK1 (catalytic domain, amino acids 866–1154; catalog number PV4774) is purchased from Invitrogen. 1 ng of JAK1 (or 2 ng of JAK1 depending on the enzyme lot number) is incubated with 20 nM Ulight-JAK1 (tyr1023) peptide (PerkinElmer catalog number TRF0121) in kinase reaction buffer (25 mM MOPS, pH 6.8, 0.016% Brij-35, 8.33 mM MgCl2, 3.33 mM DTT, 20 μM ATP) with or without 4 μL containing test compound or vehicle (DMSO, 1% final concentration), in a total volume of 20 μL, in a white 384 Opti plate (PerkinElmer, catalog number 6007290). After 60 min at room temperature (rt), reactions are stopped by adding 20 μL/well of detection mixture (1 × detection buffer [PerkinElmer, catalog number CR97-100], 0.5 nM Europium-antiphosphotyrosine [PT66] [PerkinElmer, catalog number AD0068/AD0069], 10 mM EDTA). Readout is performed after 60 min incubation at rt using the Envision plate reader with excitation at 320 nm and measuring emission at 615 nm and 665 nm (PerkinElmer). The ratio of the relative fluorescence units (RFU) at 665 nm and 615 nm (RFU 665/RFU 615 multiplied by a factor of 1000) is used for further calculations. Kinase activity is calculated by subtracting the ratio obtained in the presence of a positive control inhibitor (1 μM staurosporine) from the ratio obtained in the presence of vehicle. The ability of a test compound to inhibit this activity (or percentage inhibition) is determined as:

$$\left(1-\frac{(Fluorescent\ ratio\ test\ compound-Fluorescent\ ratio\ control)}{(Fluorescent\ ratio\ vehicule-\ Fluorescent\ ratio\ control)}\right)\times 100$$

Wherein:

- Fluorescent ratio test compound = ratio RFU 665/RFU 615 × 1000 determined for sample with test compound present
- Fluorescent ratio control = ratio RFU 665/RFU 615 × 1000 determined for sample with positive control inhibitor

- Fluorescent ratio vehicle = ratio RFU 665/RFU 615 × 1000 determined in the presence of vehicle

Dose dilution series were prepared for the compounds enabling the testing of dose–response effects in the JAK1 assay and the calculation of the IC_{50} for the compound. Each compound is routinely tested at a concentration of 20 μM followed by a 1/5 serial dilution, 10 points, in a final concentration of 1% DMSO.

When the potency of the compound series increases, more dilutions are prepared and/or the top concentration is lowered (e.g., 5 μM, 1 μM). The data are expressed as the average IC_{50} from the assays.

JAK2 Inhibition Assay.

Recombinant human JAK2 (catalytic domain, amino acids 808–1132; catalog number PV4210) is purchased from Invitrogen. 0.83 ng of JAK2 is incubated with 25 nM Ulight-JAK1 (tyrl023) peptide (PerkinElmer catalog number TRF0121) in kinase reaction buffer (25 mM MOPS, pH 7.0, 0.01% Triton X-100, 7.5 mM $MgCL_2$, 2 mM DTT, and 0.3 μM ATP) with or without 4 μL containing test compound or vehicle (DMSO, 1 % final concentration), in a total volume of 20 μL, in a white 384 Opti plate (PerkinElmer, catalog number 6007290). After 60 min at rt, reactions are stopped by adding 20 μL/well of detection mixture (1 × detection buffer (PerkinElmer, catalog number CR97-100) with 0.5 nM Europium-antiphosphotyrosine (PT66) (PerkinElmer, catalog number AD0068/AD0069) and 10 mM EDTA). Readout is performed after 60 min incubation at rt using the Envision plate reader with excitation at 320 nm and measuring emission at 615 nm and 665 nm (PerkinElmer). The ratio of the RFU at 665 nm and 615 nm (RFU 665/RFU 615 multiplied by a factor of 1000) is used for further calculations. Kinase activity is calculated by subtracting the ratio obtained in the presence of a positive control inhibitor (1 μM staurosporine) from the ratio obtained in the presence of vehicle. The ability of a test compound to inhibit this activity (or percentage inhibition) is determined as:

$$\left(1 - \frac{(Fluorescent\ ratio\ test\ compound - Fluorescent\ ratio\ control)}{(Fluorescent\ ratio\ vehicule -\ Fluorescent\ ratio\ control)}\right) \times 100$$

Wherein:

- Fluorescent ratio test compound = ratio RFU 665/RFU 615 × 1000 determined for sample with test compound present
- Fluorescent ratio control = ratio RFU 665/RFU 615 × 1000 determined for sample with positive control inhibitor
- Fluorescent ratio vehicle = ratio RFU 665/RFU 615 × 1000 determined in the presence of vehicle

Dose dilution series are prepared for the compounds enabling the testing of dose–response effects in the JAK2 assay and the calculation of the IC_{50} for the compound. Each compound is routinely tested at a concentration of 20 μM followed by a 1/5 serial dilution, 10 points, in a final concentration of 1% DMSO. When the potency of the compound series increases, more dilutions are prepared and/or the top concentration is lowered (e.g., 5 μM, 1 μM). The data are expressed as the average IC_{50} from the assays.

JAK3 Radioactive Assay polyGT Substrate.

Recombinant human JAK3 catalytic domain (amino acids 795–1124; catalog number 08-046) is purchased from Carna Biosciences. 0.5 ng JAK3 protein is incubated with 2.5 pg polyGT substrate (Sigma catalog number P0275) in kinase reaction buffer (25 mM Tris, pH 7.5, 0.5 mM EGTA, 10 mM $MgCL_2$, 2.5 mM DTT, 0.5 mM Na_3V0_4, 5 mM b-glycerolphosphate, 0.01% Triton X-100, 1 μM nonradioactive ATP, 0.25 pCi 33P-gamma-ATP [PerkinElmer, catalog number NEG602K001MC] final concentrations) with or without 5 μL containing test compound or vehicle (DMSO, 1% final concentration), in a total volume of 25 μL in a polypropylene 96-well plate (Greiner, catalog number 651201). After 45 min at 30 °C, reactions are stopped by adding 25 μL/well of 150 mM phosphoric acid. All of the terminated kinase reaction is transferred to prewashed (75 mM phosphoric acid) 96-

well filter plates (PerkinElmer catalog number 6005177) using a cell harvester (PerkinElmer). Plates are washed six times with 300 μL per well of a 75 mM phosphoric acid solution and the bottom of the plates is sealed. 40 μL/well of Microscint-20 (PerkinElmer, catalog number 6013621) is added, the top of the plates is sealed, and readout is performed using the Topcount (PerkinElmer). Kinase activity is calculated by subtracting counts per min (cpm) obtained in the presence of a positive control inhibitor (10 μM staurosporine) from cpm obtained in the presence of vehicle.

The ability of a test compound to inhibit this activity (or percentage inhibition) is determined as:

$$\left(1 - \left(\frac{(cpm\ test\ compound - cpm\ control}{(cpm\ vehicle - cpm\ control}\right)\right) \times 100$$

Wherein:

- cpm test compound = cpm determined for sample with test compound present
- cpm control = cpm determined for sample with positive control inhibitor
- cpm vehicle = cpm determined in the presence of vehicle

Dose dilution series were prepared for the compounds enabling the testing of dose–response effects in the JAK3 assay and the calculation of the IC_{50} for each compound. Each compound is routinely tested at a concentration of 20 μM followed by a 1/5 serial dilution, 10 points, in a final concentration of 1% DMSO.

When the potency of the compound series increased, more dilutions were prepared and/or the top concentration was lowered (e.g., 5 μM, 1 μM).

TYK2 Radioactive Assay polyGT Substrate.

Recombinant human TYK2 catalytic domain (amino acids 871–1187; catalog number 08-147) is purchased from Carna Biosciences. 6 ng of TYK2 is incubated with 0.05 mg/mL polyGT substrate (Sigma catalog number P0275) in kinase reaction buffer (25 mM MOPS, pH 7.2, 50 mM NaCl, 0.5 mM EDTA, ImM DTT, 5 mM $MnCL_2$, 10 mM $MgCL_2$, 0.01% Brij-35, 0.1 μM nonradioactive

ATP, 0.125 pCi 33Pgamma-ATP [PerkinElmer, catalog number NEG602K00IMC], final concentrations) with or without 5 μL containing test compound or vehicle (DMSO, 1% final concentration), in a total volume of 25 μL, in a polypropylene 96-well plate (Greiner, catalog number 651201). After 120 min at 30 °C, reactions were stopped by adding 25 μL/well of 150 mM phosphoric acid. All of the terminated kinase reaction is transferred to prewashed (75 mM phosphoric acid) 96-well filter plates (PerkinElmer catalog number 6005177) using a cell harvester (PerkinElmer). Plates were washed six times with 300 μL per well of a 75 mM phosphoric acid solution and the bottom of the plates was sealed. 40 μL/well of Microscint-20 (PerkinElmer, catalog number 6013621) is added, the top of the plates is sealed, and readout is performed using the Topcount (PerkinElmer). Kinase activity is calculated by subtracting cpm obtained in the presence of a positive control inhibitor (10 μM staurosporine) from cpm obtained in the presence of vehicle. The ability of a test compound to inhibit this activity (or percentage inhibition) is determined as:

$$\left(1-\left(\frac{(cpm\ test\ compound-cpm\ control}{(cpm\ vehicle-cpm\ control}\right)\right)\times 100$$

Wherein:

cpm test compound = cpm determined for sample with test compound present

cpm control = cpm determined for sample with positive control inhibitor

cpm vehicle = cpm determined in the presence of vehicle

Dose dilution series were prepared for the compounds enabling the testing of dose–response effects in the TYK2 assay and the calculation of the IC_{50} for each compound. Each compound is routinely tested at a concentration of 20 μM followed by a 1/5 serial dilution, 10 points, in a final concentration of 1% DMSO.

When the potency of the compound series increased, more dilutions were prepared and/or the top concentration was lowered (e.g., 5 μM, 1 μM).

Cellular Assay.

Leucosep tubes were filled up with 15 mL of Ficoll and centrifuged to locate the Ficoll below the porous barrier. Blood from buffy coat was diluted three times in washing buffer (PBS), and 30 mL of diluted buffy is transferred into the prepared Leucosep tube. After centrifugation, the interface containing PBMCs was collected, washed twice in PBS, counted, and transferred into a cell culture flask at a cell density of 1 million cells/mL. Cells were then stimulated by the addition of 100 ng/mL of PMA and 0.5 μM of ionomycin in culture media (RPMI, 10% FBS, 1% antibiotics). The incubation was done for 4 days at 37 °C, 5% CO_2. On day 4, cells were left in starvation media (RPMI, 1% antibiotics) overnight.

For FACS studies, 0.5 million cells were dispensed into polypropylene 96-well round V bottom plates and incubated with G1256067 compound at different concentrations for 30 min at 37 °C under gentle agitation. After this incubation, cells were triggered with IFNα (1000 UI/mL) or vehicle (PBS + 0.1% BSA) for 30 min at 37 °C under gentle agitation. At the end of the incubation period, cells were fixed with 170 μl of prewarmed 1X lyse/fix buffer, and the samples then incubated for additional 10 min at 37 °C. After incubation, samples were centrifuged at 400 G for 5 min at rt and washed with PBS. Next, cells were resuspended in 300 μL of ice-cold 100% methanol to permeabilize the cells and immediately stored at −20 °C until further use. Before analysis, samples are centrifuged at 400 G for 5 min at 4 °C and supernatants were discarded and washed. The cell pellet was then resuspended in PBS (100 μL). 5 μl of anti-pSTAT1 couple to PE was added and incubated for 30 min at rt in the dark and washed once with PBS. Finally, tubes were analyzed on a FACS Lyric cytometer gating 10 000 events per sample.

The evaluation of phosphorylated STAT-3 and -4 was done using the Meso Scale Discovery (MSD) kit. Briefly, 0.2 million cells were dispensed into polypropylene 96-well round V bottom plates and incubated with G1256067 compound at different concentrations for 30 min at 37 °C under gentle agitation. After this incubation, cells were triggered with IL-23 (30 ng/mL), IL-12 (10 ng/mL), or IL-10 (10 ng/mL). Next, cells were centrifuged and supernatant was discarded. Then, 100 μL of cold

lysis buffer was added to the samples, incubated for 15 min on ice, and centrifuged at 4000 G for 10 min at 4 °C. Clarified lysate was transferred to a new polypropylene plate and stored at −80°C for further use. Detection of STAT phosphorylation was performed by transferring 25 μl of crude sample into a dedicated phospho STAT plate and reading out in a SQ120 QuickPlex

Mouse Whole Blood Assay.

Blood was collected from mice after decapitation, and added to tubes containing 0.5 M K3EDTA, which were gently inverted several times to prevent clotting and stabilized by incubating the tubes for 30 minutes at 37°C under gentle agitation on a bench rocker. 1 µL of compound **9** at different concentrations or vehicle (DMSO) were distributed into 2 mL tubes, then 50 µL of blood were added and incubated for 30 min at 37°C under gentle orbital agitation. After this incubation, blood was triggered with IL-6 (3 ng/mL), IFNα (6000 IU/mL) or vehicle (PBS + 0.1% BSA) at 37°C under gentle orbital agitation for 20 min or 60 min for IL-6 and for 60 min for IFNα. At the end of the incubation period, blood was transferred into FACS round-bottom tubes with cap and 1 mL of prewarmed 1X lyse/fix buffer was added, the tubes were then incubated for additional 10 minutes at 37°C in a water bath.

Samples were then centrifuged at 500 g for 5 min at room temperature and supernatant was discarded by inverting the tubes.

Triggering	Antibody	Fluorochrome	Volume/tube
IL-6	CD4	PerCp	2µL
	pSTAT1 (pY701)	PE	5µL
IFNα	CD4	PerCp	2µL
	pSTAT1 (pY701)	PE	5µL

Antibodies used in the mouse whole blood assays for the detection of pSTAT1 in CD4-positive cells

Human Whole Blood Assay.

Blood was collected from healthy volunteers into lithium heparin tubes by venipuncture, gently inverted several times to prevent clotting, and stabilized by incubating the tubes for 30 min at 37 °C under gentle agitation. Then, 100 μL of blood was dispensed into 2 mL microtubes and incubated with compound **9** at different concentrations for 30 min at 37 °C under gentle agitation. After this incubation, blood was triggered with either IL-2 (4 ng/mL), IL-6 (3 ng/mL), IFNα (1000 IU/mL), GM-CSF (20 pg/mL) or vehicle (PBS + 0.1% BSA) for 20 min at 37 °C under gentle agitation. At the end of the incubation period, blood was transferred into round-bottom tubes and 2 mL of prewarmed 1X lyse/fix buffer was added. The samples were incubated for an additional 10 min at 37 °C in a water bath, then centrifuged at 400 G for 8 min at rt.

The cell pellet was washed with PBS and resuspended in 100 μL of PBS. 900 μL of ice-cold 100% methanol was added drop by drop under gentle agitation to permeabilize the cells. Samples were then immediately stored at −20°C until further use. To perform the analysis, cells were washed once with PBS + 3% BSA and the supernatant was discarded. The cell pellet was resuspended in PBS + 3% BSA (100 μL). Anti-pSTAT1 and anti-CD4 antibodies were added to cell pellet triggered by IL-6 or IFNα, anti-pSTAT5 and anti-CD4 antibodies were added to cell pellet triggered by IL-2 and anti-pSTAT5, and anti-CD33 antibodies were added to cell pellet triggered by GM-CSF.

Samples were further incubated for 30 min at rt in the dark and then washed once with PBS. Finally, samples were measured on a FACS Canto II flow cytometer on 10 000 events and data analyzed after gating on $CD4^+$ cells (T cells) for pSTAT1 and pSTAT5, and on $CD33^+$ (monocytes) for pSTAT5.

Flow cytometry data were analyzed with the FACSDiva software and displayed as "percentage of pSTAT positive cells." Data were finally expressed as percentage inhibition. Graphs, pIC_{50}, and IC_{50} calculations were performed with the Prism 3.03 software (GraphPad). pIC_{50} and IC_{50} were calculated individually for each donor.

Experimental procedures for the intermediates 38, 47, 3b, 4b, 5e, 6c, and 7c.

Scheme S1[a]

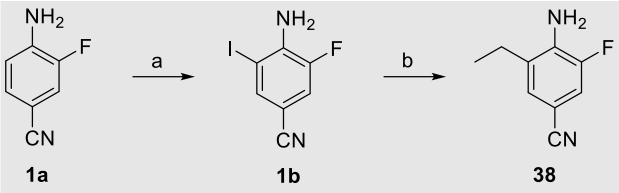

[a]Reagents and conditions: (a) I_2, Ag_2SO_4, EtOH, rt, 1 h; (b) Et_3B, Pd(dppf)Cl_2.CH_2Cl_2, Cs_2CO_3, DMF, 55 °C, 2 h.

Step 1: *4-Amino-3-ethyl-5-fluorobenzonitrile (**1b**)*. To a solution of iodine (18.6 g, 73.5 mmol, 1 equiv) in EtOH (367 mL, 0.2 M) was added Ag_2SO_4 (22.9 g, 73.5 mmol, 1 equiv) and 4-amino-3-fluorobenzonitrile **1a** (10 g, 73.5 mmol, 1 equiv). The mixture was stirred at rt for 1 h. After filtration, the mother liquor was concentrated under reduced pressure, diluted with DCM, and washed three times with saturated $Na_2S_2O_3$. The two layers were separated. The organic layer was washed with a saturated solution of ammonium chloride, dried over anhydrous Na_2SO_4, filtered, and concentrated under reduced pressure to afford the title compound (19.1 g, 99% yield). LCMS (ESI) m/z 279.8 [M + $NH_4]^+$.

Step 2: *4-Amino-3-ethyl-5-fluorobenzonitrile (**38**)*. To a mixture of 4-amino-3-fluoro-5-iodobenzonitrile **1b** (10 g, 38.2 mmol, 1 equiv) in DMF (190 mL, 0.2 M) was added triethylborane (1 M in dioxane, 50 mL, 49.6 mmol, 1.3 equiv), Cs_2CO_3 (74.6 g, 229 mmol, 6 equiv), and 1,1'-bis(diphenylphosphino)ferrocene-palladium(II)dichloride dichloromethane complex Pd(dppf)Cl_2.CH_2Cl_2 (2.79 g, 3.82 mmol, 0.1 equiv). The reaction mixture was degassed with nitrogen and heated at 100 °C for 2 h. After filtration, the mother liquor was diluted with DCM and washed with brine. The two layers were separated, and the organic layer was dried over anhydrous Na_2SO_4 and concentrated. The residue was purified by silica flash chromatography (PE/EtOAc 90:10 to 80:10) to afford the title compound (7.76 g, 64% yield). LCMS (ESI) m/z 164.9 $[M + H]^+$.

Scheme S2[a]

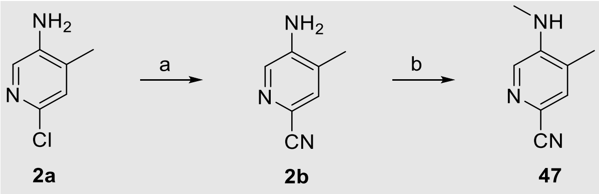

[a]Reagents and conditions: (a) $Zn(CN)_2$, $Pd(PPh_3)_4$, DMF, 150 °C; (b) LiHMDS, MeI, THF, −78 °C to rt.

Step 1: *5-Amino-4-methylpyridine-2-carbonitrile (**2b**)*. 5-amino-2-chloro-4-methyl-pyridine **2a** (850 mg, 5.98 mmol, 1 equiv), $Zn(CN)_2$ (770 mg, 6.58 mmol, 1.1 equiv) and $Pd(PPh_3)_4$ (692 mg, 0.59 mmol, 0.1 equiv) were mixed in anhydrous DMF (12 mL, 0.5 M) under N_2 and heated in a closed microwave tube at 150 °C for 5 min under microwave irradiations. The reaction mixture was cooled down to rt, poured into a saturated solution of $NaHCO_3$, and extracted with EtOAc. The organic phase was separated, dried over anhydrous Na_2SO_4, filtered, and concentrated. Crude material was purified by silica flash chromatography (petroleum ether/EtOAc: 80/20 to 0/100) to afford the title compound (302 mg, 38% yield). LCMS (ESI) m/z 133.9 $[M + H]^+$.

Step 2: *4-Methyl-5-(methylamino) pyridine-2-carbonitrile (**47**)*. A solution of 5-amino-4-methylpyridine-2-carbonitrile **2b** (1.0 g, 7.52 mmol, 1 equiv) in anhydrous THF (25 mL, 0.3 M) under N_2 was cooled to −78 °C. Next, LiHMDS (1 M in THF, 7.52 mL, 7.52 mmol, 1 equiv) was slowly added. The resulting mixture was stirred at rt for 3 h after which MeI (940 μL, 15.0 mmol, 2 equiv) was added dropwise. The mixture was stirred at rt for 18 h, then quenched with 1 mL of water and concentrated to dryness. Crude material was purified by silica flash chromatography (acetone/petroleum ether, 1:3) to afford the title compound (536 mg, 48% yield). LCMS (ESI) m/z 148.0 $[M + H]^+$.

Scheme S3[a]

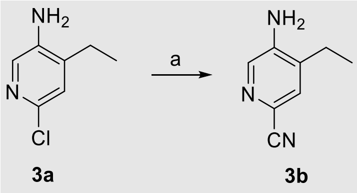

[a]Reagents and conditions: (a) $Zn(CN)_2$, $Pd(PPh_3)_4$, DMF, 150 °C.

*5-Amino-4-ethyl-pyridine-2-carbonitrile (**3b**).* 6-Bromo-4-ethylpyridin-3-amine **3a** (1.00 g, 5.00 mmol, 1 equiv), $Zn(CN)_2$ (644 mg, 5.50 mmol, 1.1 equiv) and $Pd(PPh_3)_4$ (578 mg, 0.50 mmol, 0.1 equiv) were mixed in anhydrous DMF (10 mL, 0.5 M) under N_2 and heated in a closed microwave tube at 150 °C for 6 min under microwave irradiations. The reaction mixture was cooled down to rt and poured in a saturated solution of $NaHCO_3$ and extracted with EtOAc. The organic phase was separated, dried over anhydrous Na_2SO_4, filtered, and concentrated. Crude material was triturated with MTBE. Solid was isolated by filtration and dried under reduced pressure to afford the title compound (397 mg, 54% yield). LCMS (ESI) m/z 148.0 $[M + H]^+$.

Scheme S4[a]

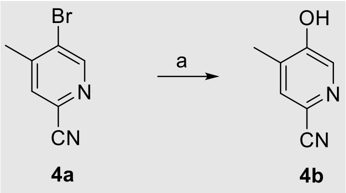

[a]Reagents and conditions: (a) *t*BuBrettPhos Pd G3, CsOH, water, 1,4-dioxane, rt, 2 h.

*5-hydroxy-4-methylpyridine-2-carbonitrile (**4b**).* A mixture of 5-bromo-2-cyano-4-methylpyridine **4a** (4.62 g, 23.4 mmol, 1 equiv), tBuBrettPhos Pd G3 (401 mg, 0.47 mmol, 0.02 equiv), $CsOH.H_2O$ (11.8 g, 70.3 mmol, 3 equiv) in 1,4-dioxane (48 mL, 0.5 M) and water (4.2 mL, 234 mmol, 10 equiv) was degassed with nitrogen and stirred at rt for 2 h. The reaction mixture was quenched with an aqueous solution of 2 M HCl and extracted with EtOAc. The two layers were separated. The organic layer was

washed with a saturated solution of $NaHCO_3$ and brine. The two layers were separated and the organic phase was dried over anhydrous Na_2SO_4, filtered, and concentrated under reduced pressure. Crude material was purified by silica flash chromatography (EtOAc/petroleum ether, 20:80 to 80:20) to afford the title compound (2.80 g, 67% yield). LCMS (ESI) m/z 134.9 $[M + H]^+$.

Scheme S5[a]

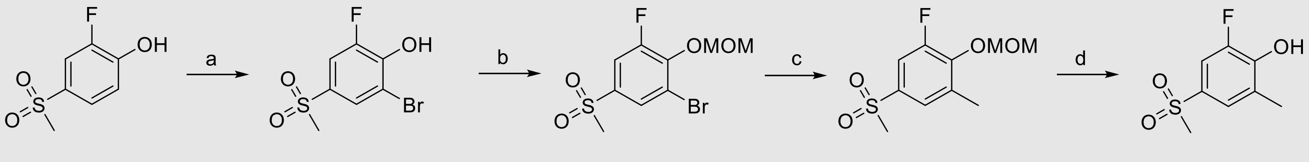

[a]Reagents and conditions: (a) Br_2, KOAc, AcOH, 0 °C, 30 min; (b) MOM-Cl, DIPEA, DCM, rt, 16 h; (c) methylboronic acid, $PdCl_2$(dppf), DCM, Cs_2CO_3, 1,4-dioxane, 100 °C, 2 h; (d) TFA, DCM, water, rt, 2 h.

Step 1: *2-Bromo-6-fluoro-4-methylsulfonyl-phenol (**5b**)*. A mixture of 2-fluoro-4-methylsulfonyl-phenol **5a** (3.00 g, 15.8 mmol, 1 equiv) and KOAc (1.55 g, 15.8 mmol, 1 equiv) in AcOH (45 mL, 0.35 M) was stirred at rt. The mixture was cooled to 0 °C and Br_2 (0.812 mL, 15.8 mmol, 1 equiv) was added dropwise. The mixture was stirred for 30 min at 0 °C, then concentrated. The precipitate obtained was filtered off, washed with water, and dried under vacuum to afford the title compound. LCMS (ESI) m/z 269.2 $[M + H]^+$.

Step 2: *1-Fluoro-2-(methoxymethoxy)-3-bromo-5-methylsulfonyl-benzene (**5c**)*. Chloromethyl methyl ether (0.87 mL, 11.45 mmol, 1.1 equiv) was dropwise added to the solution of 2-bromo-6-fluoro-4-methylsulfonyl-phenol **5b** (2.80 g, 10.4 mmol, 1 equiv) and DIPEA (3.63 mL, 20.8 mmol, 2 equiv) in DCM (21 mL, 0.5 M) at 0 °C. The reaction was stirred at rt overnight. Next, the reaction mixture was washed with water and the organic layer then washed with water, dried over anhydrous $MgSO_4$, filtered, and concentrated under reduced pressure to afford crude material. The resulting crude was purified by silica flash chromatography (PE/EtOAc, 0/100) to afford the title compound (2.80 g, 86%

yield, 74% purity). LCMS (ESI) m/z 222.1 $[M + H]^+$. ^{1}H NMR (300 MHz, DMSO-d_6) δ 8.02−7.98 (m, 1H), 7.91 (dd, J=10.7 Hz, J=2.1Hz, 1H), 5.31 (s, 2H), 3.52 (s, 3H), 3.30 (s, 3H).

Step 3: *1-Fluoro-2-(methoxymethoxy)-3-methyl-5-methylsulfonyl-benzene (**5d**)*. A suspension of l-fluoro-2-(methoxymethoxy)-3-bromo-5-methylsulfonyl-benzene **5c** (2.80 g, 8.94 mmol, 1 equiv) and Cs_2CO_3 (8.74 g, 26.82 mmol, 3 equiv) in anhydrous 1,4-dioxane (30 mL, 0.3 M) was degassed under nitrogen atmosphere. Pd(dppf)CL_2.DCM (1.638 g, 1.8 mmol, 0.2 equiv) and methylboronic acid (803 mg, 13.4 mmol, 1.5 equiv) were added to the reaction mixture and the reaction was sealed and stirred at 100 °C for 2 h. The reaction mixture was diluted with EtOAc and the solid was filtered off. The filtrate was concentrated under reduced pressure to afford crude material. The resulting crude was purified by silica flash chromatography (10% MeOH/DCM, 0/100 to 50/50) to afford the title compound (1.92 g, 86% yield). LCMS (ESI) m/z 249.3 $[M + H]^+$.

Step 4: *2-Fluoro-6-methyl-4-methylsulfonyl-phenol (**5e**)*. To a solution of 1-fluoro-2-(methoxymethoxy)-3-methyl-5-methylsulfonyl-benzene **5d** (1.90 g, 7.65 mmol, 1 equiv) in DCM (15 mL, 0.5 M) and TFA (11.7 mL, 153 mmol, 20 equiv) was added water (2 mL). The reaction was stirred at rt for 2 h and then concentrated under reduced pressure. The resulting crude product was dissolved in EtOAc and precipitated with cyclohexane. The precipitate obtained was filtered off and dried to afford the title compound (1.37 g, 89% yield). LCMS (ESI) m/z 205.3 $[M + H]^+$. ^{1}H NMR (300 MHz, DMSO-d_6) δ 7.55−7.50 (m, 2H), 5.74 (d, J=5.4 Hz, 1H), 3.01 (s, 3H), 2.33 (s, 3H).

Scheme S6[a]

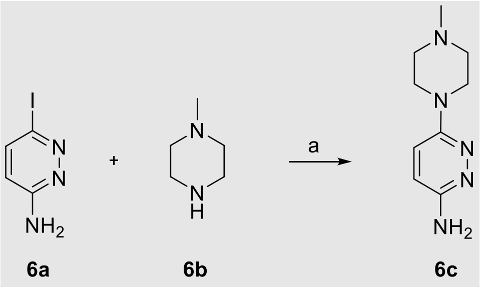

[a]Reagents and conditions: (a) CuI, L-hydroxyproline, K_3PO_4, DMSO, 60 °C, 48 h.

*6-(4-methylpiperazin-1-yl)pyridazin-3-amine (**6c**).* A mixture of 6-iodopyridazin-3-amine **6a** (2.00 g, 9.05 mmol, 1 equiv), CuI (172 mg, 0.905 mmol, 0.1 equiv), *L*-hydroxy proline (237 mg, 1.81 mmol, 0.2 equiv), K_3PO_4 (5.76 g, 27.15 mmol, 3 equiv) and 1-methyl piperazine **6b** (2 mL, 6.13 mmol, 4 equiv) in DMF (9 mL, 1 M) was heated at 60 °C for 48 h under nitrogen atmosphere. The reaction was quenched with MeOH and diluted with water. pH was adjusted to 7 using AcOH. The resulting precipitate was filtered off and washed with MeOH. The filtrate was passed through an SCX column (SCX-2, 0.6 mequiv/g, 30 g) with MeOH eluent, followed by a solution of 2 M NH_3 in MeOH to afford the title compound (343 mg, 20% yield). LCMS (ESI) m/z 194.1 $[M + H]^+$.

Scheme S7[a]

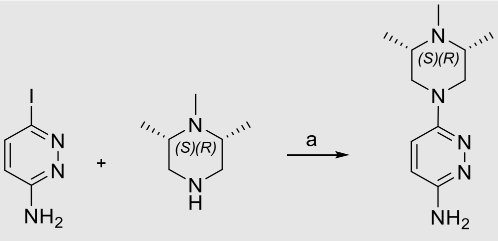

6a 7b 7c

[a]Reagents and conditions: (a) CuI, L-hydroxyproline, K_3PO_4, DMSO, 60 °C, 48 h.

*6-((3*R*,5*S*)-3,4,5-trimethylpiperazin-1-yl)pyridazin-3-amine (**7c**).* A mixture of 6-iodopyridazin-3-amine **6a** (171 mg, 0.774 mmol, 1 equiv), CuI (15 mg, 0.077 mmol, 0.1 equiv), *L*-hydroxy proline (20 mg, 0.156 mmol, 0.2 equiv), K_3PO_4 (493 mg, 2.325 mmol, 3 equiv) and (2*R*, 6*S*)-1,2,6-trimethylpiperazine **7b** (198 mg, 1.550 mmol, 2 equiv) in DMSO (2.5 mL, 0.3 M) was heated at 60 °C for 48 h under nitrogen atmosphere. DMSO was removed under reduced pressure. Residue was diluted with MeOH filtered over Pall Seitz filter paper and the filtrate was concentrated. The resulting crude was purified by silica flash chromatography (7 N NH_3 in MeOH/DCM, 0.5/99.5 to 10/90) to afford the title compound (121 mg, 71% yield). LCMS (ESI) m/z 222.1 $[M + H]^+$.

Analytical Data for 11, 12, 13, 14, 15, 16, 17, 18, 19, 20, 21, 22, 23, 24, 25, 26, 27, 28, 29, 9, 30, and 31.

Analytical Data for 11.

Chromatogram of compound **11**

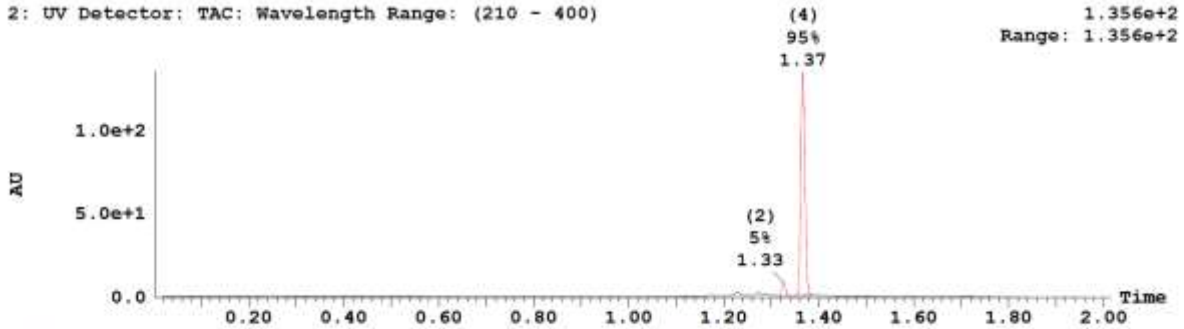

Peak Number	Compound	Time	AreaAbs	Area %Total	Width	Height	Mass Found
2		1.33	7e+004	5.14	0	8e+006	Not Found
4		1.37	1e+006	94.86	0	1e+008	Not Found

Mass spectrum of compound **11**

Peak ID	Compound	Time	Mass Found
4		1.37	Not Found

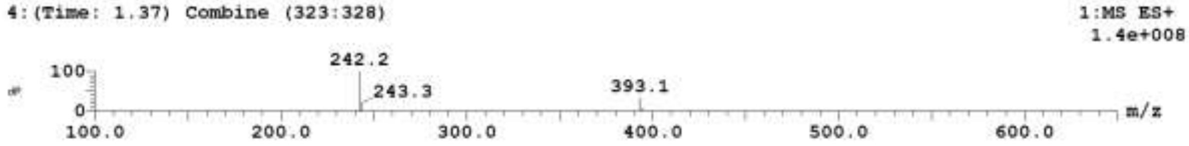

Analytical Data for 12.

^{1}H NMR of compound **12**

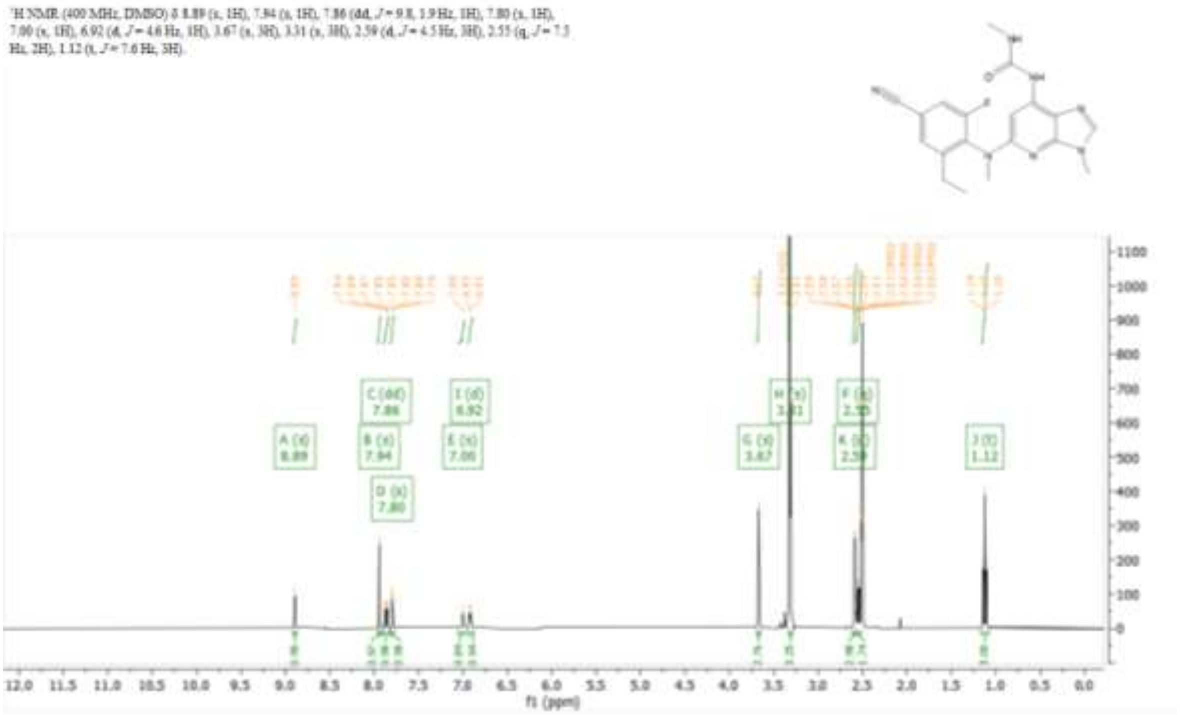

Chromatogram of compound **12**

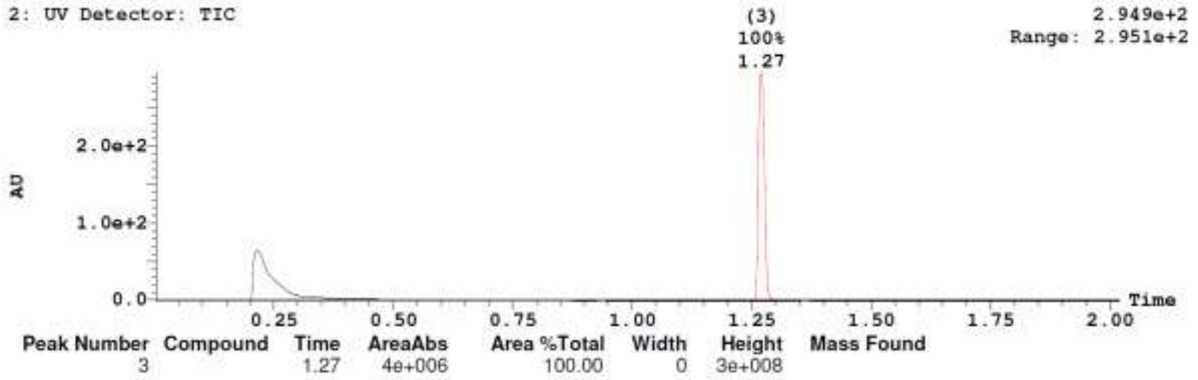

Mass spectrum of compound **12**

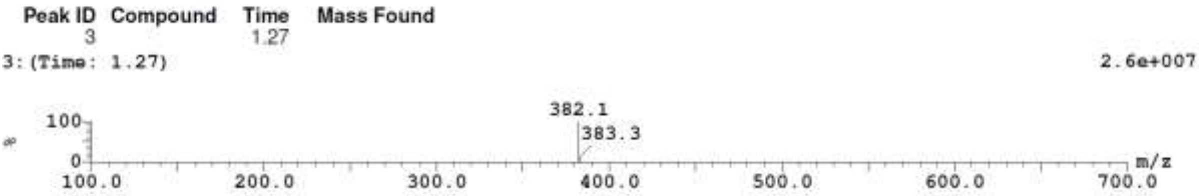

Analytical Data for 13.

^{1}H NMR of compound **13**

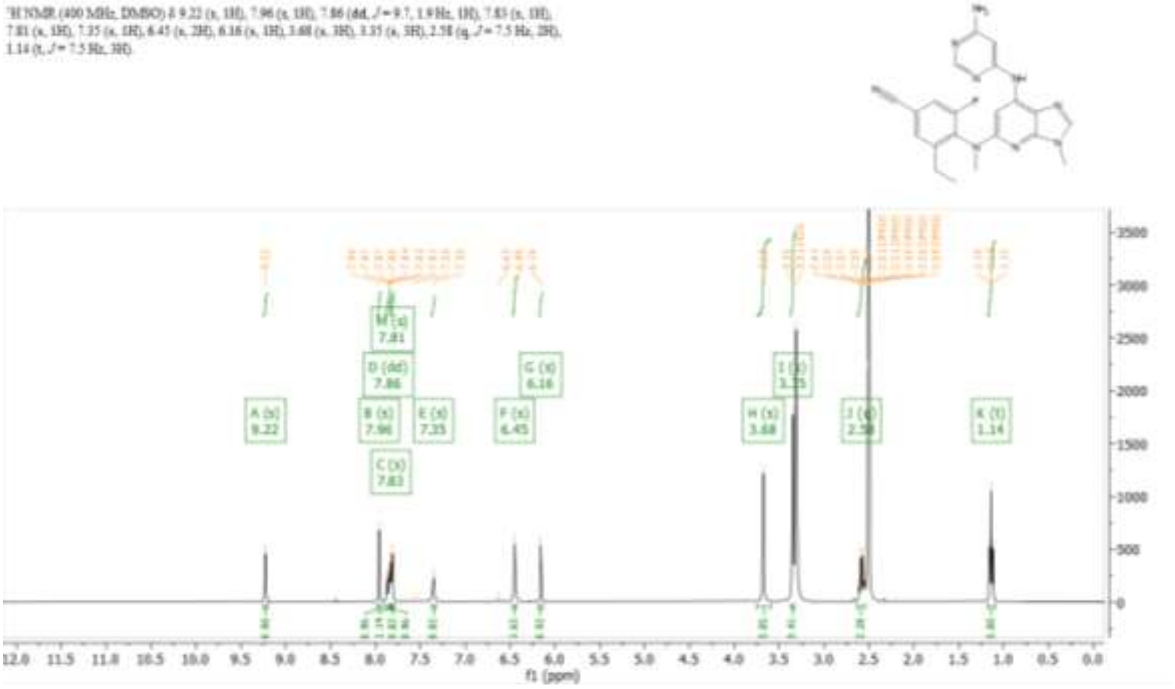

Chromatogram of compound **13**

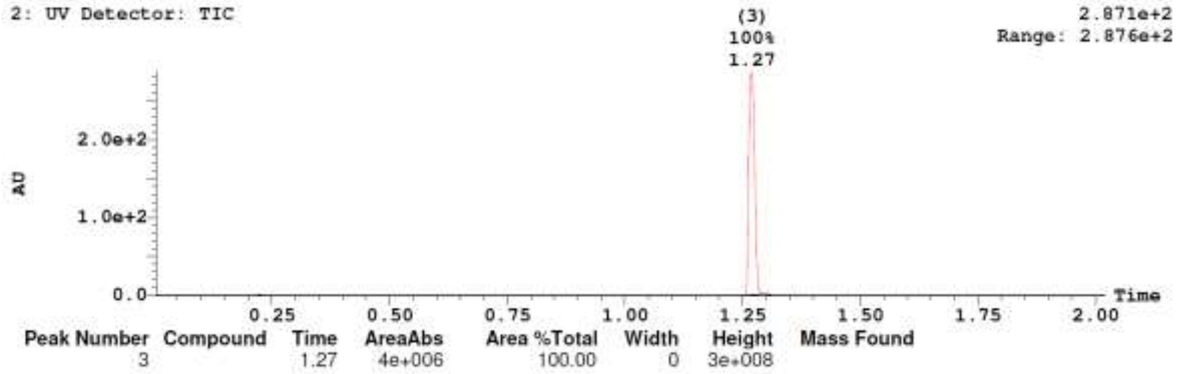

Mass spectrum of compound **13**

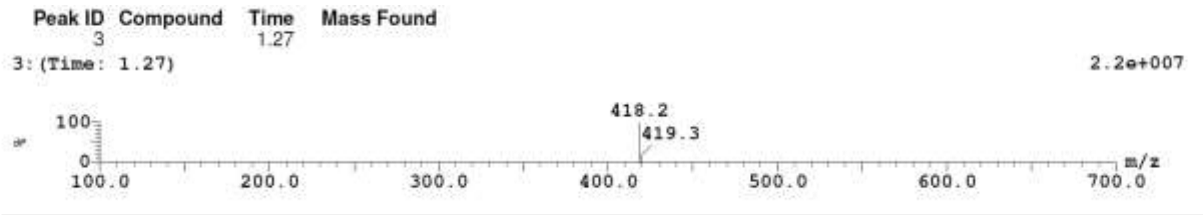

Analytical Data for 14.

^{1}H NMR of compound **14**

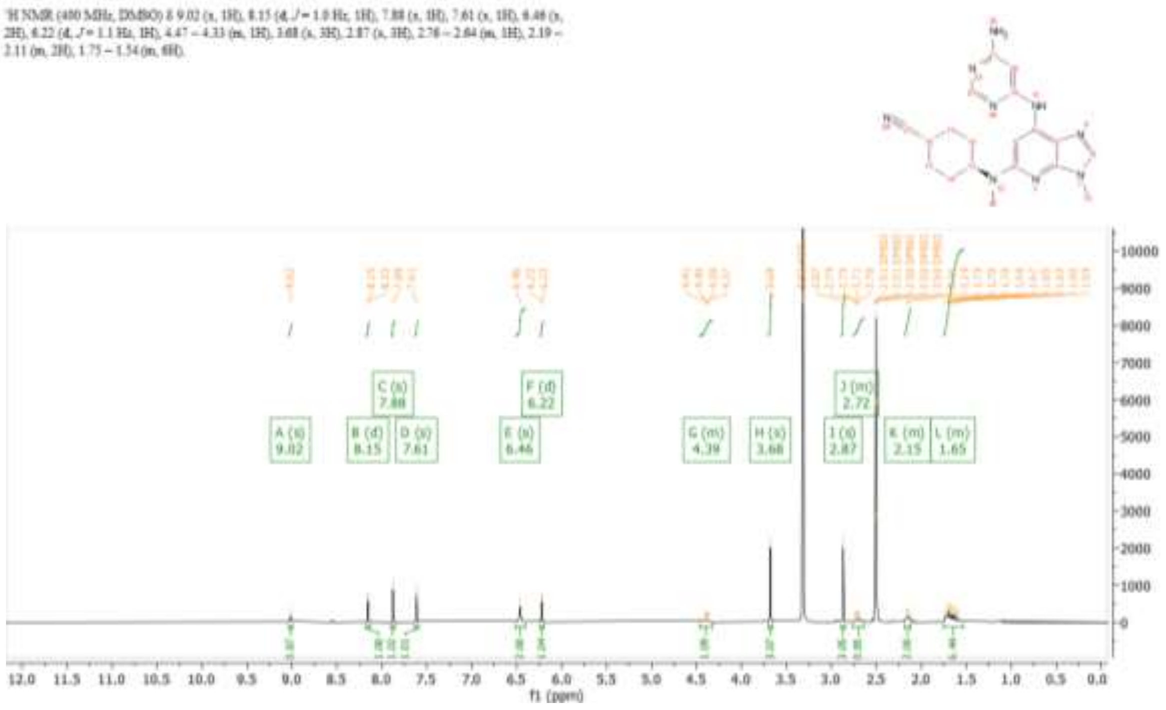

Chromatogram of compound **14**

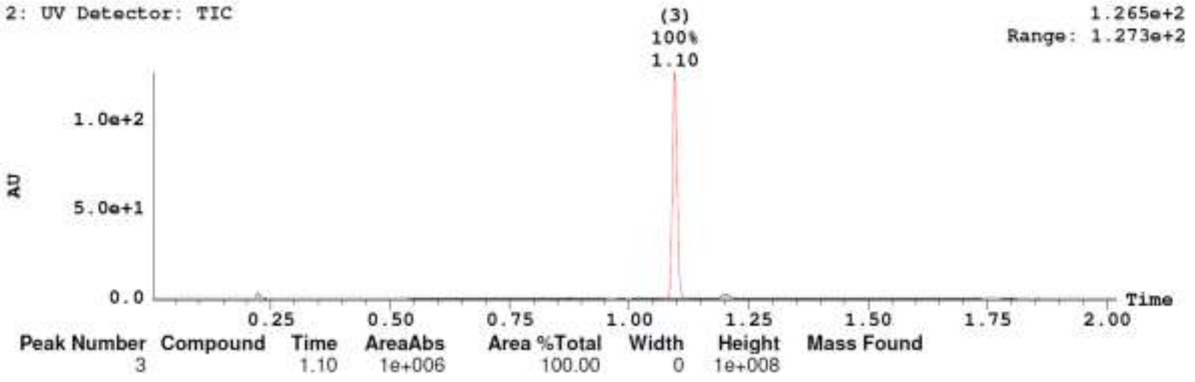

Mass spectrum of compound **14**

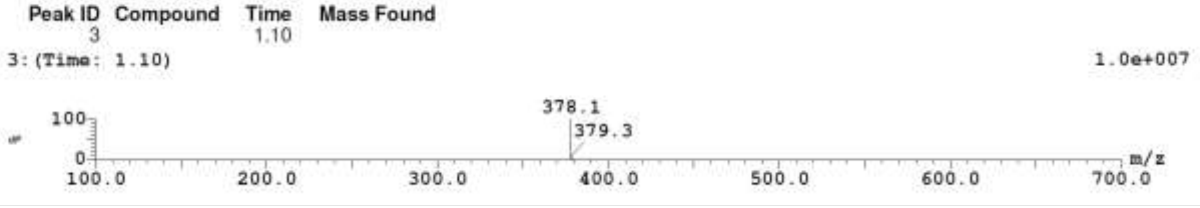

Analytical Data for 15.

^{1}H NMR of compound **15**

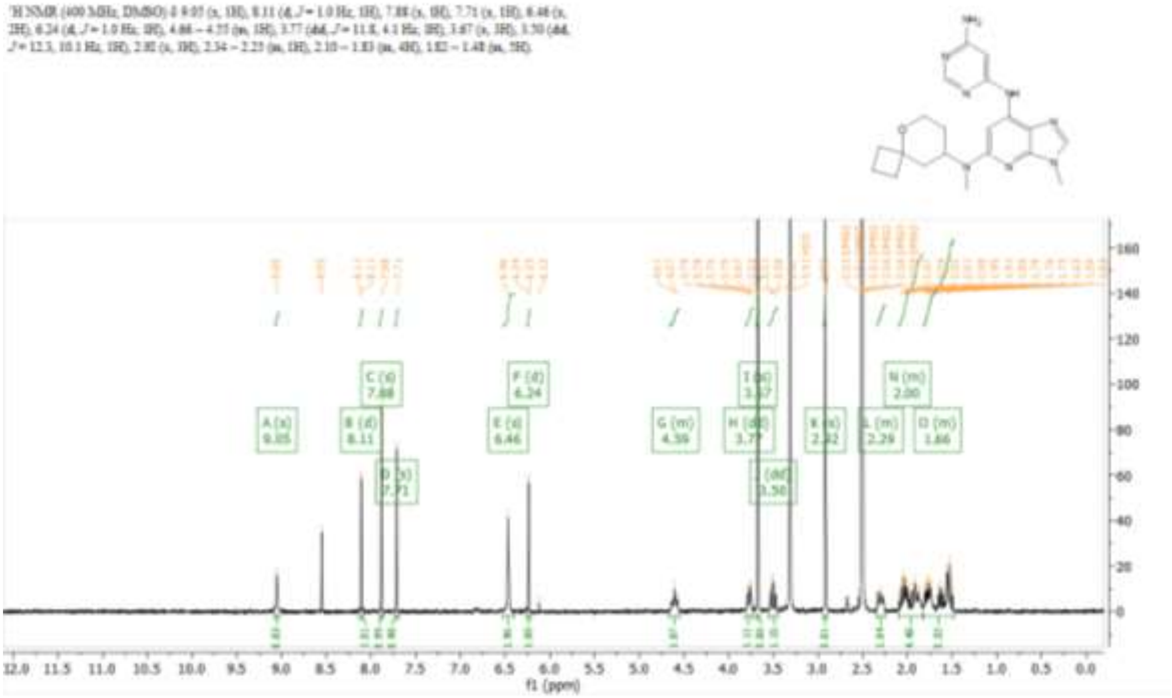

Chromatogram of compound **15**

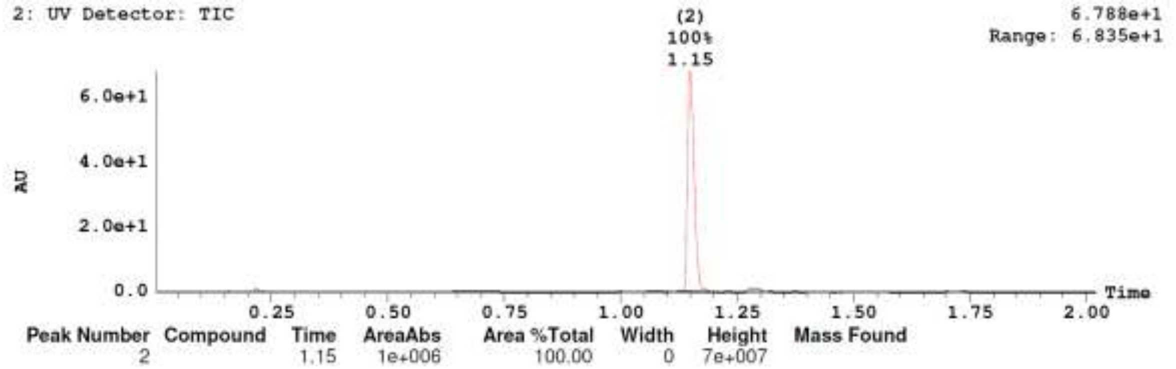

Mass spectrum of compound **15**

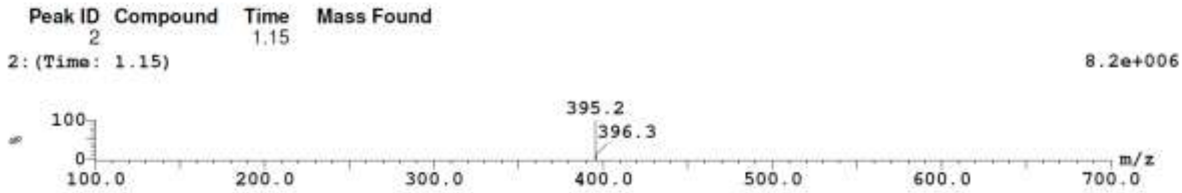

Analytical Data for 16.

^{1}H NMR of compound **16**

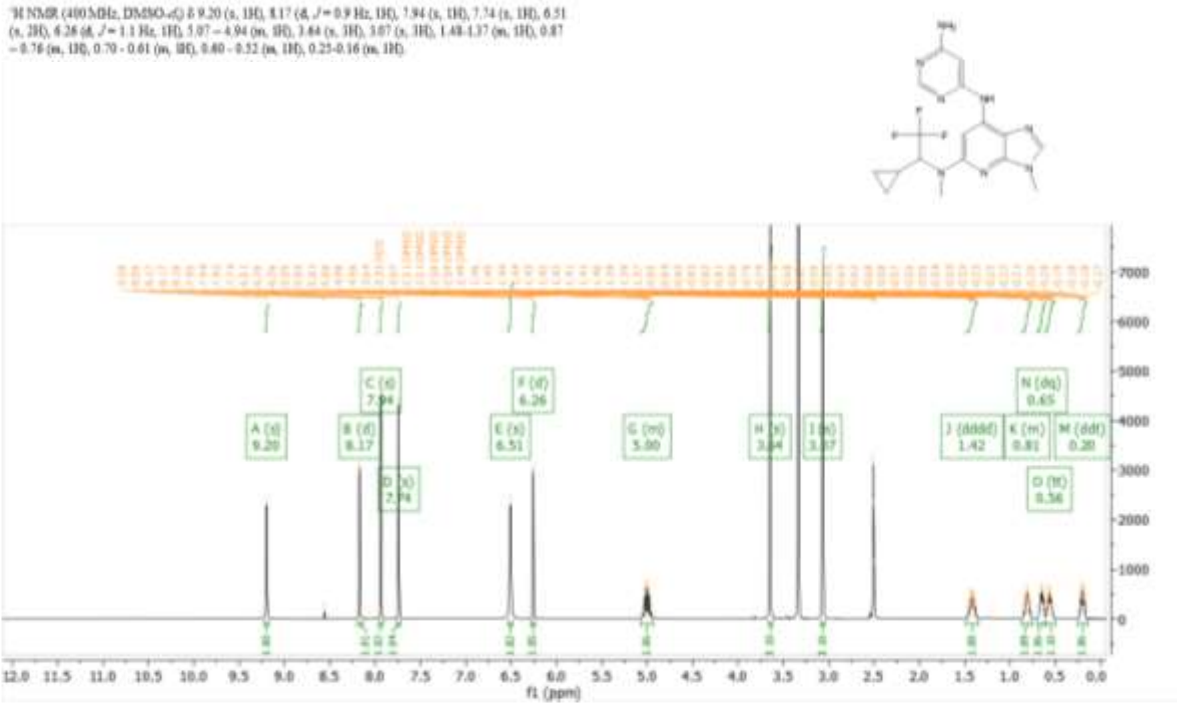

^{13}C NMR of compound **16**

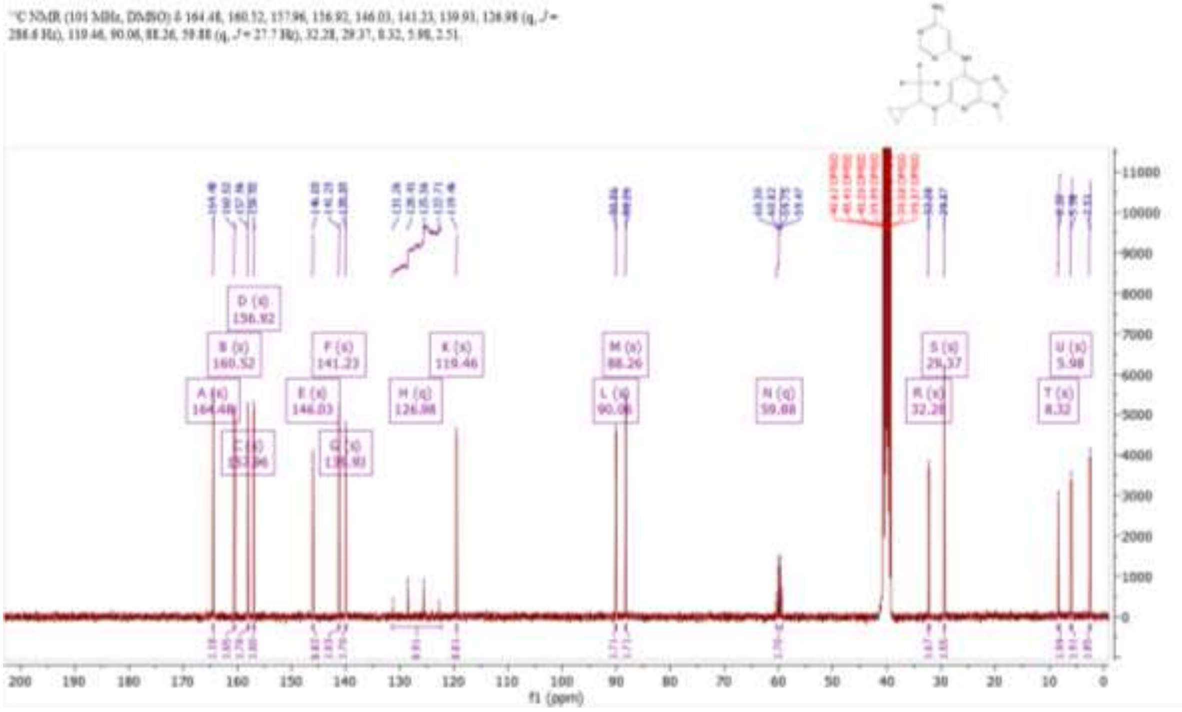

Chromatogram of compound **16**

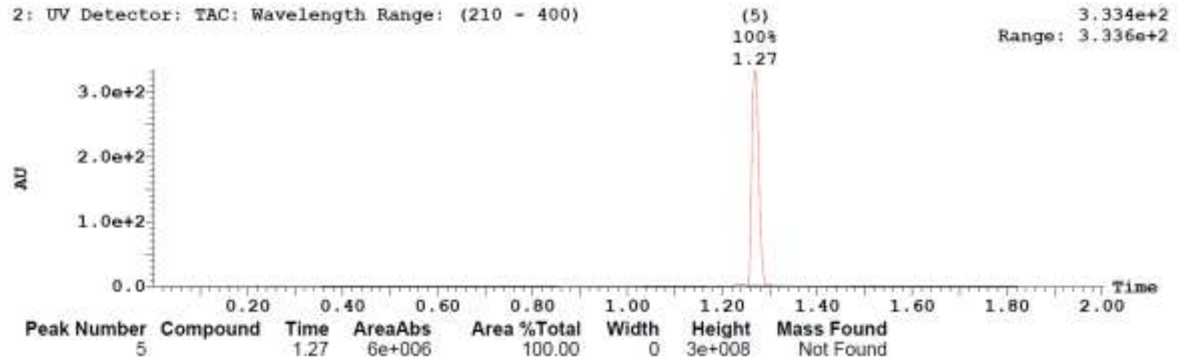

Mass spectrum of compound **16**

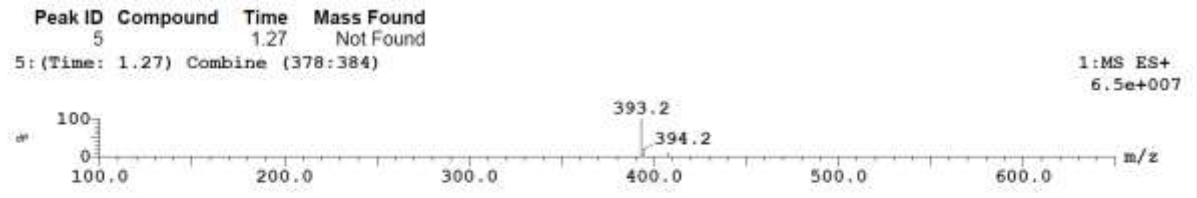

Analytical Data for 17.

^{1}H NMR of compound **17**

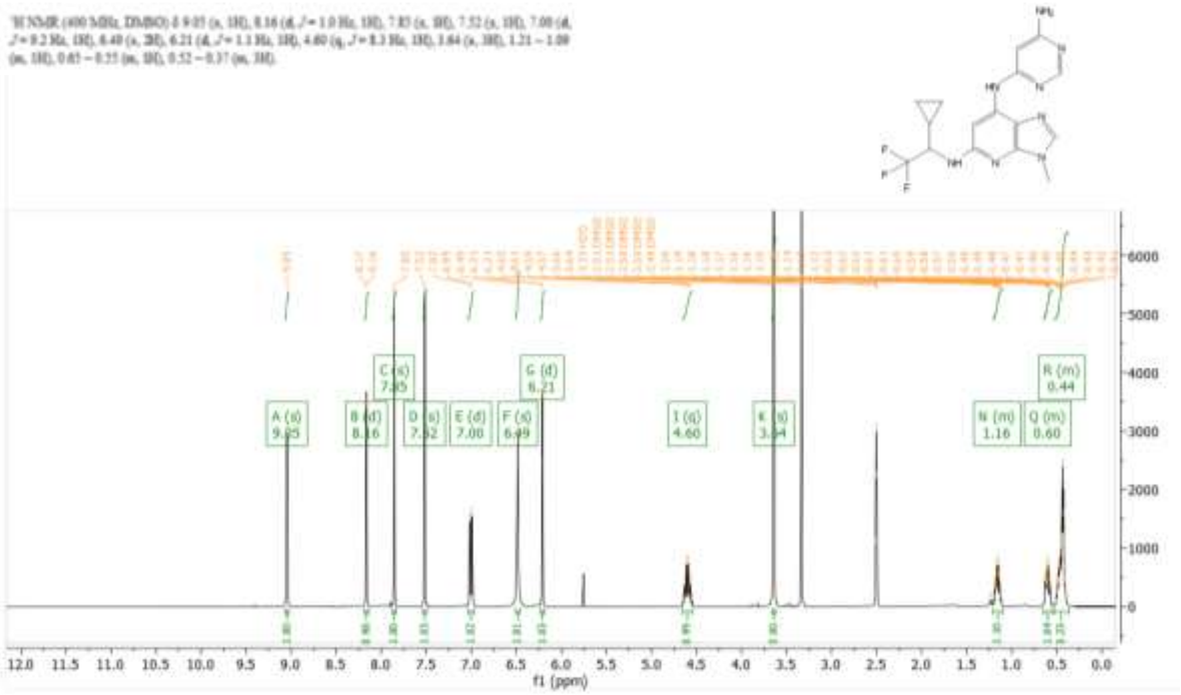

^{13}C NMR of compound **17**

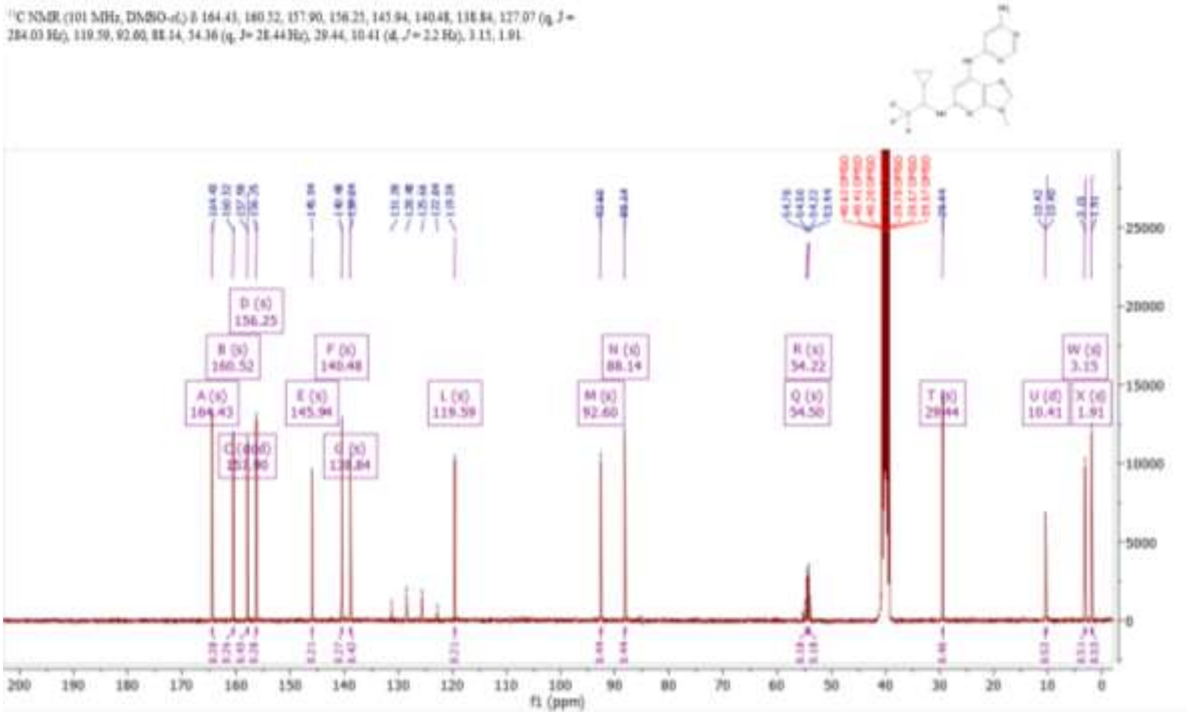

Chromatogram of compound **17**

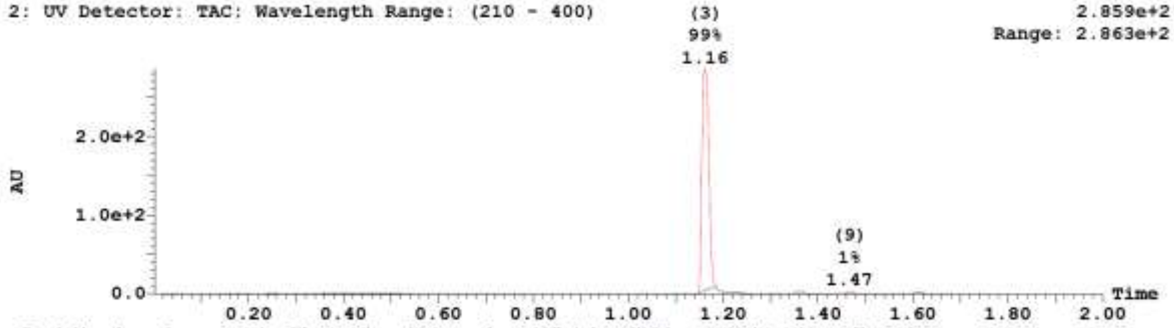

Peak Number	Compound	Time	AreaAbs	Area %Total	Width	Height	Mass Found
3		1.16	5e+006	98.91	0	3e+008	Not Found
9		1.47	5e+004	1.09	0	3e+006	Not Found

Mass spectrum of compound **17**

Peak ID	Compound	Time	Mass Found
3		1.16	Not Found

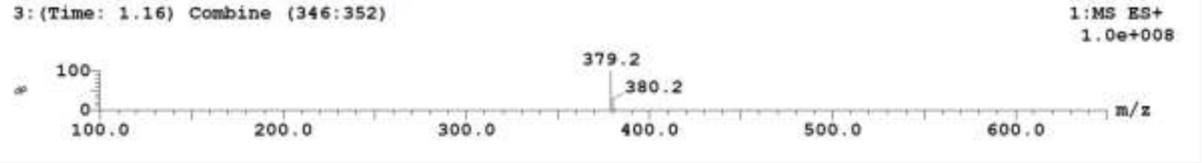

Analytical Data for 18.

^{1}H NMR of compound **18**

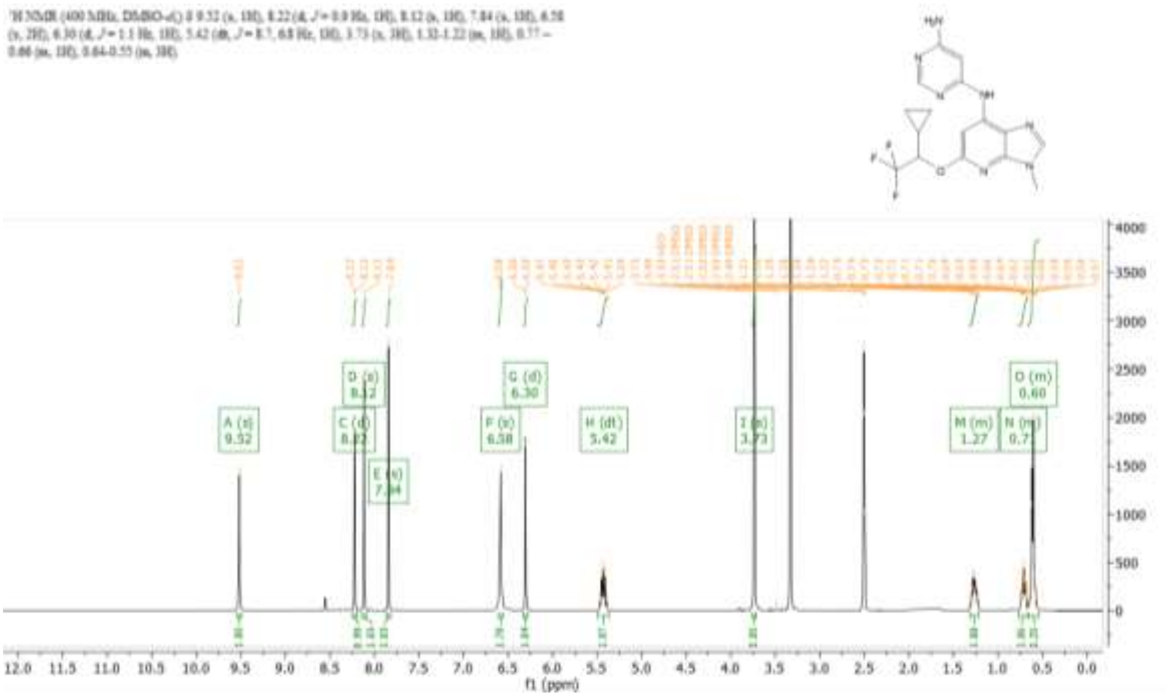

^{13}C NMR of compound **18**

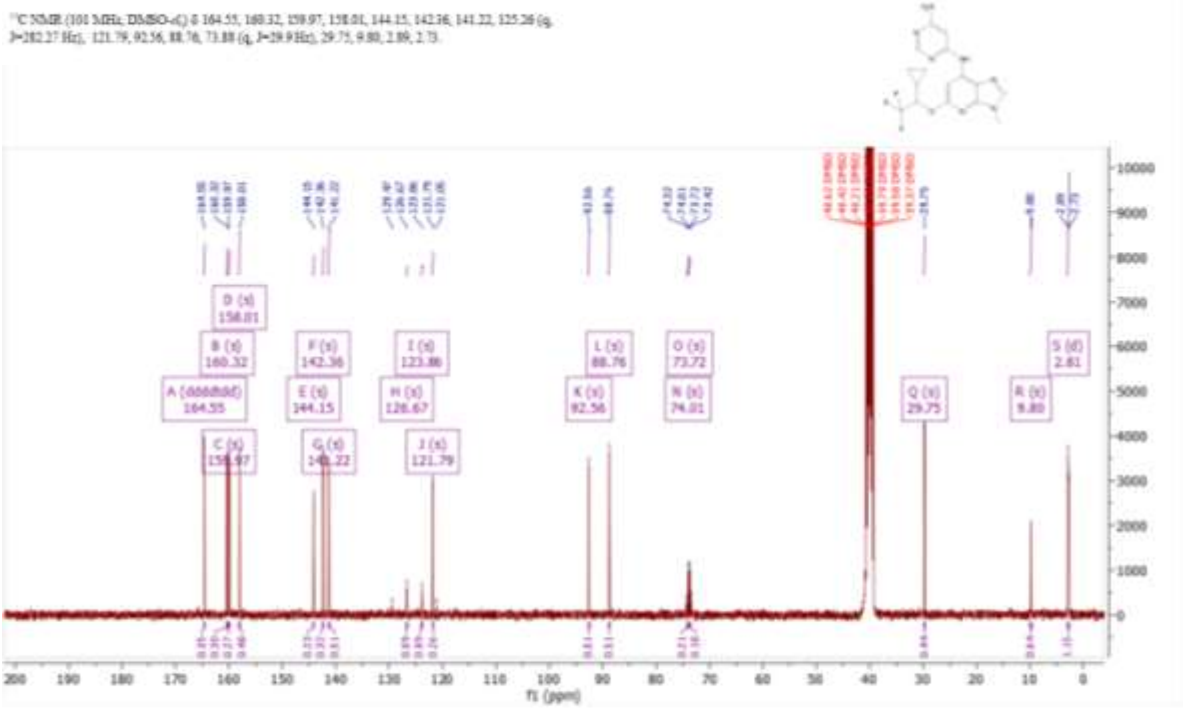

Chromatogram of compound **18**

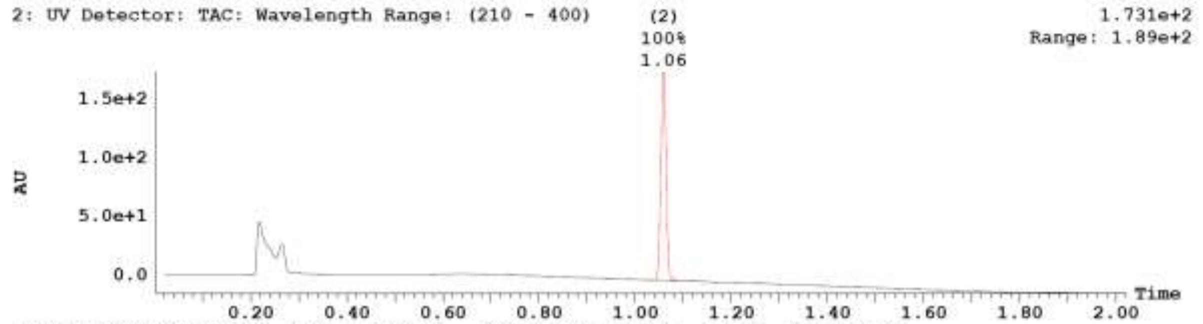

Peak Number	Compound	Time	AreaAbs	Area %Total	Width	Height	Mass Found
2		1.06	2e+006	100.00	0	2e+008	Not Found

Mass spectrum of compound **18**

Peak ID	Compound	Time	Mass Found
2		1.06	Not Found

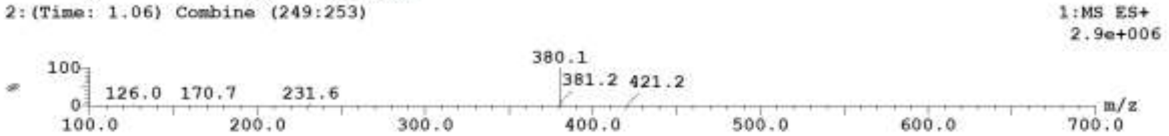

Analytical Data for 19.

^{1}H NMR of compound **19**

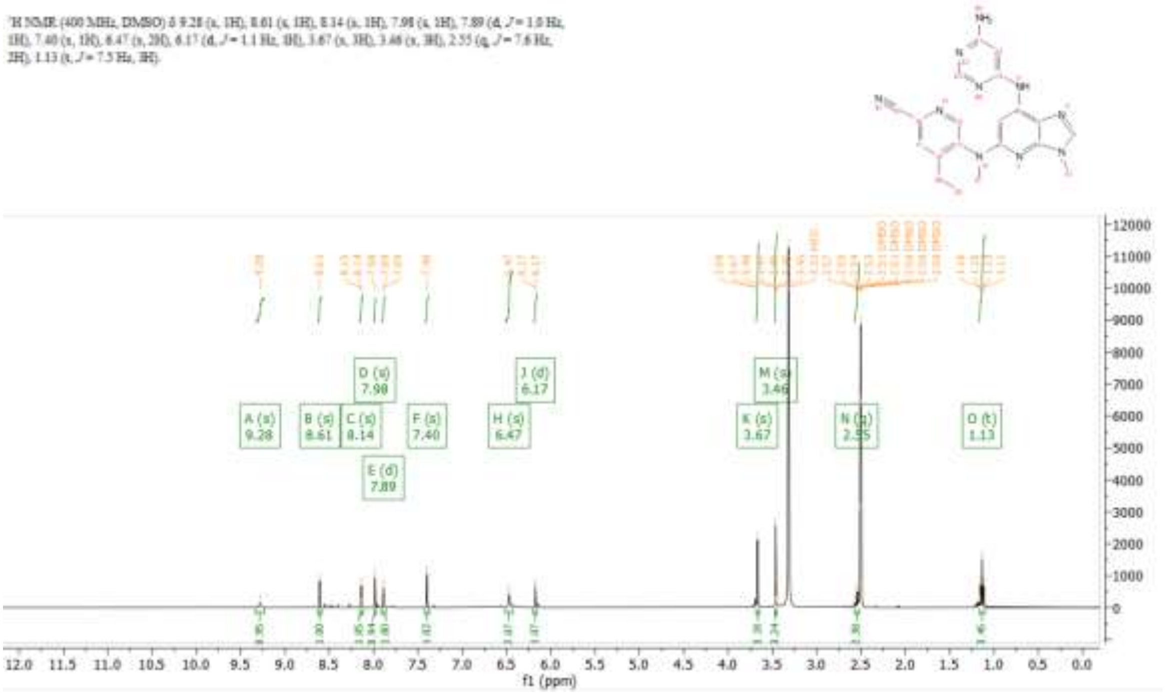

Chromatogram of compound **19**

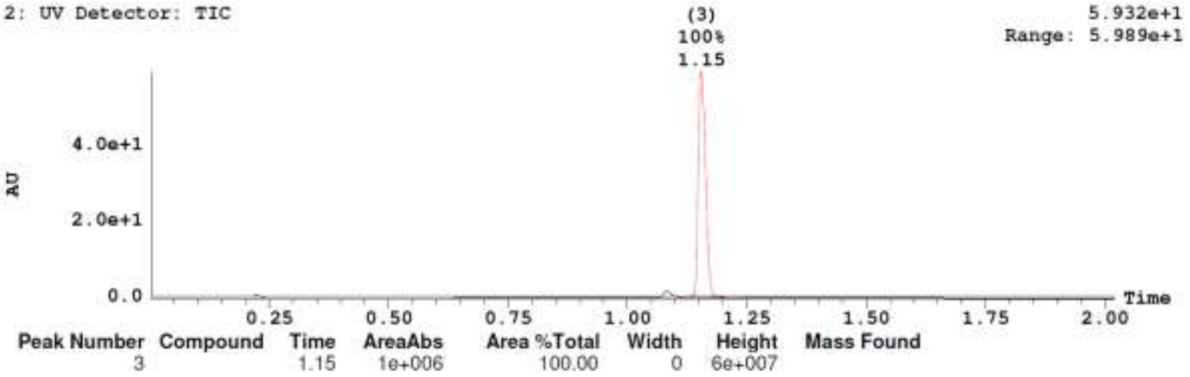

Mass spectrum of compound **19**

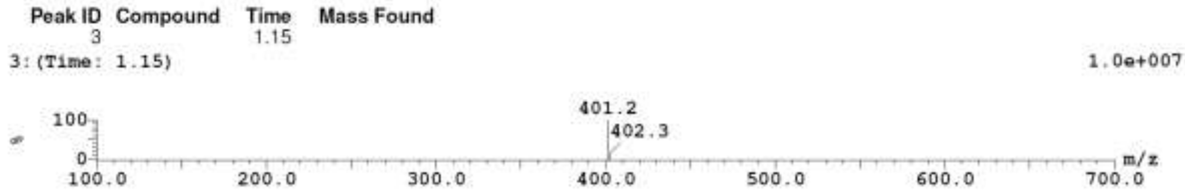

Analytical Data for 20.

^{1}H NMR of compound **20**

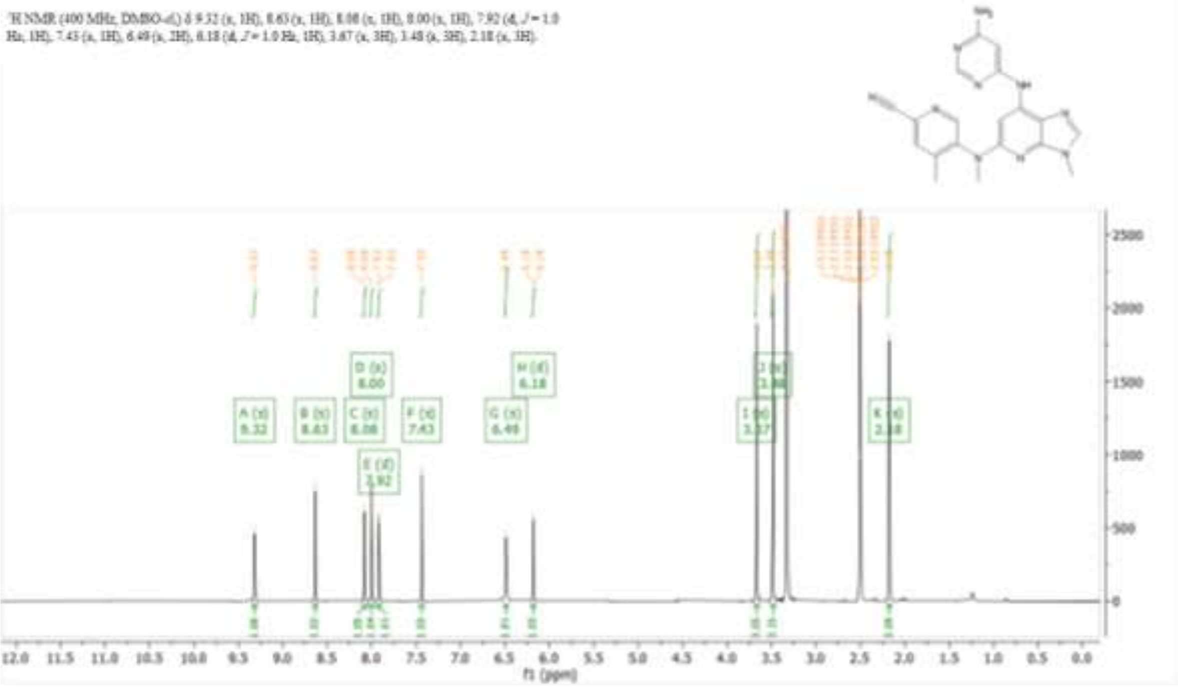

^{13}C NMR of compound **20**

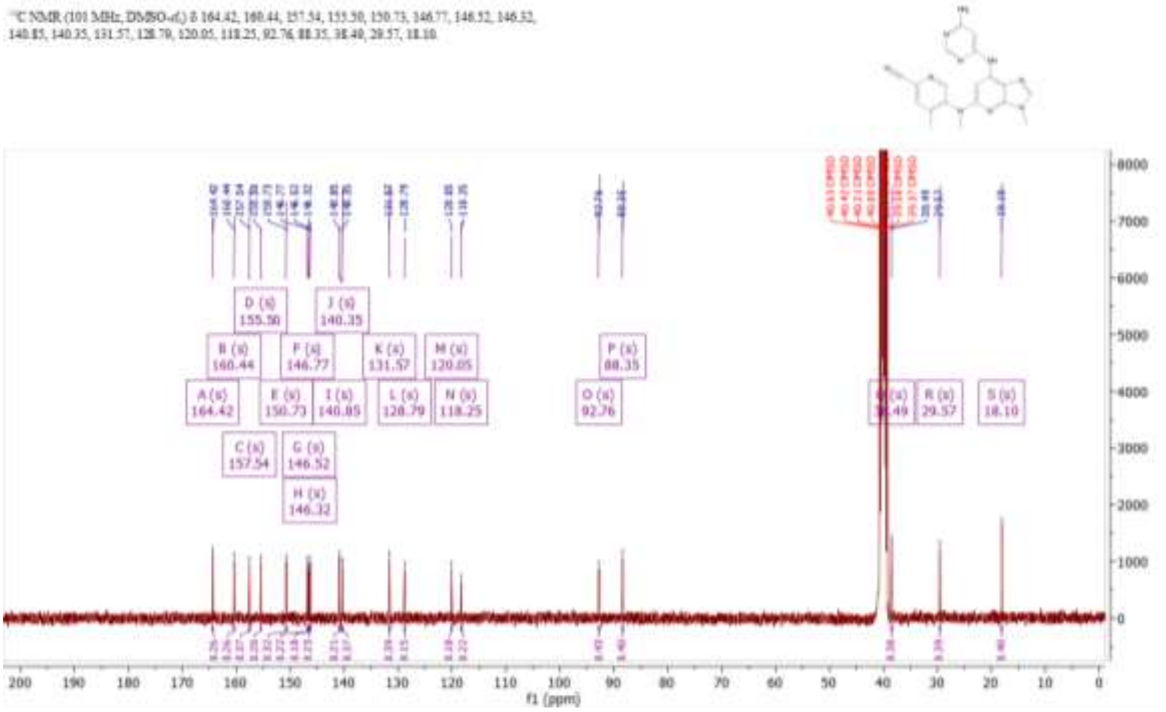

Chromatogram of compound **20**

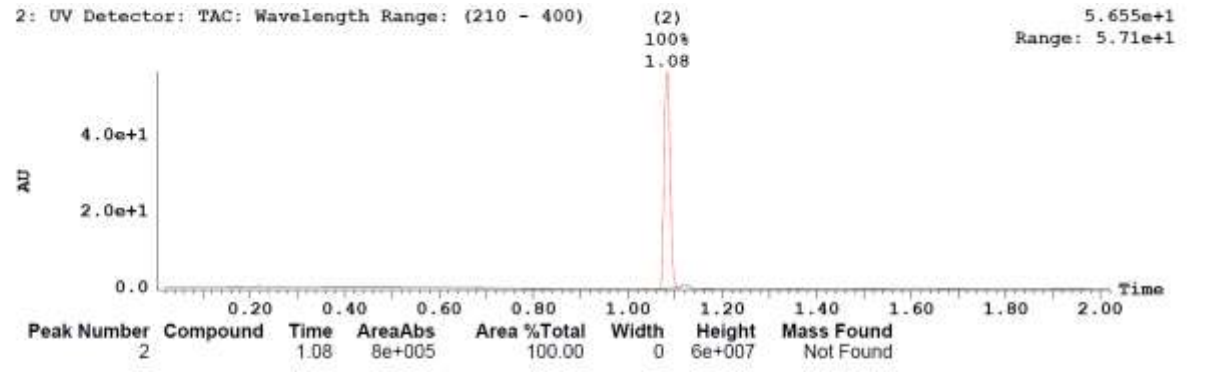

Mass spectrum of compound **20**

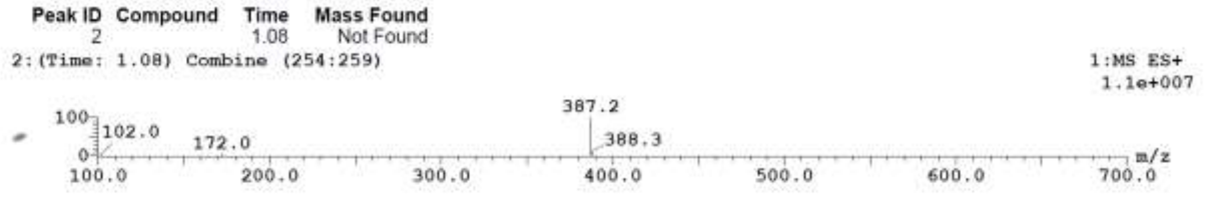

Analytical Data for 21.

^{1}H NMR of compound **21**

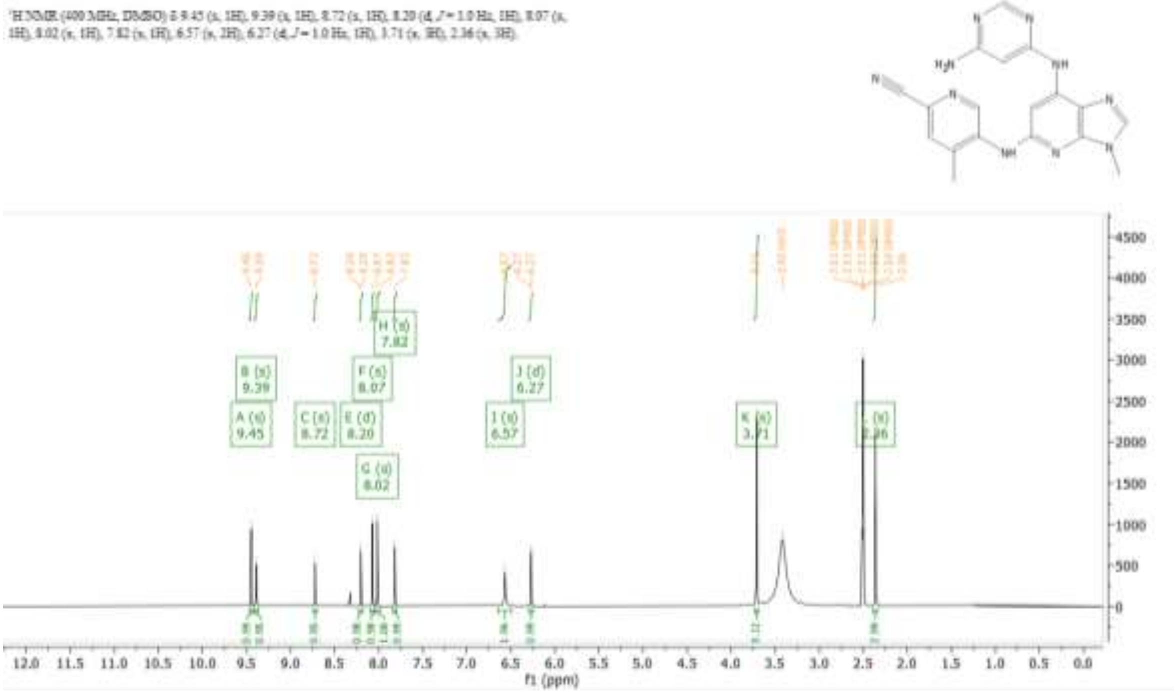

Chromatogram of compound **21**

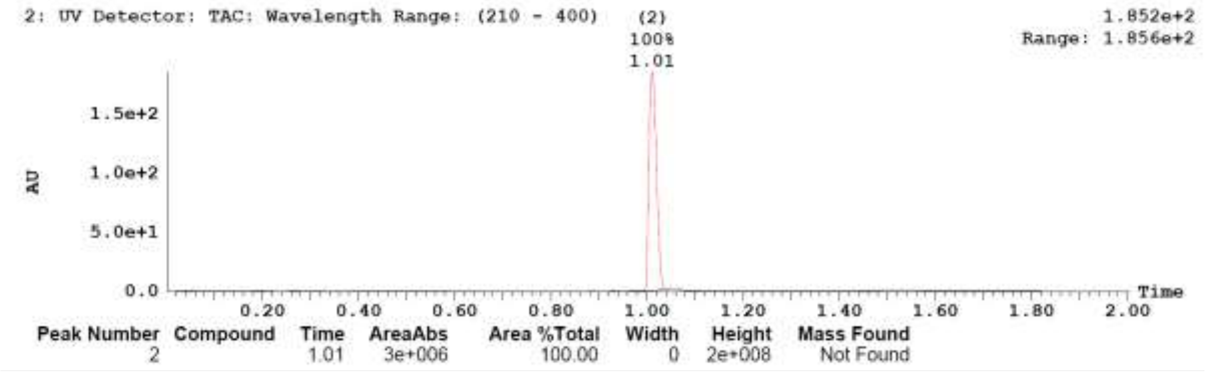

Mass spectrum of compound **21**

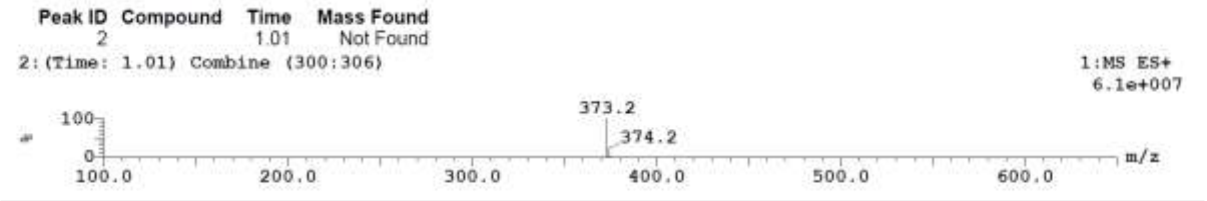

Analytical Data for 22.

^{1}H NMR of compound **22**

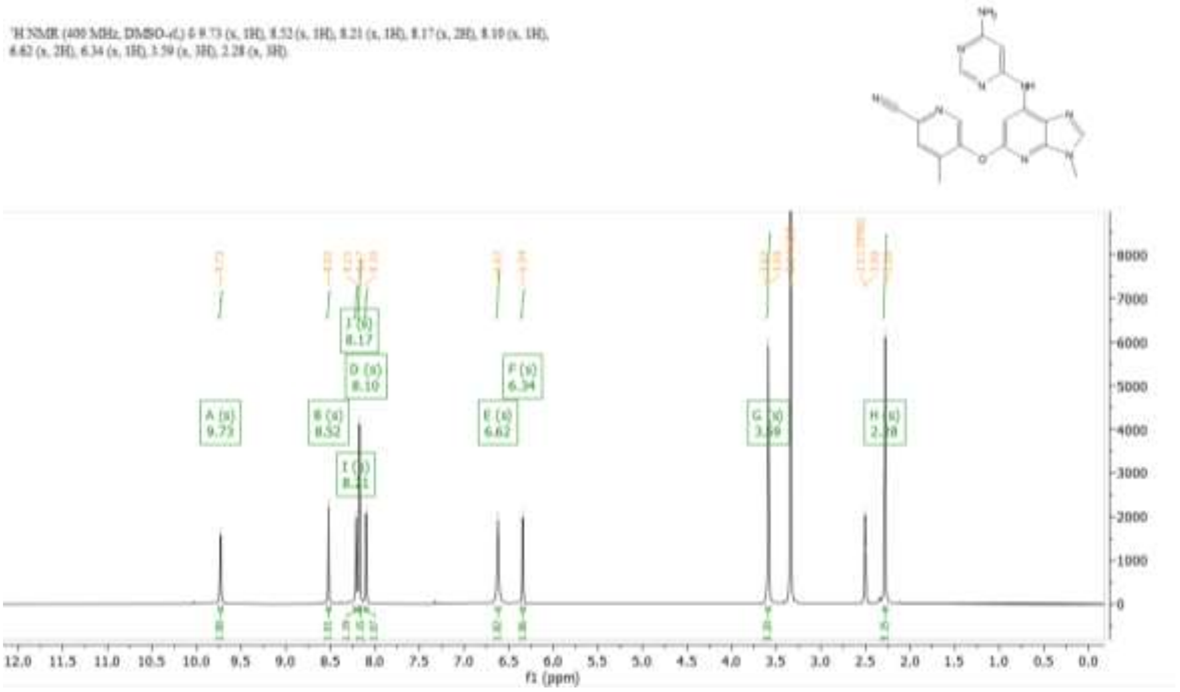

^{13}C NMR of compound **22**

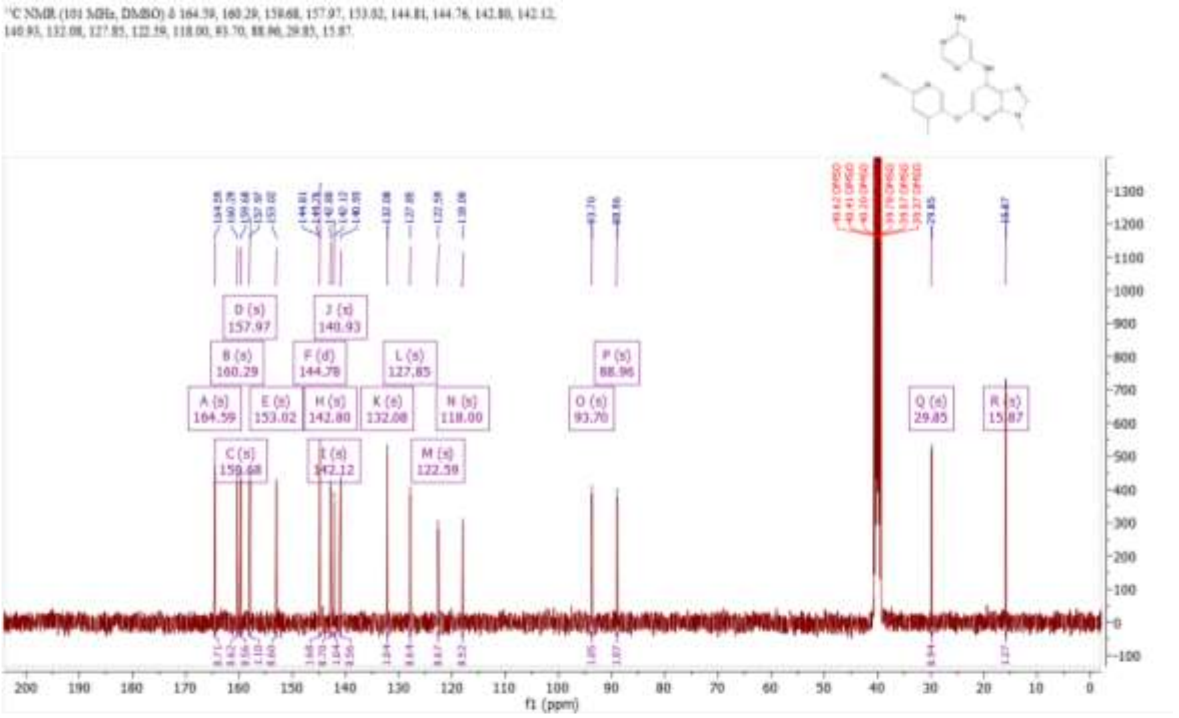

Chromatogram of compound **22**

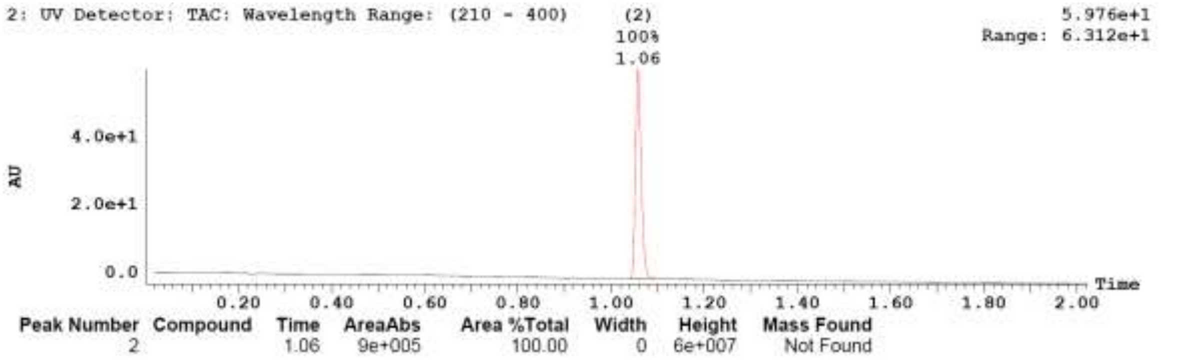

Mass spectrum of compound **22**

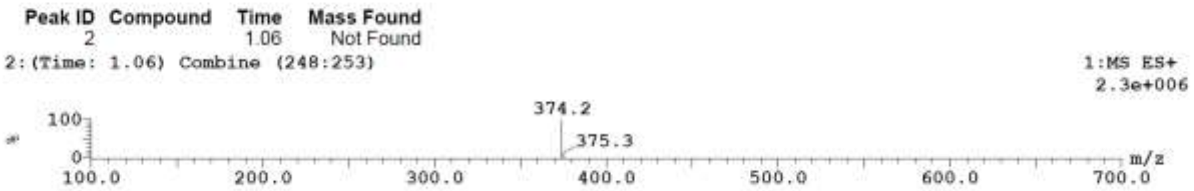

Analytical Data for 23.

^{1}H NMR of compound **23**

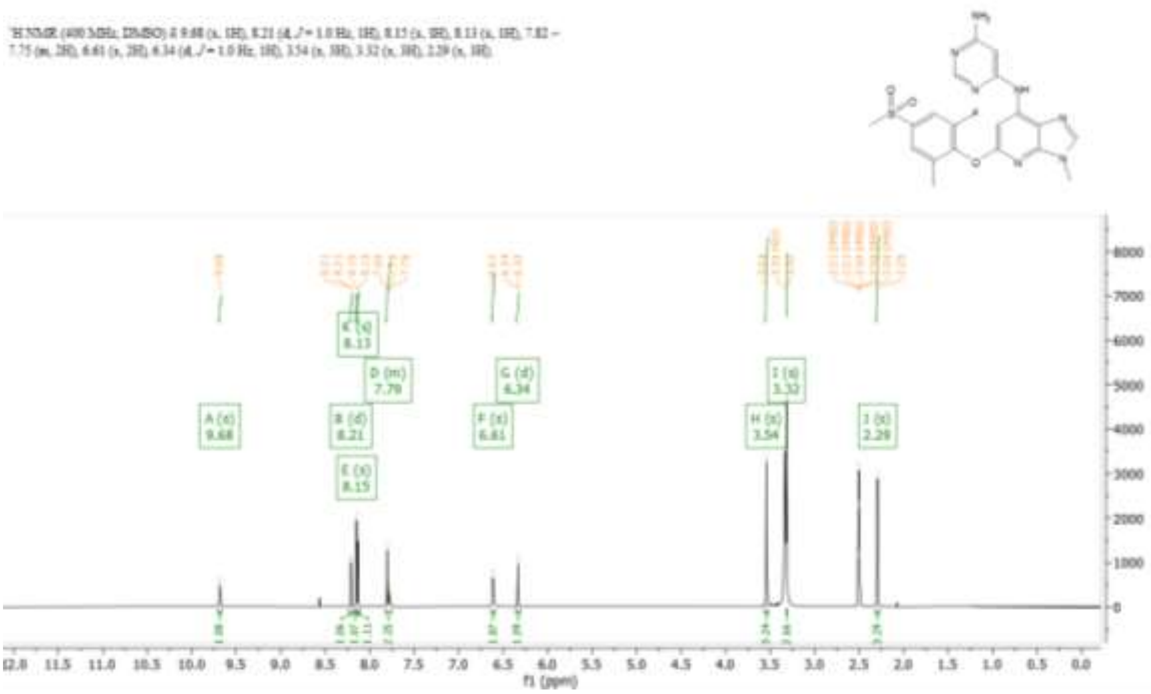

^{13}C NMR of compound **23**

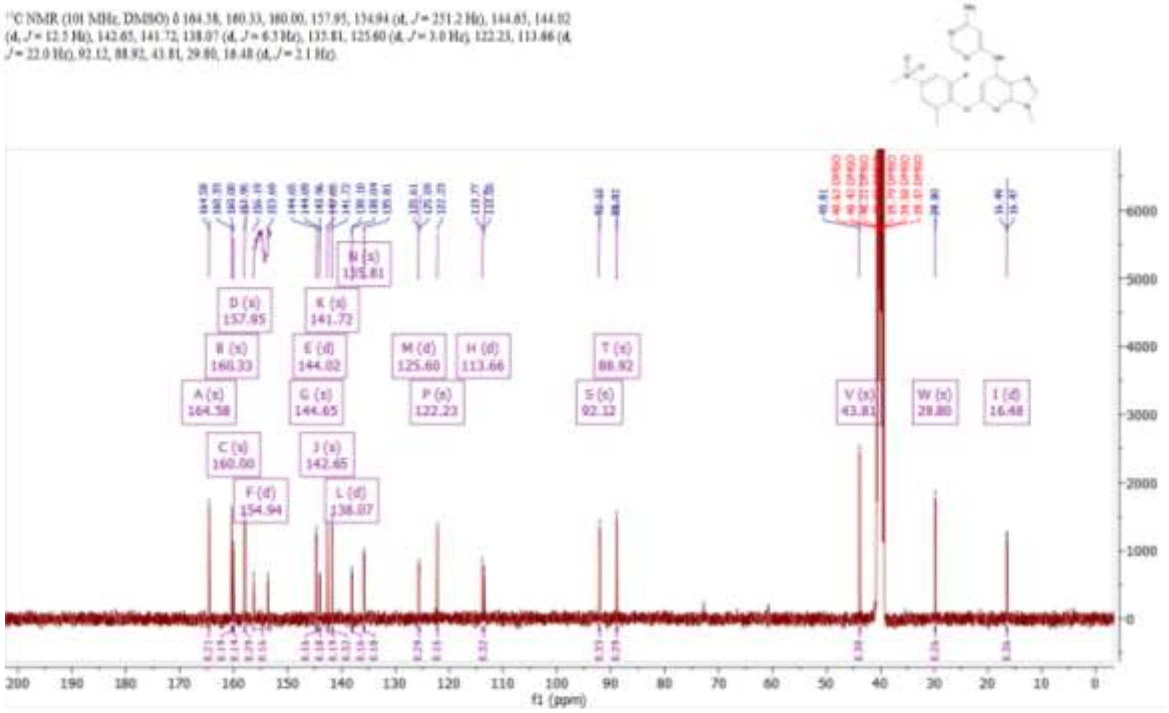

Chromatogram of compound **23**

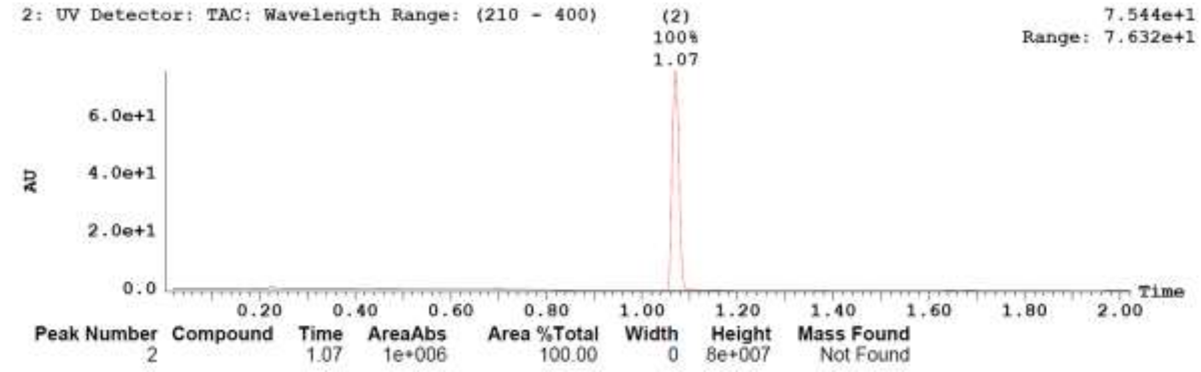

Mass spectrum of compound **23**

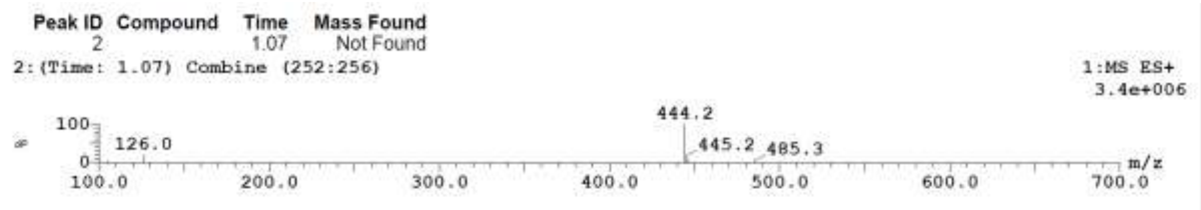

Analytical Data for 24.

^{1}H NMR of compound **24**

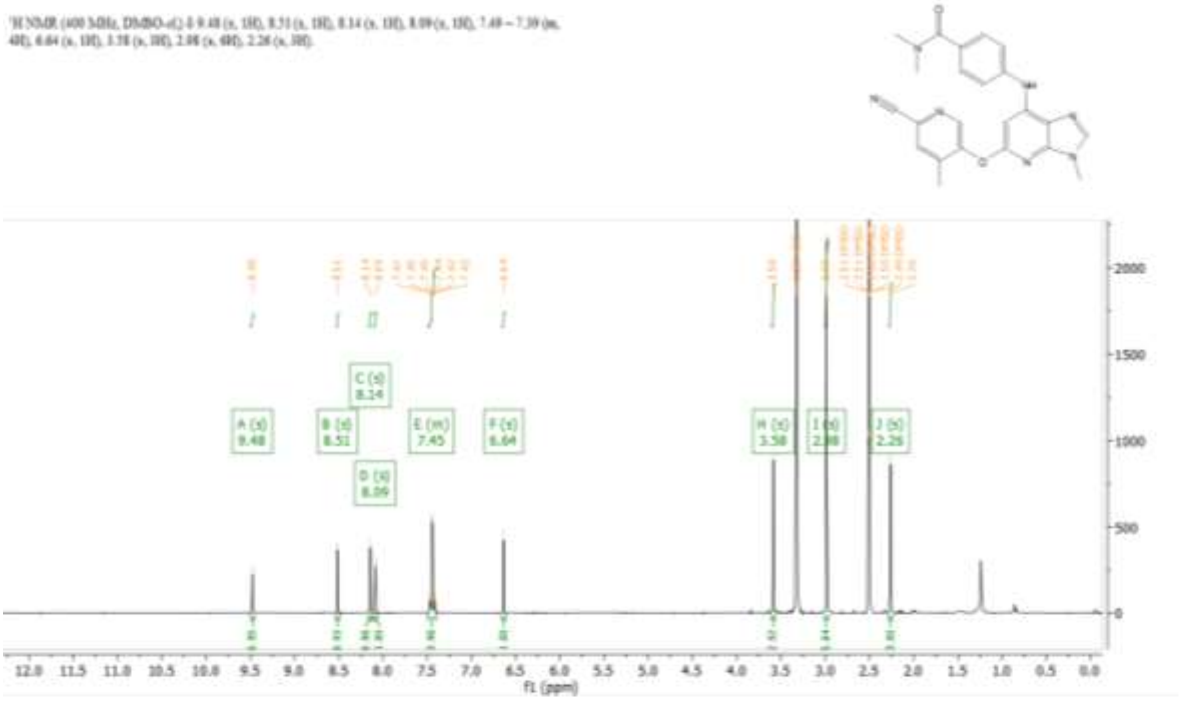

^{13}C NMR of compound **24**

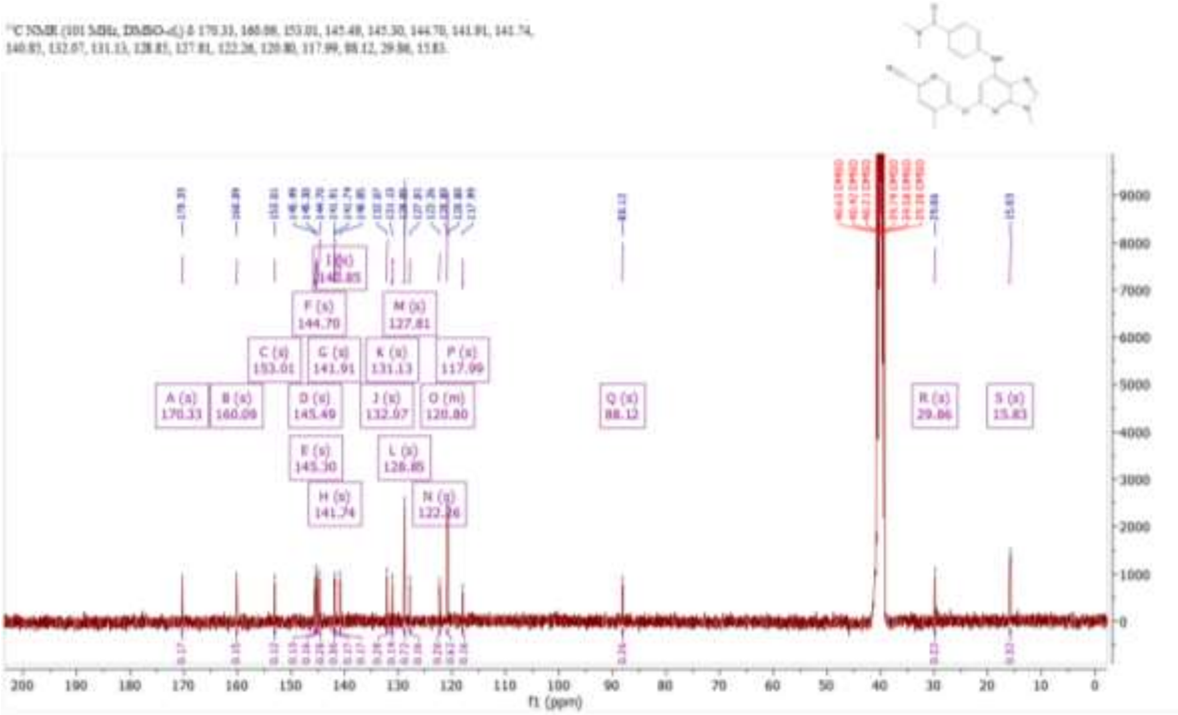

Chromatogram of compound **24**

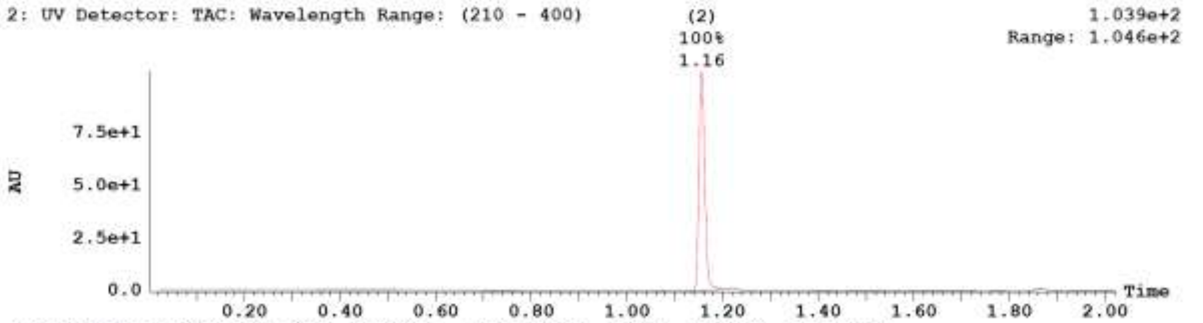

Peak Number	Compound	Time	AreaAbs	Area %Total	Width	Height	Mass Found
2		1.16	1e+006	100.00	0	1e+008	Not Found

Mass spectrum of compound **24**

Peak ID	Compound	Time	Mass Found
2		1.16	Not Found

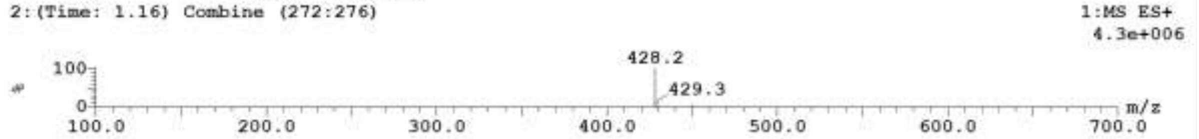

Analytical Data for 25.

^{1}H NMR of compound **25**

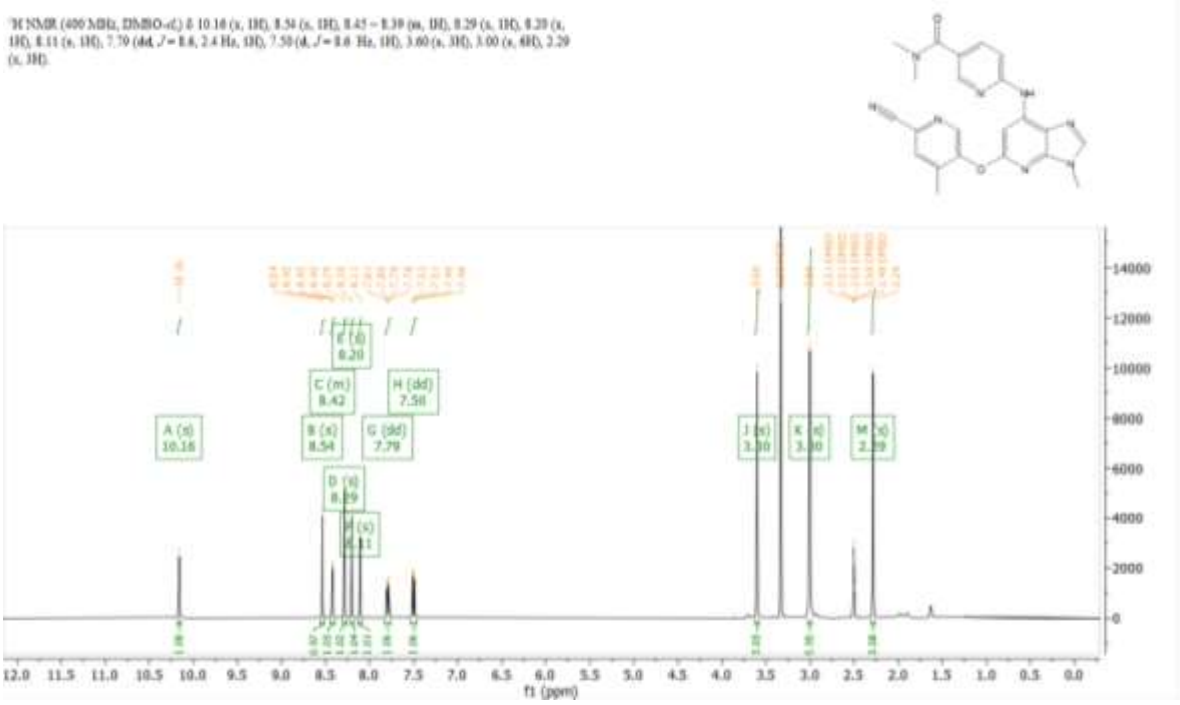

^{13}C NMR of compound **25**

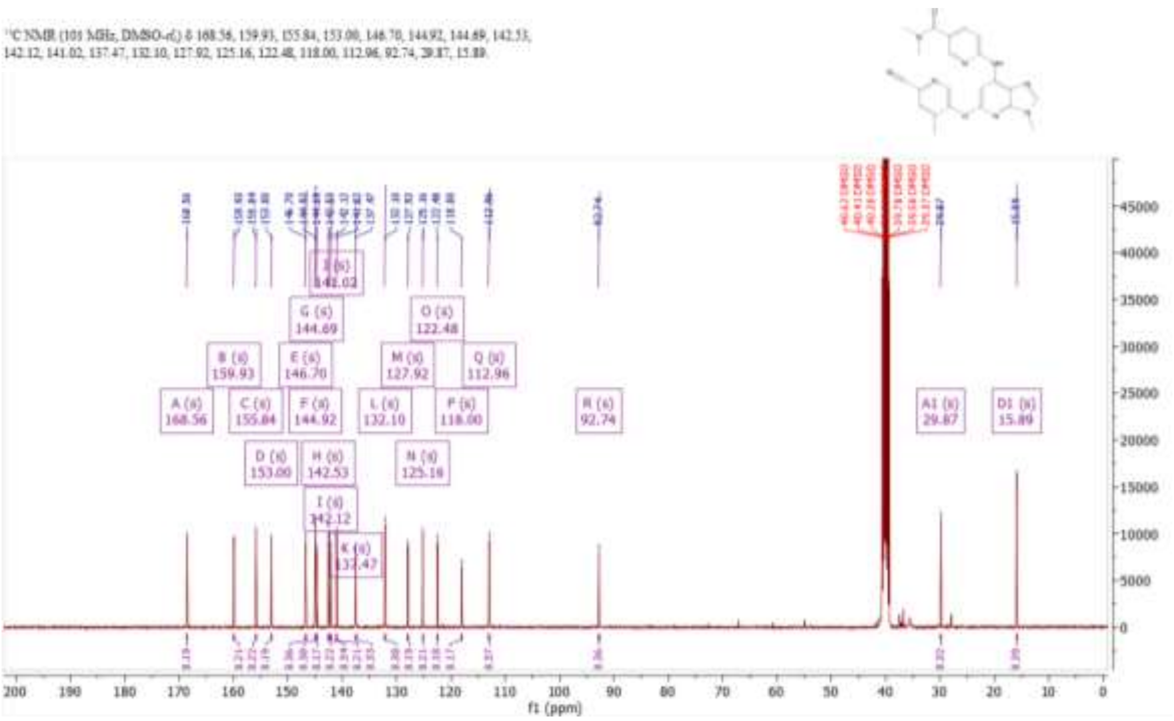

Chromatogram of compound **25**

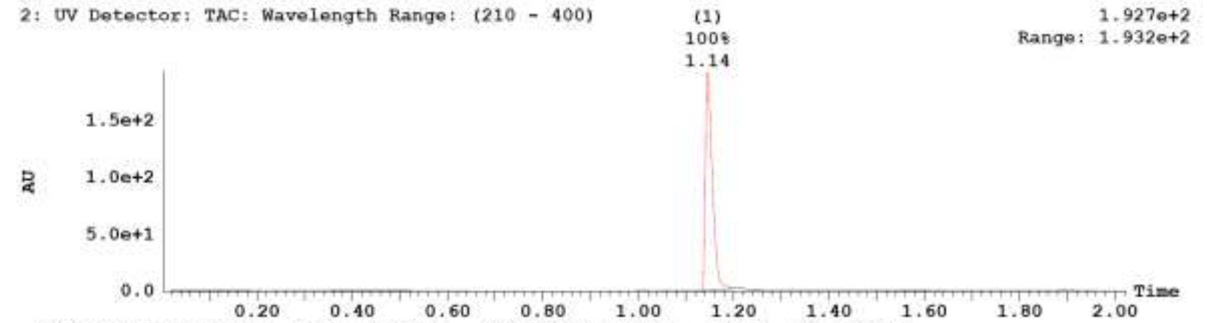

Peak Number	Compound	Time	AreaAbs	Area %Total	Width	Height	Mass Found
1		1.14	3e+006	100.00	0	2e+008	Not Found

Mass spectrum of compound **25**

Peak ID	Compound	Time	Mass Found
1		1.14	Not Found

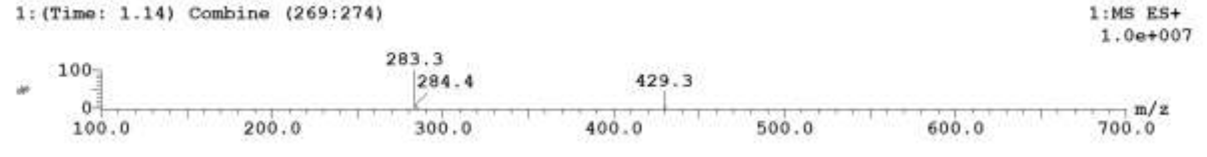

Analytical Data for 26.

^{1}H NMR of compound **26**

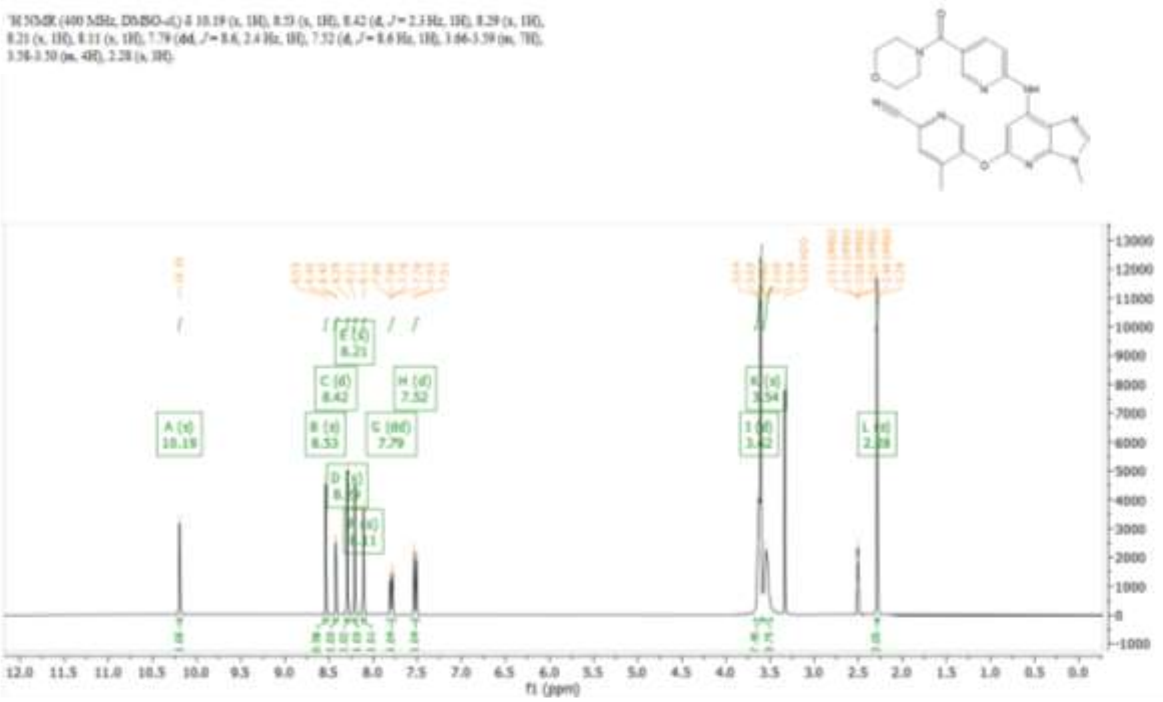

^{13}C NMR of compound **26**

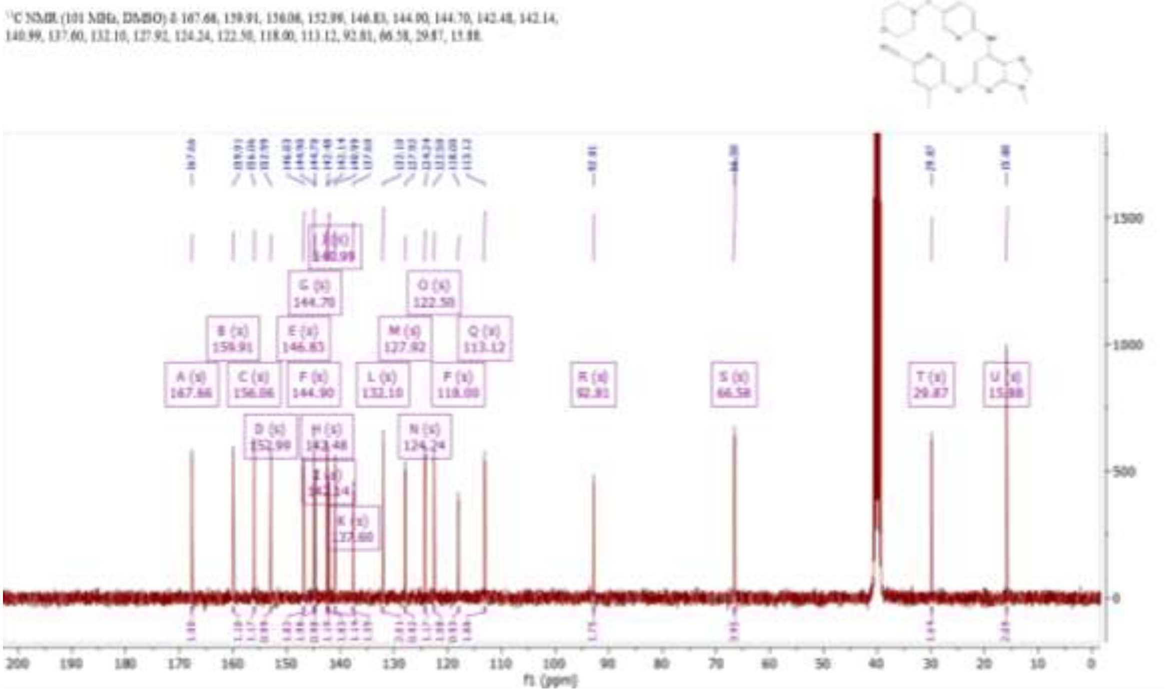

Chromatogram of compound **26**

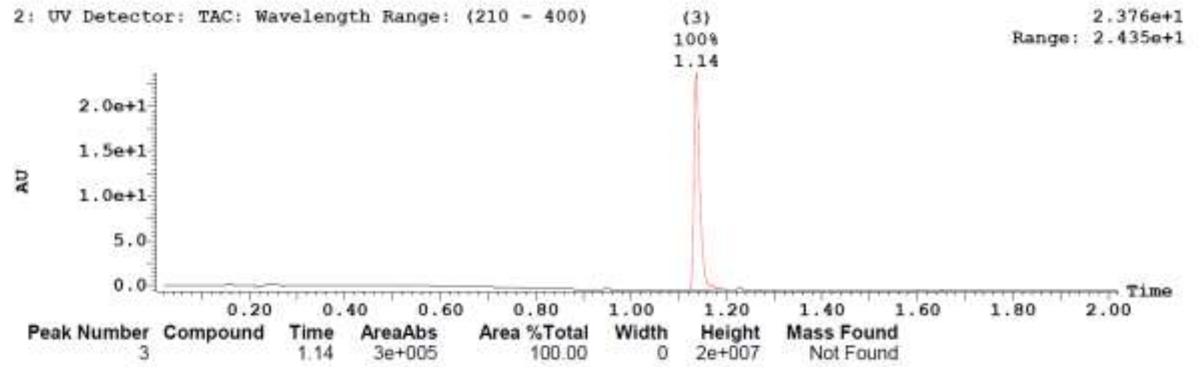

Mass spectrum of compound **26**

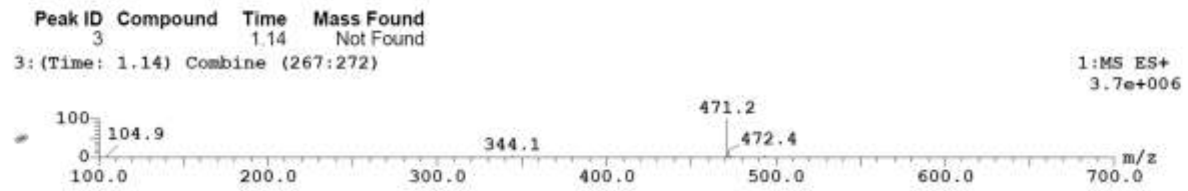

Analytical Data for 27.

^{1}H NMR of compound **27**

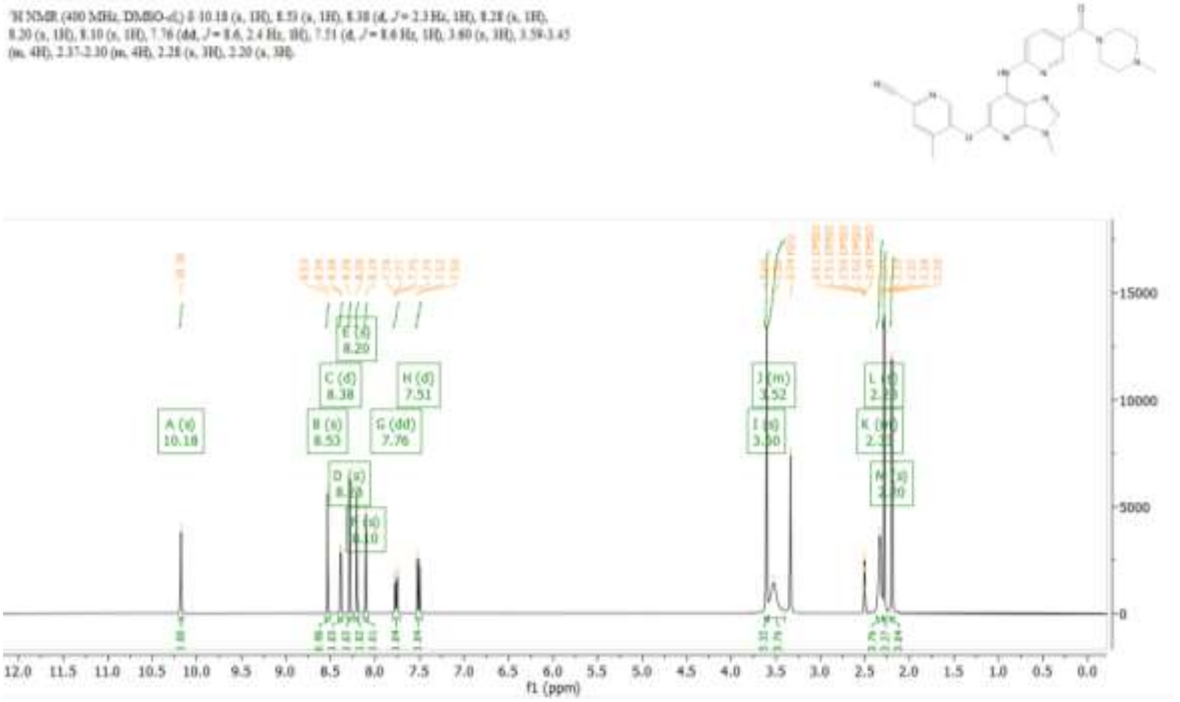

^{13}C NMR of compound **27**

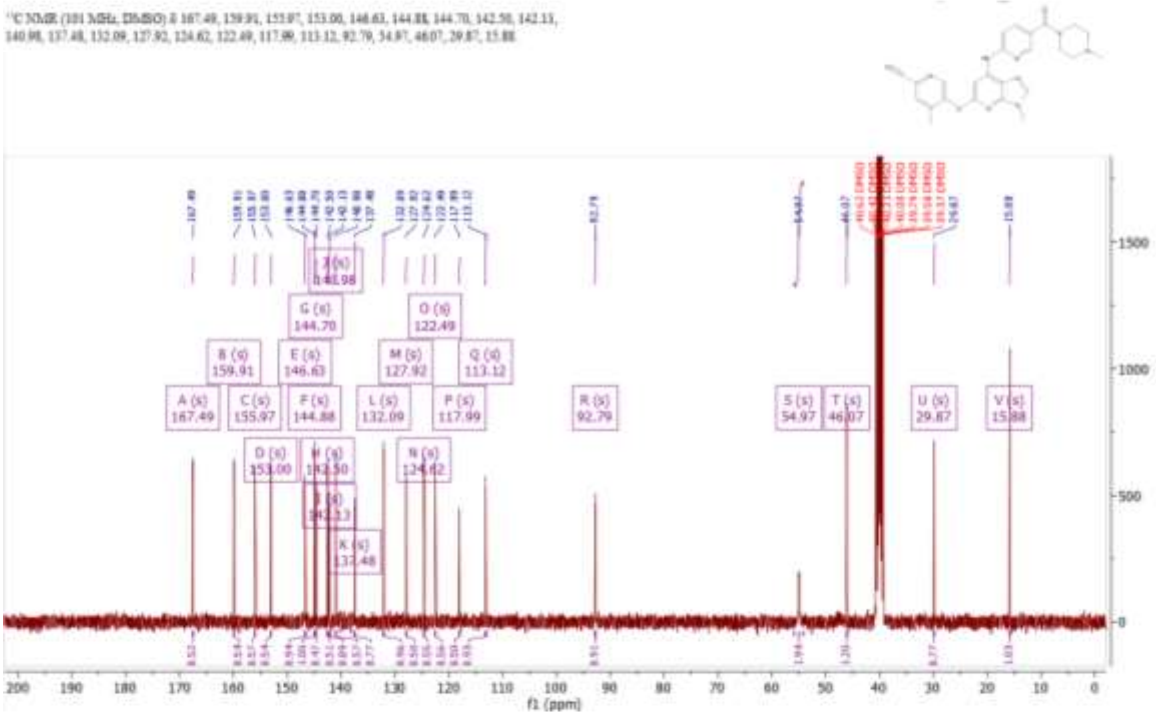

Chromatogram of compound **27**

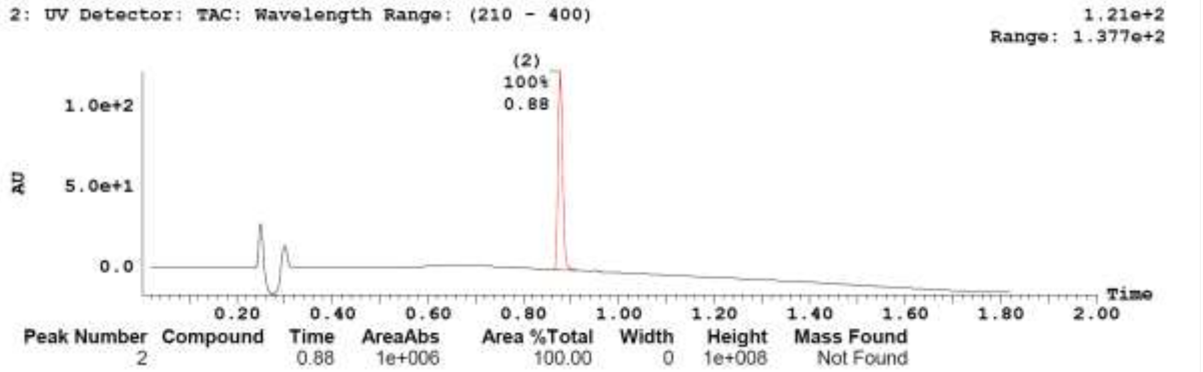

Mass spectrum of compound **27**

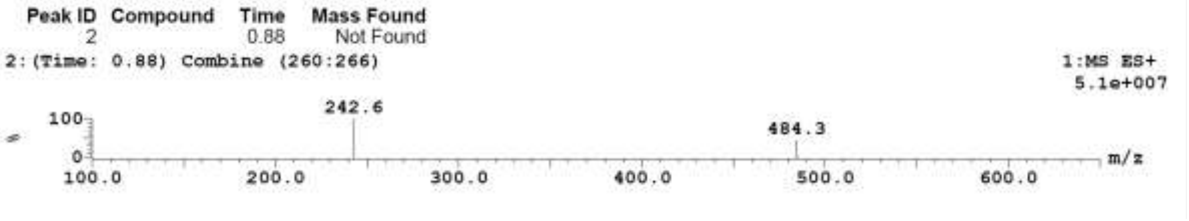

Analytical Data for 28.

^{1}H NMR of compound **28**

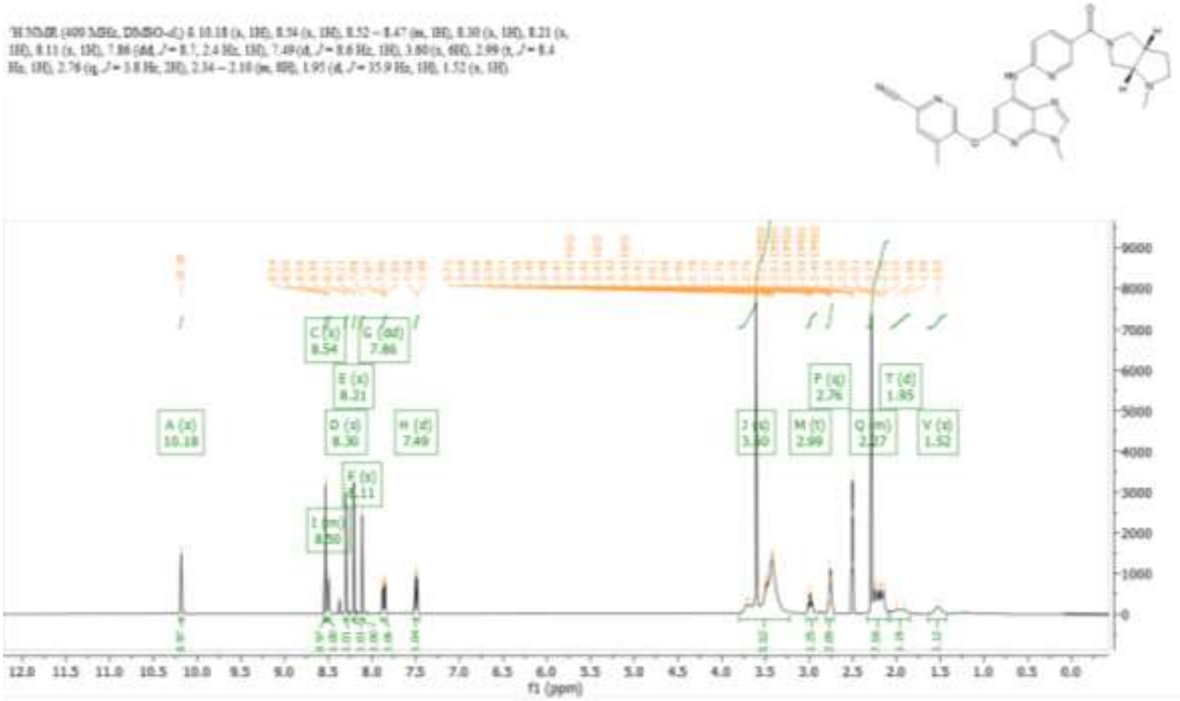

Chromatogram of compound **28**

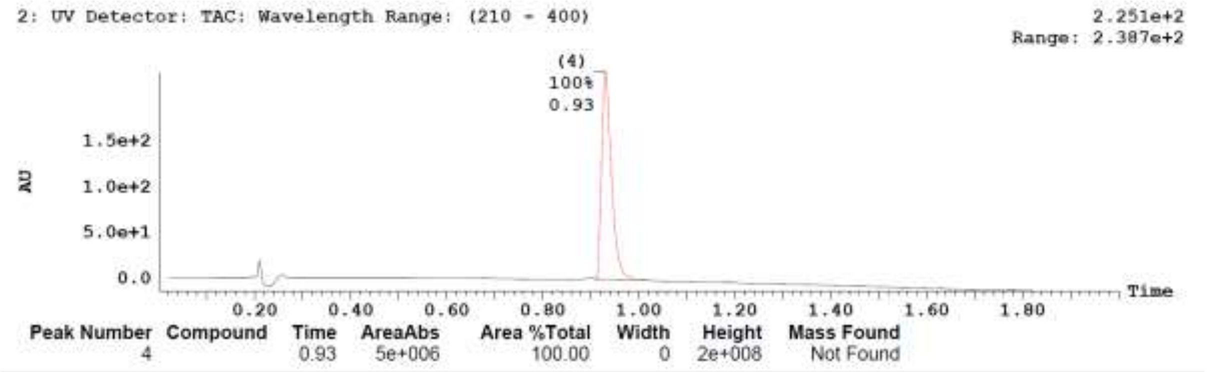

Mass spectrum of compound **28**

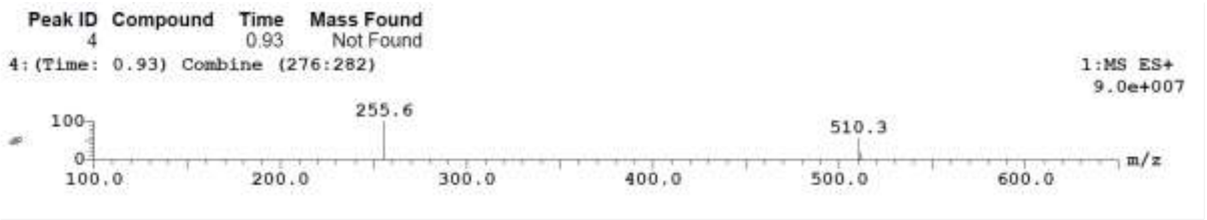

Analytical Data for 29.

^{1}H NMR of compound **29**

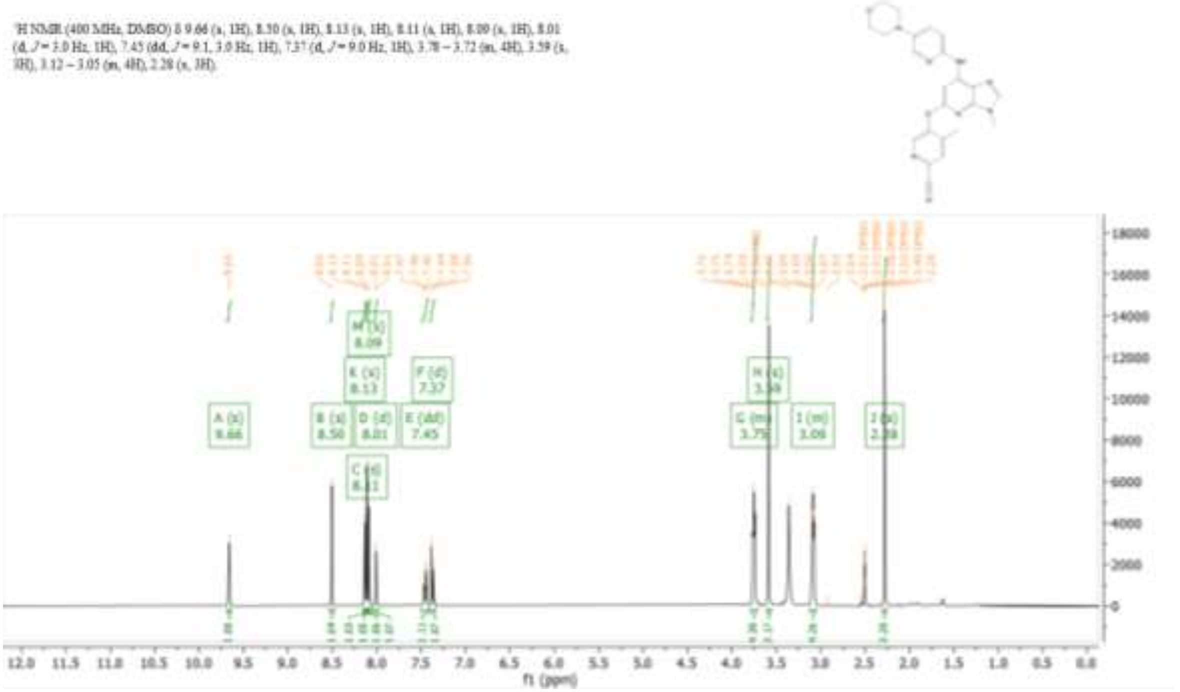

^{13}C NMR of compound **29**

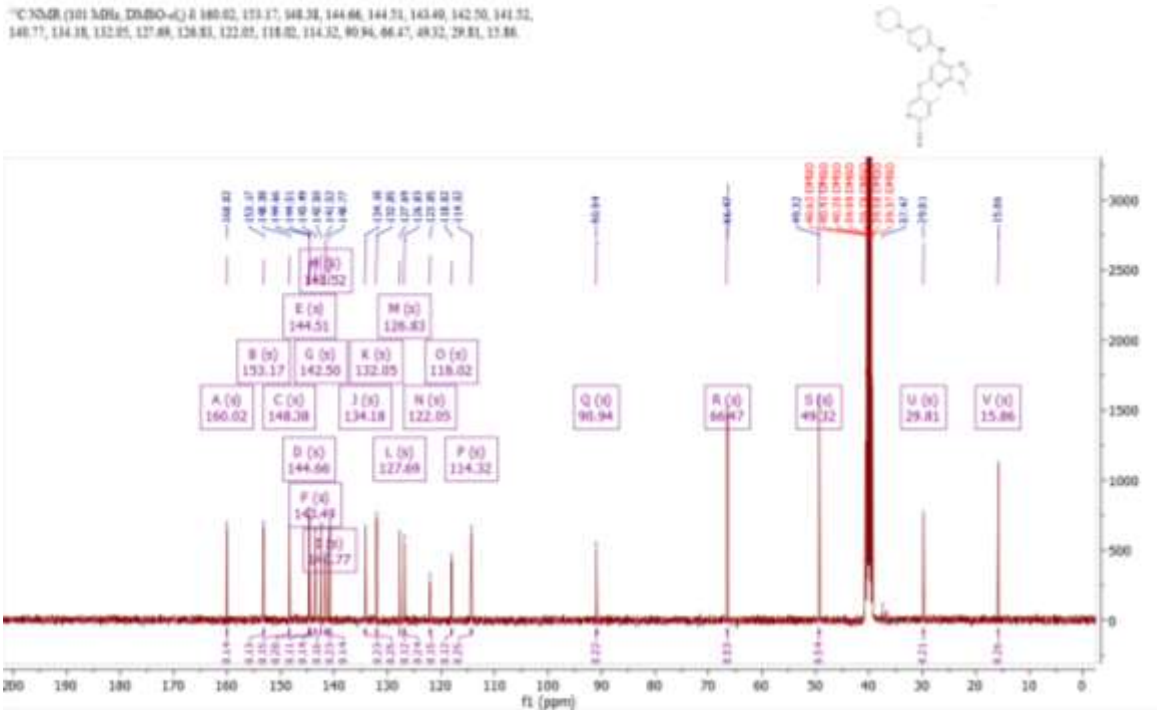

Chromatogram of compound **29**

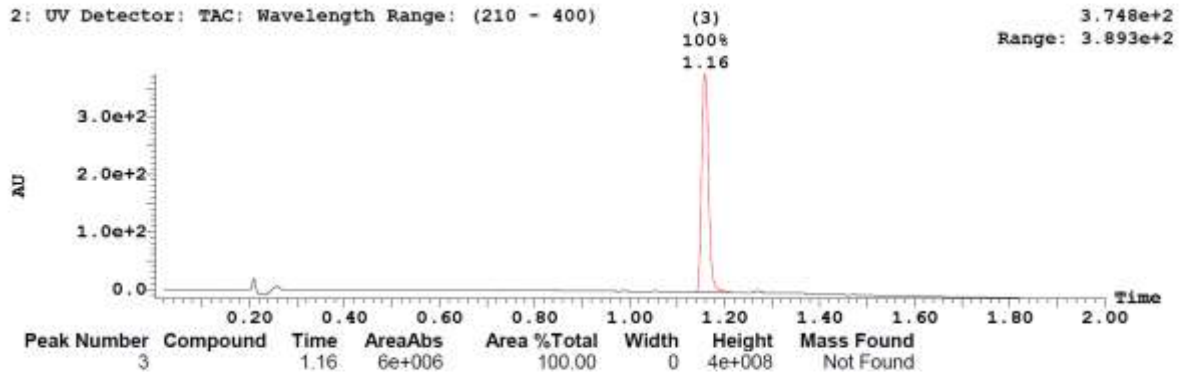

Mass spectrum of compound **29**

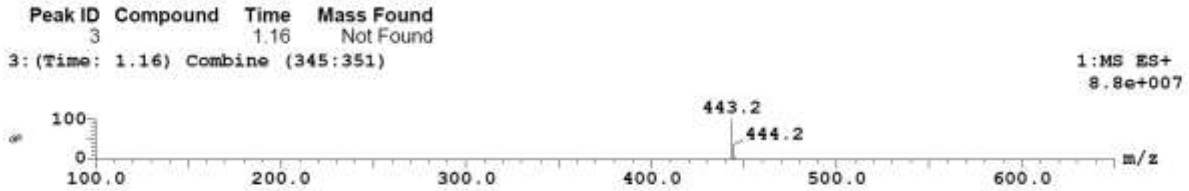

Analytical Data for 9.

^{1}H NMR of compound **9**

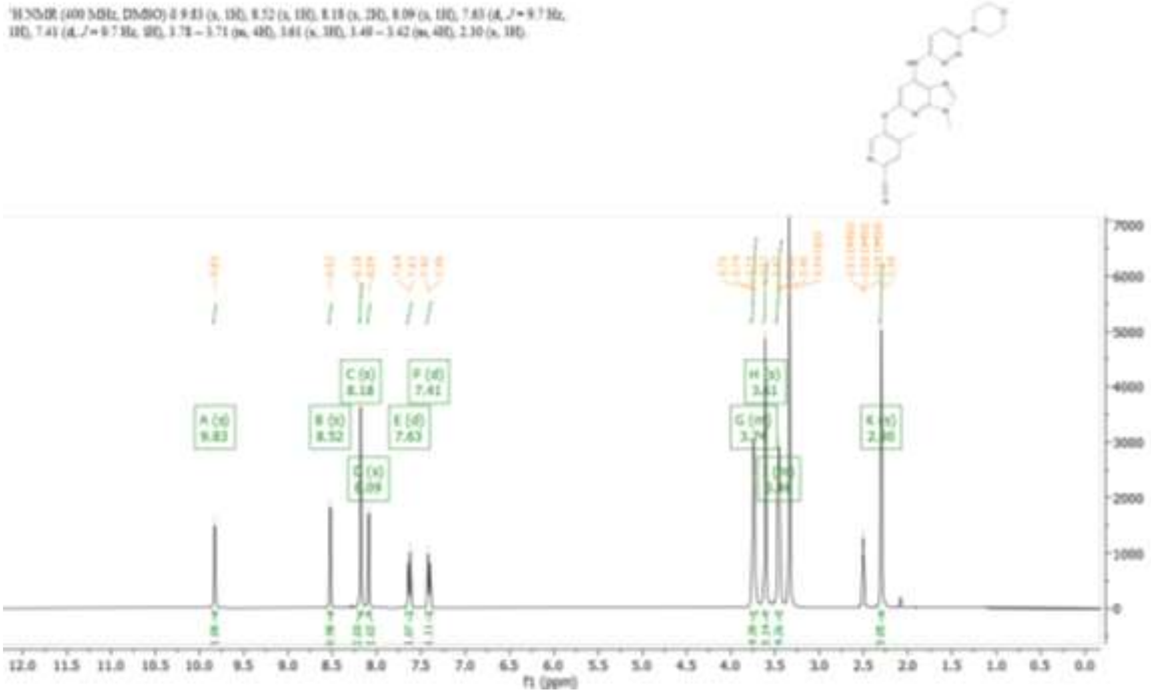

^{13}C NMR of compound **9**

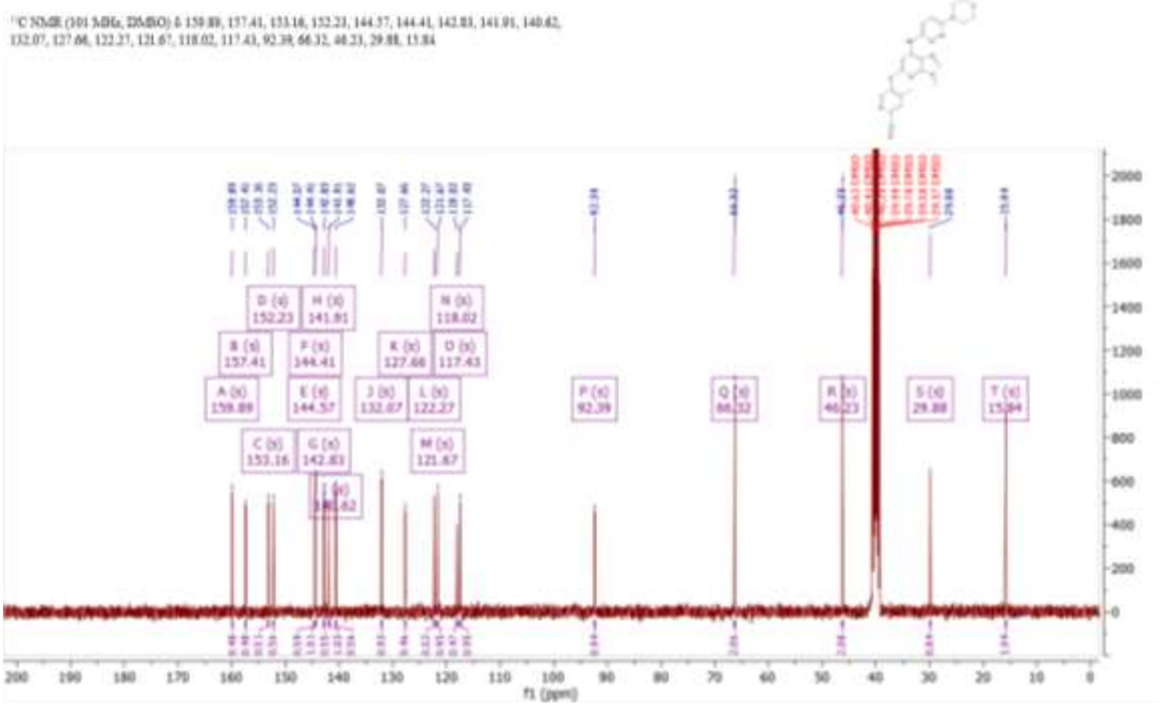

Chromatogram of compound **9**

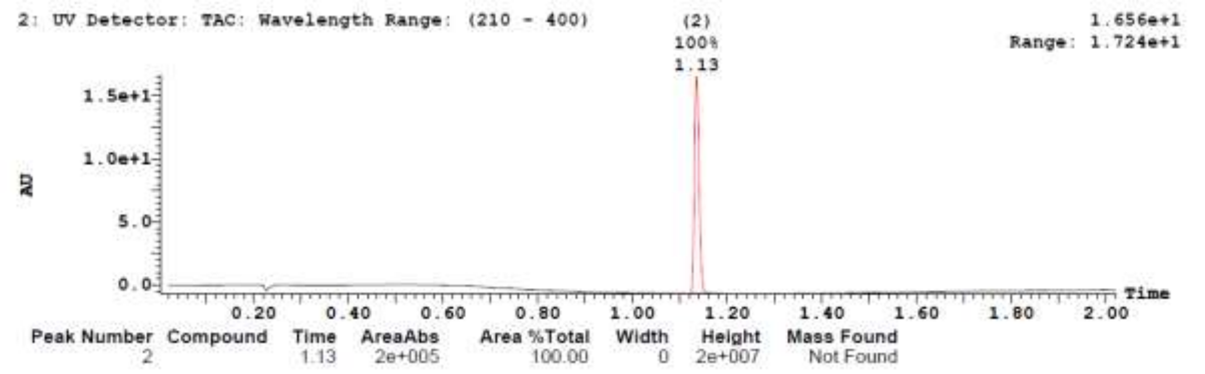

Mass spectrum of compound **9**

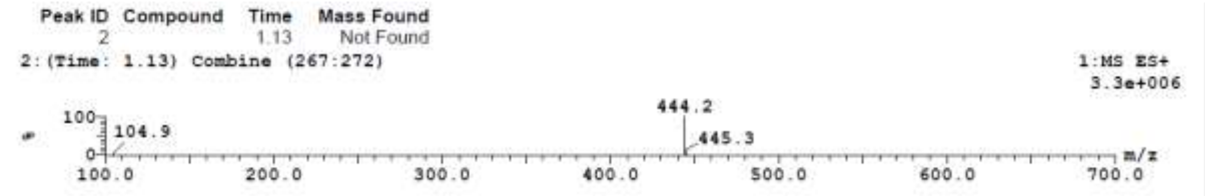

Analytical Data for 30.

^{1}H NMR of compound **30**

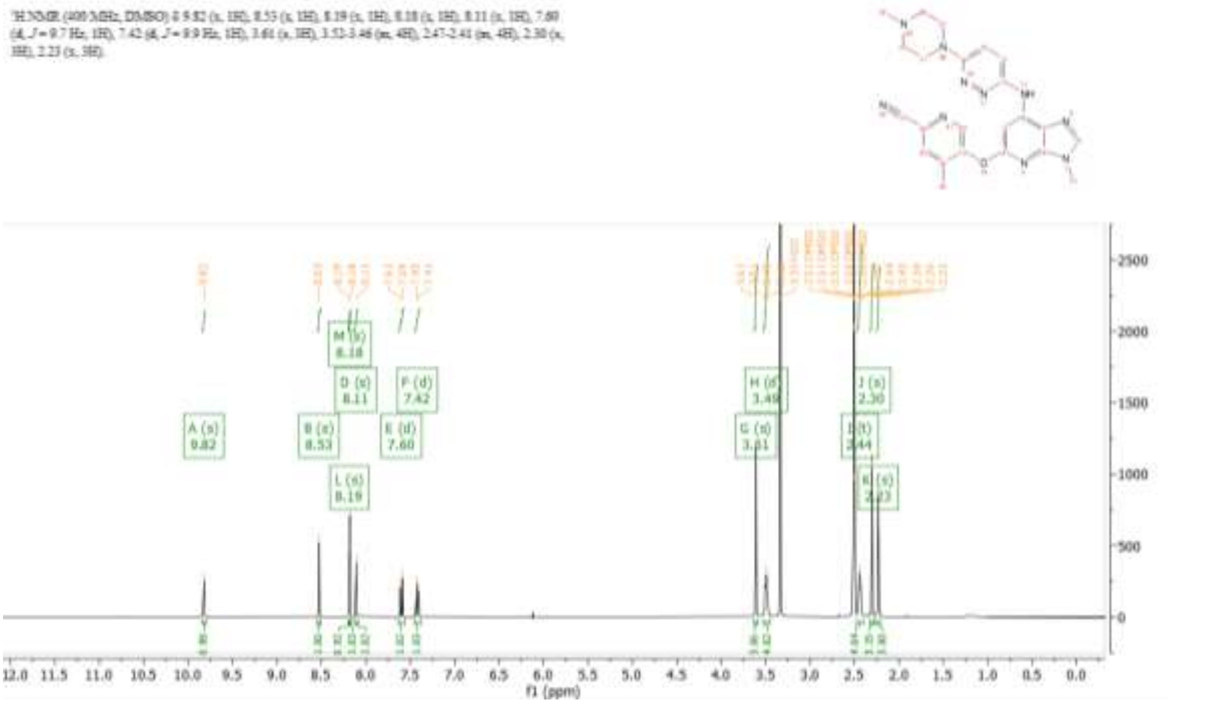

Chromatogram of compound **30**

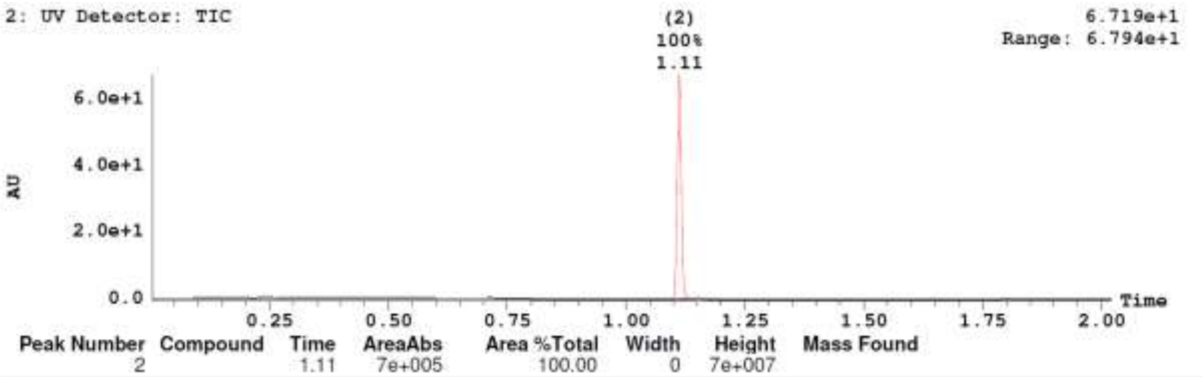

Mass spectrum of compound **30**

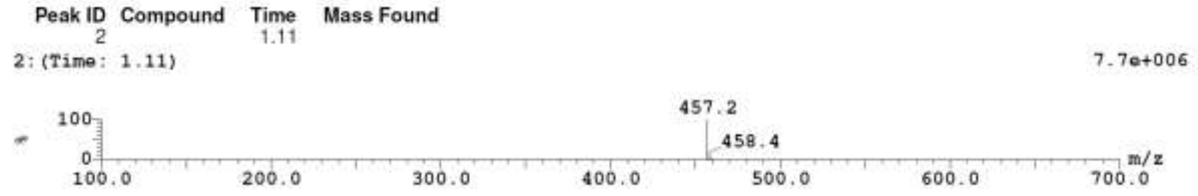

Analytical Data for 31.

^{1}H NMR of compound **31**

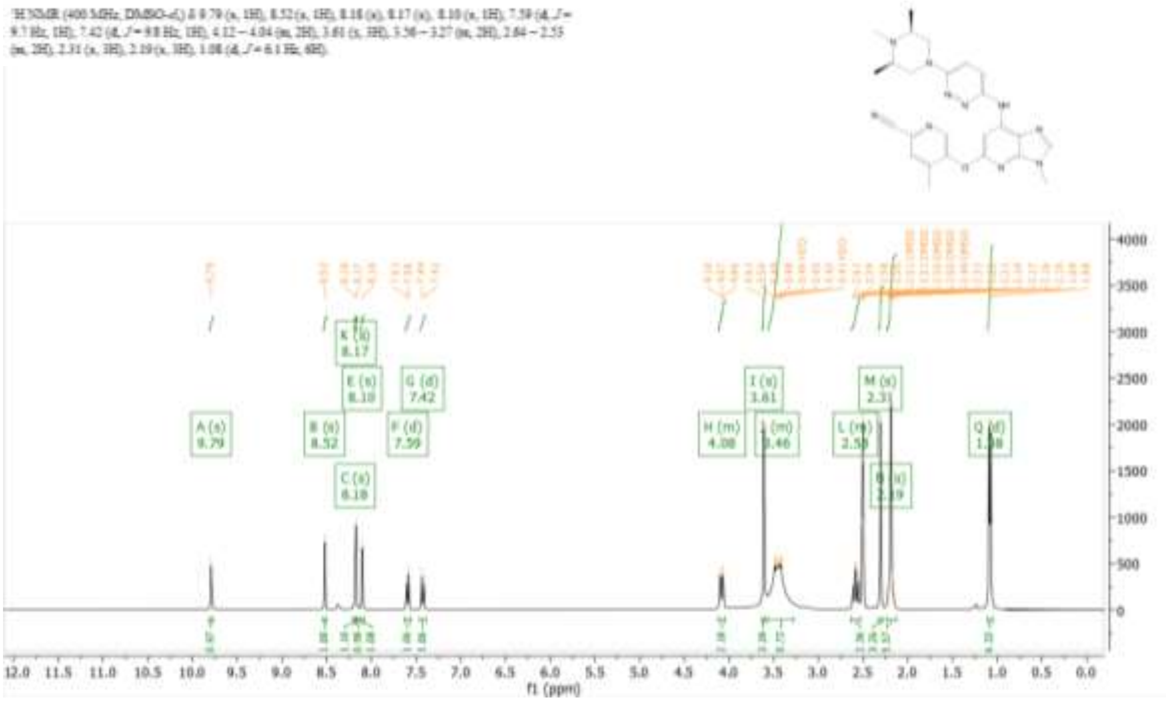

^{13}C NMR of compound **31**

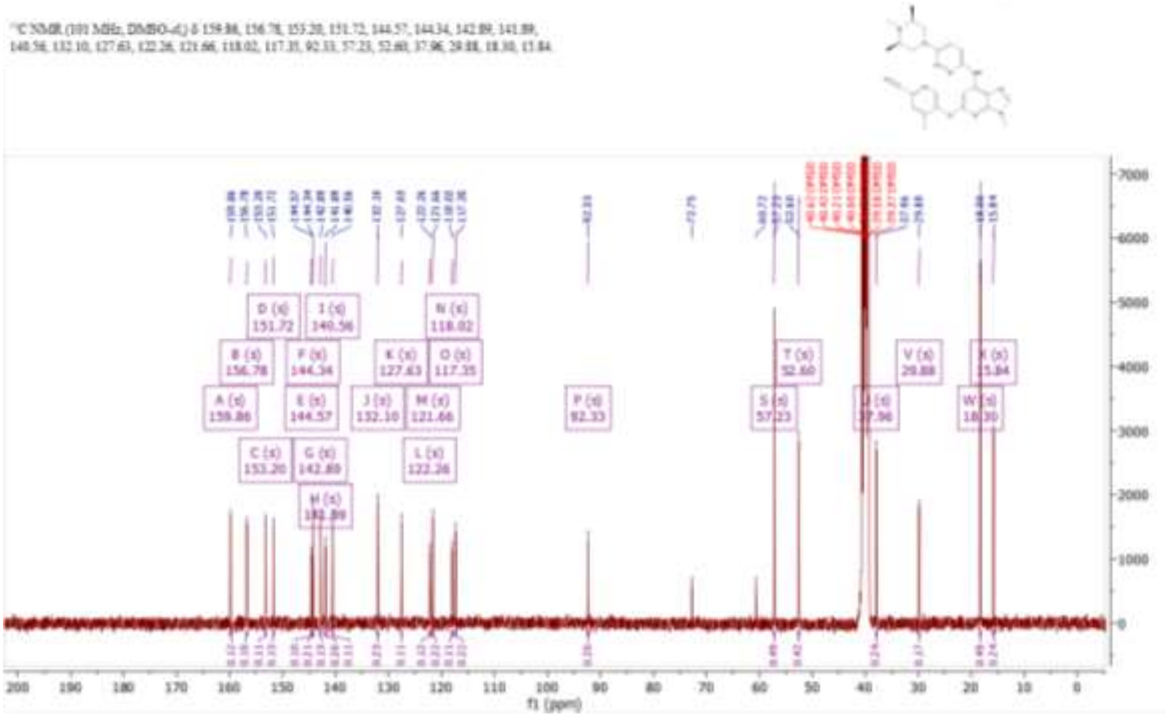

Chromatogram of compound **31**

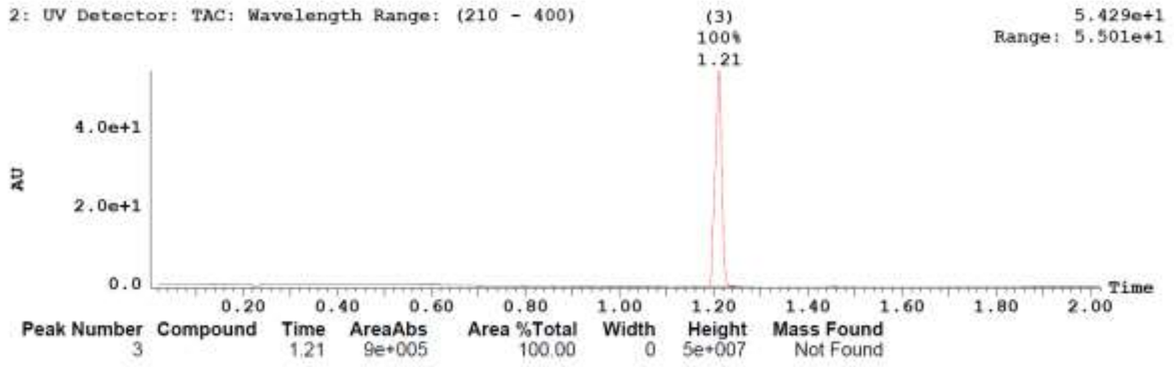

Mass spectrum of compound **31**

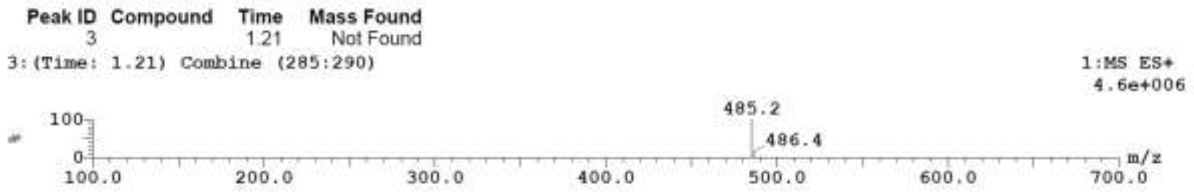

Molecular Formula Strings List.

The molecular formula strings list (CSV) is available free of charge on the ACS Publications website.

Compounds SMILE.

Compound	SMILE	TYK2 IC_{50} (nM)
11	CN(C=N1)C2=C1C(NC(C3CC3)=O)=CC(N(C)C4=C(F)C=C(C#N)C=C4CC)=N2	0.72
12	CCC1=CC(C#N)=CC(F)=C1N(C2=CC(NC(NC)=O)=C3N=CN(C3=N2)C)C	0.32
13	CN1C2=NC(N(C3=C(F)C=C(C#N)C=C3CC)C)=CC(NC4=CC(N)=NC=N4)=C2N=C1	3.91
14	CN1C2=NC(N(C)[C@@H]3CC[C@@H](C#N)CC3)=CC(NC4=CC(N)=NC=N4)=C2N=C1	6
15	CN1C2=NC(N(C)C3CCOC4(CCC4)C3)=CC(NC5=CC(N)=NC=N5)=C2N=C1	7.5
16	CN1C2=NC(N(C)C(C3CC3)C(F)(F)F)=CC(NC4=CC(N)=NC=N4)=C2N=C1	0.96

17	CN1C(N=C2NC(C(F)(F)F)C3CC3)=C(C(NC4=NC=NC(N)=C4)=C2)N=C1	1
18	CN1C2=NC(OC(C3CC3)C(F)(F)F)=CC(NC4=NC=NC(N)=C4)=C2N=C1	1.2
19	CN1C2=NC(N(C3=C(CC)C=C(C#N)N=C3)C)=CC(NC4=CC(N)=NC=N4)=C2N=C1	0.42
20	CN1C2=NC(N(C)C3=C(C)C=C(C#N)N=C3)=CC(NC4=CC(N)=NC=N4)=C2N=C1	0.96
21	CC1=C(NC2=NC(N(C)C=N3)=C3C(NC4=CC(N)=NC=N4)=C2)C=NC(C#N)=C1	4.9
22	CN1C2=NC(OC3=CN=C(C#N)C=C3C)=CC(NC4=CC(N)=NC=N4)=C2N=C1	2.7
23	CN1C2=NC(OC3=C(F)C=C(S(C)(=O)=O)C=C3C)=CC(NC4=CC(N)=NC=N4)=C2N=C1	4.6
24	CN1C2=NC(OC3=CN=C(C#N)C=C3C)=CC(NC4=CC=C(C(N(C)C)=O)C=C4)=C2N=C1	12
25	CN1C2=NC(OC3=CN=C(C#N)C=C3C)=CC(NC4=CC=C(C(N(C)C)=O)C=N4)=C2N=C1	3.6
26	CN1C2=NC(OC3=CN=C(C#N)C=C3C)=CC(NC4=CC=C(C(N5CCOCC5)=O)C=N4)=C2N=C1	1.6
27	O=C(N1CCN(C)CC1)C2=CC=C(NC3=C4C(N(C)C=N4)=NC(OC5=CN=C(C#N)C=C5C)=C3)N=C2	1.8
28	O=C(N1C[C@]2([H])CCN(C)[C@]2([H])C1)C3=CC=C(NC4=C5C(N(C)C=N5)=NC(OC6=CN=C(C#N)C=C6C)=C4)N=C3	2.1
29	CN1C2=NC(OC3=CN=C(C#N)C=C3C)=CC(NC4=CC=C(N5CCOCC5)C=N4)=C2N=C1	3.6
9	CN1C=NC2=C(NC3=CC=C(N4CCOCC4)N=N3)C=C(OC5=C(C)C=C(C#N)N=C5)N=C21	2.3
30	CC1=CC(C#N)=NC=C1OC2=CC(NC3=NN=C(N4CCN(CC4)C)C=C3)=C5C(N(C)C=N5)=N2	2.4
31	CN1C2=NC(OC3=CN=C(C#N)C=C3C)=CC(NC4=CC=C(N5C[C@H](C)N(C)[C@H](C)C5)N=N4)=C2N=C1	3.3

REFERENCE

(1) Berman, H. M.; Westbrook, J.; Feng, Z.; Gilliland, G.; Bhat, T. N.; Weissig, H.; Shindyalov, I. N.; Bourne, P. E. The Protein Data Bank. *Nucleic Acids Res.* **2000**, *28* (1), 235–242. DOI: 10.1093/nar/28.1.235
